# Transcriptomics Coupled to Proteomics Reveals Novel Targets for the Protective Role of Spermine in Diabetic Cardiomyopathy

**DOI:** 10.1155/2022/5909378

**Published:** 2022-04-09

**Authors:** Can Wei, Tao Song, Hui Yuan, Xiaoxue Li, Xinying Zhang, Xiao Liang, Ying Fan

**Affiliations:** ^1^Department of Pathophysiology, Harbin Medical University, Harbin 150086, China; ^2^Department of Oral and Maxillofacial Surgery, First Affiliated Hospital of Harbin Medical University, Harbin 150081, China; ^3^Department of Pathophysiology, Mudanjiang Medical University, Mudanjiang 157011, China; ^4^Department of Cardiovascular, First Affiliated Hospital of Harbin Medical University, Harbin 150081, China

## Abstract

**Background:**

Diabetic cardiomyopathy (DbCM) is the main complication and the cause of high mortality of diabetes. Exploring the transcriptomics and proteomics of DbCM is of great significance for understanding the biology of the disease and for guiding new therapeutic targets for the potential therapeutic effect of spermine (SPM).

**Methods and Results:**

By using a mouse DbCM model, we analyzed the overall transcriptome and proteome of the myocardium, before/after treatment with SPM. The general state and cardiac structure and function changes of each group were also compared. Diabetes induced an increased blood glucose and serum triglyceride content, a decreased body weight, serum insulin level, and cardiac function-related indexes, accompanied by disrupted myocardial tissue morphology and ultrastructure damage. Using RNA sequencing (RNA-seq), we identified thousands of differentially expressed genes (DEGs) in DbCM with or without SPM treatment. Kyoto Encyclopedia of Genes and Genomes (KEGG) analysis demonstrated that the DEGs were significantly enriched in lipid metabolism and amino acid metabolism pathways. Specifically, quantitative real-time PCR (qRT-PCR) confirmed that SPM protected DbCM by reversing the expressions of lipid metabolism and amino acid metabolism-related genes, including Alox15, Gm13033, pla2g12a, Ptges, Pnpla2, and Acot1. To further reveal the pathogenesis of DbCM, we used proteome-based data-independent acquisition (DIA) and identified 139 differentially expressed proteins (DEPs) with 67 being upregulated and 72 being downregulated in DbCM. Venn intersection analysis showed 37 coexpressed genes and proteins in DbCM, including 29 upregulation and 8 downregulation in DbCM. In the protein-protein interaction (PPI) network constructed by the STRING database, the metabolism-related coexpressed genes and proteins, such as Acot2, Ephx2, Cyp1a1, Comt, Acox1, Hadhb, Hmgcs2, Acot1, Inmt, and Cat, can interact with the identified DEGs and DEPs.

**Conclusion:**

The biomarkers and canonical pathways identified in this study may hold the key to understand the mechanisms of DbCM pathobiology and provide new targets for the therapeutic effect of SPM against DbCM by targeting lipid and amino acid metabolism pathways.

## 1. Background

Diabetic cardiomyopathy (DbCM) is a disease that diabetes mellitus results in direct damage to the structure and function of the myocardium without causing other vascular disease, such as hypertension or coronary artery disease. DbCM is one of the most severe complications of type 1 diabetes (T1D), characterized by combined diastolic and systolic cardiac dysfunction [1, 2]. The pathophysiology of DbCM is complex, and multiple mechanisms contribute to its development and outcome. It has been reported that increased many factors promote the progress of DbCM, including fatty acid (FA) oxidation and lipid toxicity, mitochondrial dysfunction, endoplasmic reticulum stress (ERS), oxidative stress, and increased inflammatory cytokines, but the exact mechanisms have not been well understood [3, 4]. The arrival of high-throughput genomics and proteomics techniques is allowing the expansion of classical studies over large gene and protein datasets; thus, we can better understand the molecular mechanism of DbCM [5, 6].

Spermine (SPM), a small polyamine, is a natural product of cellular metabolism with positively charged alkylamines [7]. SPM regulates cell proliferation, differentiation, and apoptosis and is crucial for diverse physiological processes including immunity, aging, and transcriptional regulation [8, 9]. We have previously found that the intracellular content of SPM was decreased significantly in cardiomyocytes of T1D rats [10, 11]. Numerous studies have shown that SPM plays a protective role in many cardiovascular diseases, such as ischemia-reperfusion injury, myocardial hypertrophy, hypertension, and myocardial infarction [12–15]. Recently, we discovered that SPM reduces hyperglycemia-induced myocardial injury by inhibiting oxidative stress and restoring myocardial energy metabolism [10, 16, 17]. However, the effect of SPM on T1D, especially DbCM, needs to be further explored.

Here, we describe the dynamic changes occurring throughout the nontreated and SPM-treated myocardium of diabetic mice from the morphological, molecular, and omics perspectives. The information from this study may be utilized in identifying novel candidate targets and pathways for SPM treatment of DbCM.

## 2. Materials and Methods

### 2.1. Animal Use and Experimental Design

Male C57BL/6J mice (aged 6-8 weeks and weighted 22-24 g) were supplied by the animal house at second affiliated hospital of Harbin Medical University (Harbin, China). All animal experimental protocols were approved by the Institutional Animal Research Committee of Harbin Medical University and following the ARRIVE Guidelines. Mice were housed in a controlled environment (24 ± 1°C; 60 ± 10% relative humidity; fixed 12/12 h light/dark cycle) with food and water ad libitum and were randomized into three groups after adaptively feeding for 1 week (*n* = 24 per group, no blinding was done in animal experiments): [1] control check group (CK): 0.1 mol/L sterile citrate buffer (pH 4.5) was injected intraperitoneally; [2] diabetic cardiomyopathy model group (DbCM): an intraperitoneal injection of streptozotocin (STZ, 180 mg/kg, dissolved in 0.1 mol/L, pH 4.5 citric acid-citrate sodium buffer) was used to establish type 1 diabetes (T1D) model with normal diet fed for 12 weeks; and [3] spermine group (SPM): an intraperitoneal injection of SPM (1 mg/kg/day) was administered every day for two weeks prior to STZ injection; after that, SPM was injected every other day for 12 weeks. The experimental mice were fasted 12 h prior to the STZ administration; immediately thereafter, 10% sucrose was supplemented in the drinking water for 24 h to avoid sudden hypoglycemia due to insulin hypersecretion. The body weight and blood glucose levels were monitored once weekly after 6 h fasting. Additional details may be found in our previous study [18]. After 12 weeks, the mice were fasted overnight and then anesthetized with intraperitoneal 2% pentobarbital (50 mg/kg, dissolved in normal sodium) for tissue collection. The hearts of the mice were carefully dissected from the surrounding tissues and cut into three parts. One part of tissue samples was fixed with 4% polyformaldehyde, then embedded in paraffin, and dehydrated in gradient sucrose for hematoxylin-eosin (H&E), Masson's trichrome, and Sirius red staining. A second part of tissues was fixed with 2.5% glutaraldehyde for observation with a transmission electron microscope. The third part of tissues was stored at −80°C for further experiments.

### 2.2. Animal Echocardiography

Echocardiography was carried out at 1 day before and 12 weeks after STZ injection using an echocardiographic system with a Vevo1100 ultrasound machine (VisualSonics, Toronto, Canada) at a probe frequency of 10 MHz. Animals were anesthetized under 3% pentobarbital sodium, and standard two-dimensional images were obtained at the papillary muscles which were obtained. The ejection fraction (EF) and fractional shortening (FS) were measured on the M-mode tracings and calculated by the machine as the average of three cardiac cycles [19]. All settings were optimized to obtain maximal signal-to-noise ratio and two-dimensional images to provide optimal endocardial delineation.

### 2.3. Histopathological Examination

Heart tissues were fixed in 4% paraformaldehyde, paraffin-embedded, and sectioned (3 *μ*m). The tissue sections were stained with hematoxylin-eosin (H&E), Masson's trichrome, and Sirius red, respectively. The morphology and extracellular collagen deposition in the cardiac tissue were observed under a microscope.

### 2.4. Ultrastructural Changes Detected by Electron Microscopy

A small piece of heart tissues (1 mm^3^) were fixed in 2.5% (vol/vol) glutaraldehyde for 12 h at 4°C, followed by 1% osmium tetroxide. Then, tissues were dehydrated in ascending gradations of ethanol and embedded in fresh epoxy resin [20]. Ultrathin sections (60–80 nm) were cut and stained with lead citrate before being examined on a Hitachi H-7500 transmission electron microscope.

### 2.5. RNA Sequencing (RNA-Seq) Analysis

Total RNA was isolated from the collected heart tissues with TRIzol Reagent (Invitrogen, Life Technologies Corporation). RNA quality was assessed on an Agilent 2100 Bioanalyzer (Agilent Technologies, CA, USA) and checked using RNase-free agarose gel electrophoresis. Extracted mRNA was enriched by Oligo (dT) beads, with removal of rRNA using a Ribo-Zero™ Magnetic Kit (Epicentre, WI, USA). Then, the enriched mRNA was fragmented into short fragments and reverse transcribed into cDNA with random primers. After synthesis of second-strand cDNA, fragments were then purified, end repaired, polyadenylated, and ligated to Illumina sequencing adapters. Ligation products were size-selected by agarose gel electrophoresis, PCR amplified, and sequenced using Illumina HiSeq 2500 by Gene Denovo Biotechnology Co. (Guangzhou, China) [21]. Clean reads were further filtered by fastp (version 0.18.0) [22]. We used DESeq2 software to identify RNA differential expression between two different groups [23]. The genes/transcripts with the parameter of false discovery rate (FDR) < 0.05 and absolute fold change ≥ 1.5 were considered differentially expressed genes/transcripts.

KEGG enrichment analysis was then used to identify the significantly enriched metabolic or signal transduction pathways in differentially expressed genes (DEGs) comparing with the whole genome background [24]. The calculated *P* value was gone through FDR correction, taking FDR < 0.05 as a threshold. We also performed gene set enrichment analysis (GSEA) using software GSEA and MSigDB to identify the significance of a set of genes in specific pathways between groups [25]. Briefly, we input gene expression matrix and ranked genes by signal-to-noise normalization method. Enrichment scores and *P* value were calculated in default parameters.

### 2.6. Proteome Analysis

Total protein was extracted from myocardial tissue by the cold acetone method. Briefly, samples were transferred into lysis buffer and homogenized for 3 min and 3 times in ice using ultrasonic homogenizer. The homogenate was centrifuged at 15,000 rpm for 15 min at 4°C, and the supernatant was collected. The proteins were digested with sequence-grade modified trypsin (Promega, Madison, WI) at a substrate/enzyme ratio of 50 : 1 (*w*/*w*) at 37°C for 16 h. Proteins were labeled, using the iTRAQ labeling kit (Applied Biosystems) according to manufacturer's instructions with minor modification. Digested protein products were then dried, dissolved, and labeled. The labeled striated and catch samples were fractionated using an SCX column on an HPLC system (LC-20AB, Shimadzu, Japan).

The peptides were analyzed by on-line nanospray LC-MS/MS on an Orbitrap Fusion Lumos coupled to EASY-nLC 1200 system (Thermo Fisher Scientific, MA, USA), using a C18 analytical reverse-phase column (200 nL/min at 40°C). The mass spectrometer was run under data independent acquisition mode and automatically switched between MS and MS/MS mode. Raw data of DIA were processed and analyzed by Spectronaut X (Biognosys AG, Switzerland) with default parameters. These experimental procedures and data analysis were performed by Gene Denovo Biotechnology Co. (Guangzhou, China). After Student's *t*-test, different expressed proteins were filtered if their *Q* value (FDR) was smaller than 0.05 and Absolute AVG log2 ratio was more than 0.58. To annotate the differentially expressed proteins (DEPs), KEGG pathway analysis was also performed to identify the significantly enriched metabolic pathways. The calculated *P* value was gone through FDR correction, taking FDR < 0.05 as a threshold. Protein-protein interaction (PPI) network was identified using String v10 [26]. The network file was visualized using Cytoscape (v3.7.1) software to present a core and hub gene biological interaction.

### 2.7. Quantitative Real-Time PCR (qRT-PCR)

Total RNA was isolated from the heart, and the integrity was detected by agarose gel electrophoresis. Equal amounts of total RNA were used for qRT-PCR analysis with a cDNA kit (Bimake, Houston, TX, USA) according to the manufacturer's instructions. qRT-PCR was performed in triplicate by using SYBR Green qPCR Master Mix (Roche, Basel, Switzerland) and a Roche LightCycler 96 setup and the Real-Time PCR System (Bio-Rad, Hercules, CA, USA). Each well was loaded with a total of 20 *μ*L containing 2 *μ*L of cDNA, 0.4 × 2 *μ*L of target primers, 7.2 *μ*L of water, and 10 *μ*L of SYBR Fast Master Mix. Hot-start PCR was performed for 45 cycles, with each cycle consisting of denaturation for 5 s at 94°C, annealing for 15 s at 58°C, and elongation for 10 s at 72°C. The CFX manager software (version 2.0, Roche Bio-Rad, USA) was used for data analysis. Relative quantification was done using the 2^−*ΔΔ*CT^ method [27]. Expression was normalized against *β*-actin. The sequences of primers were used as follows: arachidonate 15-lipoxygenase (Alox15) (5′-CCCTGTCGGGACTCGGAAGC-3′ and 5′-CCAGTGCCCTCAGGGAGGCT-3′), prostaglandin-endoperoxide synthase 2 pseudogene (Gm13033) (5′-GCACAAATCTGATGTTTGCATTCT-3′ and 5′-CTGGTCCTCGTTCATATCTGCTT-3′), phospholipase A2, group XIIA (Pla2g12a) (5′-TGTTGGGATGTGCACAGAAT-3′ and 5′-GCAGATAGCGCACAGATTCA-3′), prostaglandin E synthase (Ptges) (5′-CATGTGAGTCCCTGTGATGG-3′ and 5′-GACTGCAGCAAAGACATCCA-3′), patatin-like phospholipase domain containing 2 (Pnpla2) (5′-AGCTCATCCAGGCCAATGTCT-3′ and 5′-TGTCTGAAATGCCACCATCCA-3′), acyl-CoA thioesterase 1 (Acot1) (5′-GCAGCCACCCCGAGGTAAA-3′ and 5′-GCCACGGAGCCATTGATG-3′), and *β*-actin (5′-ATGGATGACGATATCGCTGC-3′ and 5′-CTTCTGACCCATACCCACCA-3′).

### 2.8. Western Blotting

Heart tissue samples were homogenized and lysed in RIPA buffer. Total protein from heart tissue was extracted by using Minute™ Total Protein Extraction Kit (Invent Biotechnologies, Plymouth, MN, USA). After quantification and denaturation, equal amounts of proteins were separated by SDS-PAGE and transferred using electrophoresis to a PVDF membrane (Millipore, Schwalbach, Germany). The membranes were blocked for 1 h and incubated overnight at 4°C with specific primary antibody. Then, the membrane was incubated in secondary antibody for 2 h at room temperature on a shaker. The signals were detected by the Enhanced Chemiluminescent (ECL) kit (HaiGene, Harbin, China) and the Multiplex Fluorescent Imaging System (ProteinSimple, California, USA). The intensities of protein bands were quantified by a Bio-Rad ChemiDoc™ EQ densitometer and Bio-Rad Quantity One software (Bio-Rad Laboratories, Hercules, CA, USA) and normalized to *β*-tubulin.

### 2.9. Statistical Analysis

Statistical analyses were performed using SPSS 21.0 software. GraphPad Prism was used for mapping and curve fitting. All data are presented as the means ± standard errors of the means (SEM). One-way analysis of variance (ANOVA) followed by the Student's *t*-test was applied to analyze statistically significant differences between two groups. For all experiments, analyses were done at least in biological triplicate. A *P* < 0.05 was considered statistically significant. For the myocardial tissue used for RNA-seq and proteome analysis, 18 mouse hearts were randomly divided into 3 parts, that is, 6 hearts each for the mixed samples.

## 3. Results

### 3.1. SPM Improves Heart Functions and Biochemical Parameters in DbCM Mice

**Figure 1:**
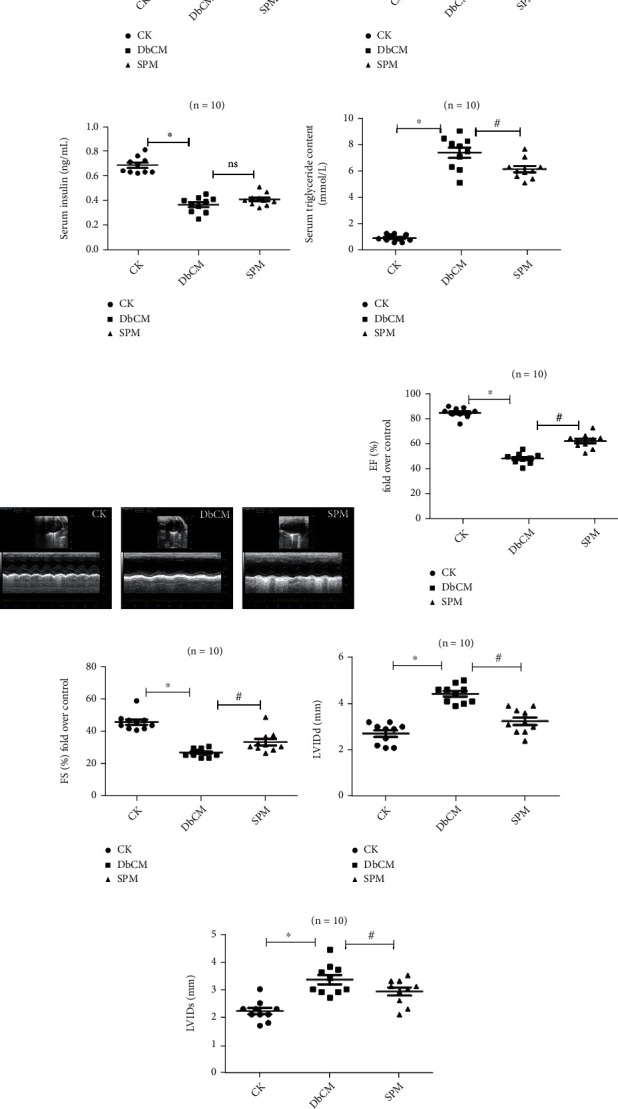
SPM alleviates the changes in heart function and biochemical parameters in DbCM mice. (a) Blood glucose concentration. (b) Body weight. (c) Serum insulin concentration. (d) Serum triglyceride content. (e) Cardiac function ultrasonogram. (f) Ejection fraction (EF%). (g) Fractional shortening (FS%). (h) Left ventricular internal dimension at end-diastole (LVIDd). (i) Left ventricular internal dimension at end-systole (LVIDs). Data are represented as the mean ± SEM for *n* = 10 per group. ^∗^*P* < 0.05 versus the CK group. ^#^*P* < 0.05 versus the DbCM group.

A single intraperitoneal injection of STZ in mice resulted in metabolic phenotypes that are characteristics of human T1D, including increased blood glucose and serum triglyceride content, decreased serum insulin, and lower body weight (Figures 1(a)–1(d) and Table S1). STZ-injected mice also developed common signs of T1D, including polydipsia, polyuria, and noticeable hypoactivity and weakness (data not shown). We next examined the cardiac functions of T1D mice. Echocardiography revealed significantly impaired cardiac function 12 weeks after STZ delivery, as shown by decreased left ventricular ejection fraction (EF%) and fractional shortening (FS%), and increased left ventricular internal dimension (LVID) at end-diastole (LVIDd) and end-systole (LVIDs) (Figures 1(e)–1(i) and Table S2). Taken together, these results showed that a DbCM model was successfully established in STZ-induced T1D mice. To explore the effect of SPM on DbCM, we administered SPM to the mice before and after STZ delivery and observed that SPM attenuated the changes of cardiac function-related indexes and decreased the content of serum triglyceride (Figures 1(d)–1(i)).

**Figure 2:**
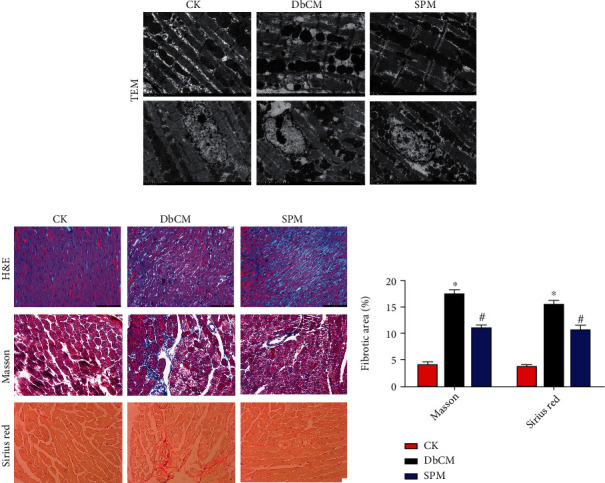
SPM attenuates the myocardial ultrastructural and histomorphological changes of DbCM mice. (a) Myocardial ultrastructure by electron microscopy (original magnifications ×2,000 or 5,000; scale bars = 2 *μ*m or 5 *μ*m). (b) Myocardial histomorphology by H&E staining (original magnification ×200; scale bars = 200 *μ*m), Masson's trichrome (collagen deposition displayed in blue), and Sirius red staining (collagen fibers displayed in red; original magnification ×400; scale bars = 100 *μ*m). (c) Fibrotic area analysis. ^∗^*P* < 0.05 versus the CK group. ^#^*P* < 0.05 versus the DbCM group.

We further examined the ultrastructure of cardiomyocytes by transmission electron microscopy (TEM). Mice receiving STZ injection developed dysplasia of sarcomere, rupture of myofilaments, disappearance of nuclei, and abnormal mitochondrial structure, all of which were minimally visible in SPM-injected DbCM mice (Figure 2(a)). In DbCM mice, cardiomyocytes were disorderly arranged with dissolved nuclei, coagulative necrosis, and wavy myocardial fibers. Cardiomyocyte hypertrophy and nuclear malformation were also visible in the DbCM group (Figure 2(b), H&E staining). Masson and Sirius red staining revealed a large amount of collagen deposition in the myocardial tissue interstitial and perivascular areas in DbCM mice (Figures 2(b) and 2(c), Masson and Sirius red staining). However, the myocardium histological changes of SPM-injected mice were alleviated compared to the DbCM group.

### 3.2. Transcriptome Profiles for the Myocardium of CK, DbCM, and SPM Mice

**Figure 3:**
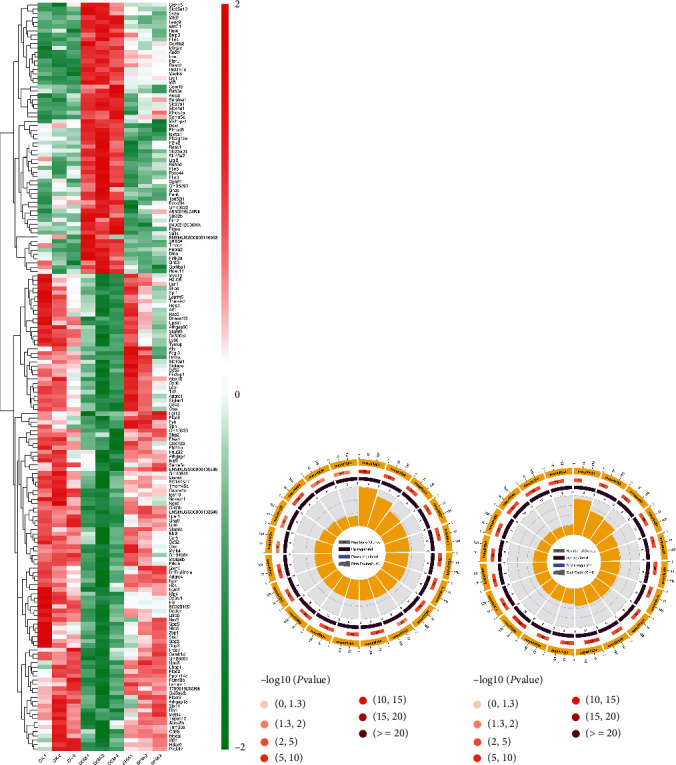
Transcriptome profiles for the myocardium of CK, DbCM, and SPM mice. (a) The heat map showing the 174 overlapped genes producing differentially expressed in DbCM and reversed expression by SPM. Red color shows the genes with significantly elevated transcription levels, and green color shows the genes with significantly reduced transcription levels. (b) KEGG pathway analysis of the DEGs belonging to metabolic related pathways of DbCM/CK. (c) KEGG pathway analysis of the DEGs belonging to metabolic related pathways of SPM/DbCM. Numbers and *P* values of DEGs in each pathway.

RNA-seq analysis was performed to evaluate differentially expressed genes (DEGs) in the myocardium among the control check group (CK), diabetic cardiomyopathy model group (DbCM), and spermine group (SPM) mice. The biological repeatability of the samples in each group was good, and the correlation heat map analysis between samples is shown in Figure S1. Transcriptomic data have showed that 1318 genes were differentially expressed in DbCM when comparing to CK and 1393 genes were differentially expressed when comparing SPM to DbCM. Then, we cross-matched these differentially expressed candidates from the DbCM/CK and SPM/DbCM groups and further identified 174 overlapped RNAs [28]. In other words, SPM may play a role in DbCM by regulating the expression of these 174 candidates. Subsequently, we plotted a heat map for the 174 DEGs with red plots representing elevated transcription levels and the green plots indicating downregulation of transcription levels (Figure 3(a) and Table S3).

The DEGs in the three groups were further subjected to KEGG pathway analysis. Most DEGs between DbCM and CK were enriched in the PI3K-Akt signaling pathway, metabolic pathways, ECM receptor interaction, AGE-RAGE signaling pathway in diabetic complications, cell adhesion molecules (CAMs), Ras signaling pathway, HIF-1 signaling pathway, p53 signaling pathway, MAPK signaling pathway, and PPAR signaling pathway (Figure S2A and Table S4). The DEGs between SPM and DbCM were enriched in the metabolic pathways, complement and coagulation cascades, oxidative phosphorylation, PPAR signaling pathway, chemokine signaling pathway, MAPK signaling pathway, and PI3K-Akt signaling pathway, insulin resistance, calcium signaling pathway, Wnt signaling pathway, etc. (Figure S2B and Table S5). In particular, the DEGs identified in DbCM/CK and SPM/DbCM were commonly enriched in the metabolic pathways. Thirty metabolic pathways were identified in DbCM/CK, including arachidonic acid metabolism, carbon metabolism, glycerophospholipid metabolism, metabolism of xenobiotics by cytochrome P450, glutathione metabolism, valine, leucine, and isoleucine degradation, fatty acid metabolism, and biosynthesis of unsaturated fatty acids, while 32 metabolic pathways were identified in SPM/DbCM, including biosynthesis of amino acids, carbon metabolism, valine, leucine, and isoleucine degradation, glycerophospholipid metabolism, glutathione metabolism, fatty acid metabolism, linoleic acid metabolism, and biosynthesis of unsaturated fatty acids (Figures 3(b) and 3(c)). The annotation of KEGG pathways suggest that the occurrence of DbCM and the therapeutic effect of SPM may be closely related to the metabolic pathways such as lipid metabolism and amino acid metabolism.

### 3.3. SPM Reduces DbCM Myocardial Damage by Regulating Lipid Metabolism and Amino Acid Metabolism Pathways

**Figure 4:**
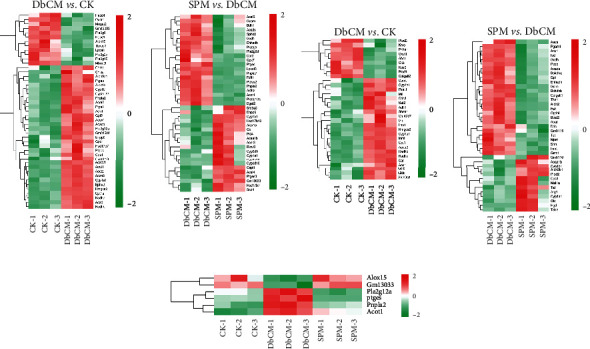
SPM reduces DbCM myocardial damage by regulating lipid metabolism and amino acid metabolism pathways. (a) The heat map showed the DEGs related to lipid metabolism pathway in DbCM/CK and SPM/DbCM. (b) The heat map showed the DEGs related to amino acid metabolism pathway in DbCM/CK and SPM/DbCM. (c) The heat map showed the coexpressed genes related to metabolic pathway in the CK, DbCM, and SPM groups. Red color shows the genes with significantly elevated transcription levels, and green color shows the genes with significantly reduced transcription levels.

To determine whether the therapeutic effect of SPM on DbCM is related to its regulation of lipid metabolism and amino acid metabolism pathways, we further analyzed the DEGs enriched in these two pathways. First, we screened out the DEGs involved in the lipid metabolism pathway (Figure 4(a)) and amino acid metabolism pathway (Figure 4(b)) between the DbCM compared to the CK group and the SPM compared to the DbCM group, respectively, and then plotted a heat map. The red plots represented upregulation of genes, and the green plots indicated downregulation of genes (Figures 4(a) and 4(b) and Tables S6 and S7). Gene set enrichment analysis (GSEA) results are also consistent with the above results. Compared with CK, 5 of the top 10 gene sets in DbCM mice were related to amino acid metabolism or lipid metabolism (Figure S3A); in addition, 5 of the top 10 gene sets in SPM mice were also related to metabolism (Figure S3B).

Among these DEGs, we noticed that Alox15, Gm13033, pla2g12a, Ptges, Pnpla2, and Acot1 were significantly differentially expressed in the DbCM group, which could be reversed by SPM. In other words, Alox15 and Gm13033 were downregulated in the DbCM group and upregulated by SPM, while pla2g12a, Ptges, Pnpla2, and Acot1 were upregulated in the DbCM group and downregulated by SPM (Figure 4(c)).

**Figure 5:**
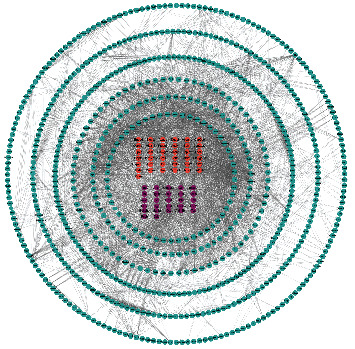
Gene interaction network analysis on metabolic pathway-related candidates between SPM and DbCM.

Furthermore, we performed a gene interaction (GI) network analysis on the two metabolic pathway-related candidates between the SPM group and the DbCM group. The results confirmed that the candidates involved two metabolic pathways, including Alox15, Gm13033, pla2g12a, Ptges, Pnpla2, and Acot1, were at the core reflecting that SPM could protect DbCM by regulating lipid and amino acid metabolisms (Figure 5).

### 3.4. Identification of Proteome Changes in DbCM

**Figure 6:**
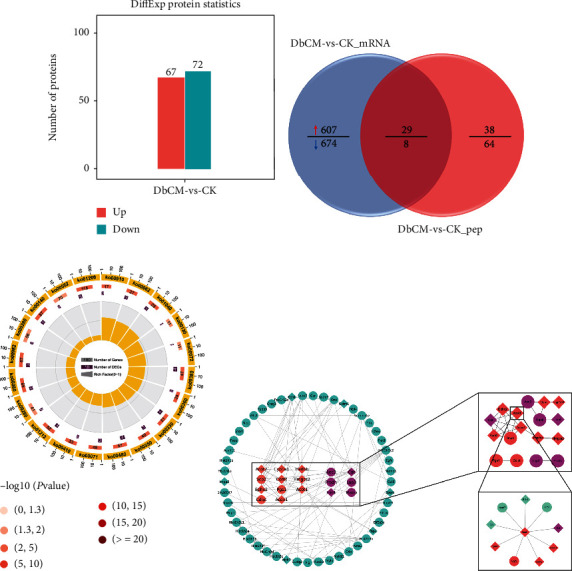
Identification of proteome changes in DbCM. (a) The amounts of differentially expressed proteins by comparing DbCM to CK. (b) KEGG pathway analysis of the DEPs belonging to metabolic related pathways of DbCM/CK. Numbers and *P* values of DEPs in each pathway. (c) Venn diagrams showing the 37 overlapped candidates that differentially expressed on both RNA level and protein level. (d) Protein-protein interaction network analysis on metabolic pathway-related candidates between DbCM and CK.

We further measured the proteome in pursuit of causative pathways. A total of 139 proteins were found to be expressed differently between the CK and DbCM groups, in which 67 were increased and 72 were decreased in the DbCM (Figure 6(a)). The protein expression level changes between CK and DbCM mice were then determined by the KEGG analysis of differentially expressed proteins (DEPs). KEGG results showed that DEPs are mainly enriched in peroxisome, metabolic pathways, PPAR signaling pathway, tryptophan metabolism, arginine and proline metabolism, glutathione metabolism, biosynthesis of unsaturated fatty acids, complement and coagulation cascades, HIF-1 signaling pathway, FoxO signaling pathway, and other pathways (Figure S4 and Table S8). Interestingly, we again noticed that many DEPs between the two groups can be enriched in lipid metabolism and amino acid metabolism pathways, including arginine and proline metabolism, glutathione metabolism, tryptophan metabolism, metabolism of xenobiotics by cytochrome P450, fatty acid degradation and elongation, biosynthesis of unsaturated fatty acids, glycerophospholipid metabolism, and citrate cycle (TCA cycle) (Figure 6(b) and Table S9).

By comparing the DEGs and DEPs between the DbCM and CK groups for Venn analysis, we found that 29 were upregulated and 8 were downregulated among the coexpressed genes or proteins (Figure 6(c)). This also suggests that these 37 common DEGs or DEPs are the most closely related to the progress of DbCM and may become effective therapeutic targets.

Finally, we performed protein-protein interaction (PPI) network analysis on DEGs and DEPs that involved in lipid metabolism and amino acid metabolism pathways between the CK and DbCM groups. In the PPI network constructed by the STRING database, Acot2, Ephx2, Cyp1a1, Comt, Acox1, Hadhb, Hmgcs2, Acot1, Inmt, and Cat could interact with other DEGs and DEPs involved in the KEGG pathway, especially Acot1 (Figure 6(d)), indicating that these genes or proteins may participate in the pathogenesis of DbCM through interaction with the others.

### 3.5. SPM Regulates the Expressions of Genes Related with Metabolic Pathways

**Figure 7:**
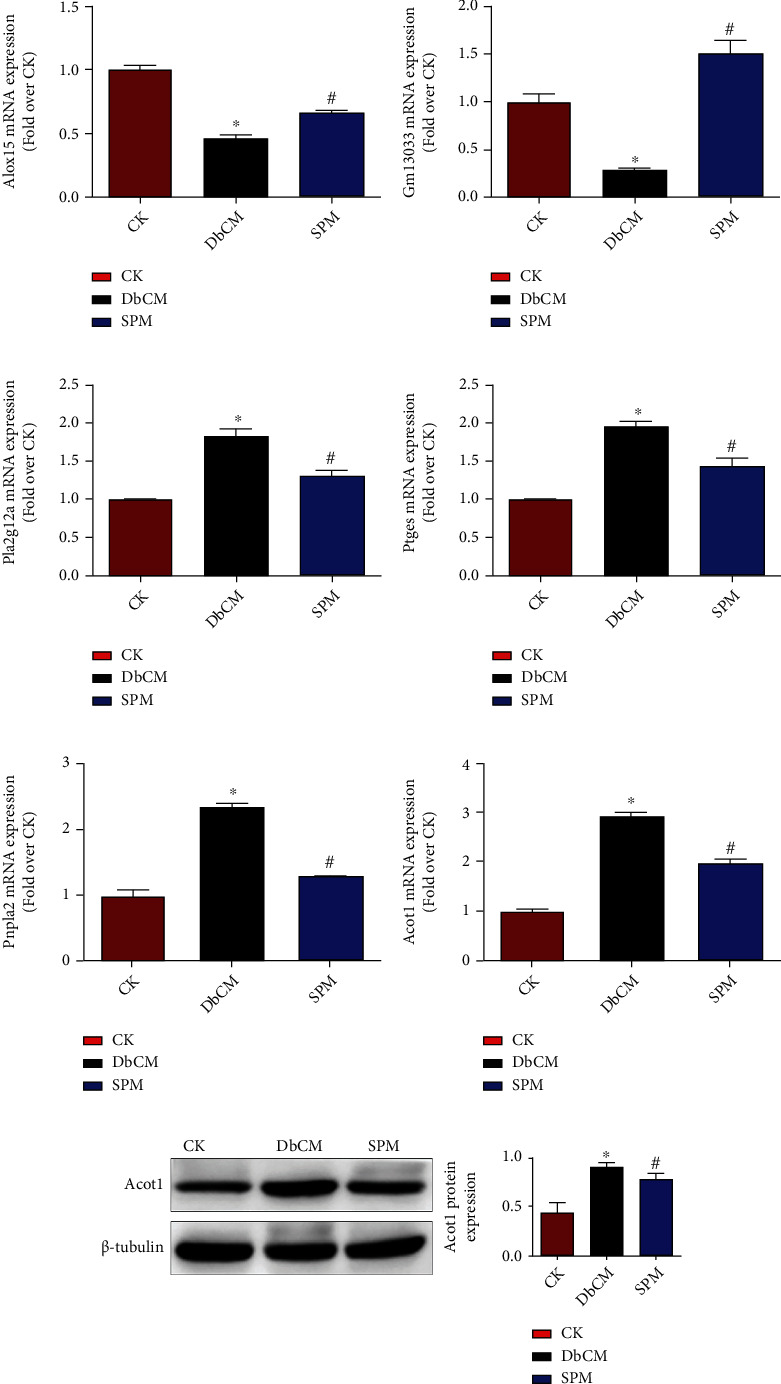
SPM regulates the expressions of genes related with metabolic pathways. The mRNA expression levels of genes related with metabolic pathways, including (a) Alox15, (b) Gm13033, (c) pla2g12a, (d) Ptges, (e) Pnpla2, and (f) Acot1. (g) The protein expression levels of Acot1. Data are represented as the mean ± SEM for *n* = 4 per group. ^∗^*P* < 0.05 versus CK group. ^#^*P* < 0.05 versus the DbCM group.

In order to validate the molecular characteristics of DbCM and the therapeutic effect of SPM at the transcriptomics level, we used qRT-PCR to confirm the mRNA expression of metabolic pathway-related candidates, including Alox15, Gm13033, pla2g12a, Ptges, Pnpla2, and Acot1. The results were consistent with the results of RNA-seq analysis. In comparison with the control, the expression levels of Alox15 and Gm13033 were downregulated while the expression levels of pla2g12a, Ptges, Pnpla2, and Acot1 were upregulated in the DbCM group. In addition, the above changes were reversed by SPM treatment (Figures 7(a)–7(f)). Western blotting also confirmed Acot1 was increased in DbCM but reversed by SPM (Figure 7(g)). These results were consistent with the proteomics and transcriptomics data.

## 4. Discussion

DbCM is a common and severe complication of T1D with an increased incidence rate globally [1, 2, 29]. DbCM is manifested with diastolic function impairments and heart failure at the early stage, which contributes to the early morbidity and high mortality of T1D patients [30, 31]. Multifactorial pathogenesis was observed in the progress of DbCM, such as metabolic disorders, autonomic dysfunction, abnormal homeostasis, interstitial fibrosis, and structural protein alteration [1, 32]. Even with these evidences, the pathogenesis of DbCM still remains controversial. Thus, elaborating the molecular mechanism of DbCM and exploring therapeutic targets remains an urgent public health need [33, 34].

In this study, we utilized a well-established T1D mouse model by intraperitoneal injection of STZ and confirmed that STZ leads to hyperglycemia, hyperlipidemia, hyperinsulinemia and weight loss, and abnormal cardiac function and structure. SPM is an important polyamine metabolite with positively charged alkyl amines and is involved in many cellular processes [7]. We previously demonstrated that the metabolic imbalance of polyamines is related to a variety of cardiovascular diseases, including diabetes, myocardial ischemia-reperfusion injury, and myocardial hypertrophy [10, 12, 13, 35]. Our recent data have shown the critical roles of SPM in protecting DbCM in rats, while there are still many details awaiting further research, including its effect on mouse myocardial injury [10, 16, 17]. Here, we determined that SPM has a protective effect on the DbCM model of C57BL/6J mice, which is reflected in its improvement of the general state and maintenance of cardiac function and structure. The experimental data suggested that SPM might be a potential modulator for myocardial injury in DbCM.

To explore the features of DbCM mice with SPM treatment, the characteristics and differences of each group were comprehensively analyzed using RNA-seq [36]. In previous studies, we found that thousands of significantly different genes were identified in DbCM/CK and SPM/DbCM. In addition, 174 genes were found in an analysis of differential expression in the DbCM but reversed by SPM [28]. Subsequently, we further analyzed the biological significance of these DEGs by enrichment analysis of KEGG, which revealed many key pathways related to the mice during hyperglycemia and SPM treatment. Compared with other key pathways, the lipid metabolism and amino acid metabolism pathways have higher enrichment, with 45 and 32 DEGs that were enriched in the DbCM/CK group and 41 and 39 DEGs were enriched in the SPM/DbCM group, respectively. More interesting, we noticed that Alox15 (arachidonate 15-lipoxygenase), Gm13033 (prostaglandin-endoperoxide synthase 2 pseudogene), pla2g12a (phospholipase A2-group XIIA), Ptges (prostaglandin E synthase), Pnpla2 (patatin-like phospholipase domain containing 2), and Acot1 (acyl-CoA thioesterase 1) were significantly downregulated or up-regulated in the DbCM group. The expressions of these genes could be reversed by SPM. Based on the interaction network, we found that abovementioned molecules were in the center of the interaction network and involved in the regulation of extensive genes related to others. Collectively, this discovery strongly suggests that DbCM progress may be related to lipid metabolism and amino acid metabolism disorders, and SPM protects against DbCM by adjusting the expression of these genes related to metabolic pathways.

We further used proteomics-based data-independent acquisition to deeply analyze the possible biomarkers and therapeutic targets of DbCM. Similar to the RNA-seq results, the DIA data provided evidence of high proteome differences between DbCM and CK, which are reflected in 139 proteins differentially expressed. Although differential expression may be associated with the processes of protein synthesis, posttranslational modification, and protein degradation, all may result in variation in protein abundance and have functional implications [37]. Enrichment analysis of KEGG showed that many DEPs participate in lipid metabolism and amino acid metabolism pathways. That is to say, changes in metabolic pathways affect the progression of DbCM on both RNA and protein levels.

We integrate RNA-seq with a DIA method and revealed an attractive phenomenon by analyzing the data association between the transcriptome and the proteome. A total of 37 candidates are coexpressed in the two omics. The cross-identified proteins can not only provide clues about DbCM pathogenesis but can also serve as biomarkers being the diagnostic or prognostic potential [38, 39]. Among the 37 coexpressed proteins, PPI network showed that Acot2, Ephx2, Cyp1a1, Comt, Acox1, Hadhb, Hmgcs2, and Acot1 involved in lipid metabolism pathways and Inmt and Cat involved in amino acid metabolism pathways, all of which can interact with other proteins to contribute to the development of DbCM. It is worth noting that among the above candidates, Acot1 has been reported to be important for controlling the rate of fatty acid (FA) oxidation, protecting cardiotoxicity through antiferroptosis, and regulating intracellular signal transduction [40, 41] that can play a major regulatory role by interacting with multiple factors.

Through qRT-PCR and western blotting, we verified some important factors screened in omics. We found that SPM affects the progress of DbCM by regulating factors related to metabolic pathways. Interestingly, we found an inconsistency between RNA and protein expressions. Translational efficiency, mRNA, and protein turnover rates are likely to have an impact on these inconsistencies [42, 43].

The development of DbCM may have a very complicated regulatory network, and this study provides only a preliminary exploration. However, our comprehensive study has revealed the differences between DbCM and SPM treatments at the morphological, molecular, and omics levels, which lay a foundation for SPM to improve DbCM through gene or protein regulation.

## 5. Conclusion

In summary, we investigated the changes occurring throughout the nontreated and SPM-treated myocardium of diabetic mice at the morphological, molecular, and omics levels. Our research integrates RNA-seq with a DIA method, to capture the molecular mechanism of DbCM and the potential therapeutic target of SPM. That is, SPM has a protective effect on DbCM, which may be related to the improvement of lipid metabolism and amino acid metabolic pathway disorders by regulating Acot1 and other targets.

## Data Availability

Sequencing data reported here are available at GEO, under the superseries, GSE161052 (https://www.ncbi.nlm.nih.gov/geo/query/acc.cgi?acc=GSE161052).

## Abbreviations

Acot1: Acyl-CoA thioesterase 1
Alox15: Arachidonate 15-lipoxygenase
DbCM: Diabetic cardiomyopathy
DEGs: Differentially expressed genes
DIA: Data-independent acquisition
DEPs: Differentially expressed proteins
EF: Ejection fraction
ERS: Endoplasmic reticulum stress
FA: Fatty acid
FDR: False discovery rate
FS: Fractional shortening
GSEA: Gene set enrichment analysis
GI: Gene interaction
Gm13033: Prostaglandin-endoperoxide synthase 2 pseudogene
HE: Hematoxylin-eosin
KEGG: Kyoto Encyclopedia of Genes and Genomes
LVIDd: Left ventricular internal dimension at end-diastole
LVIDs: Left ventricular internal dimension at end-systole
Pla2g12a: Phospholipase A2-group XIIA
Pnpla2: Patatin-like phospholipase domain containing 2
PPI: Protein-protein interaction
Ptges: Prostaglandin E synthase
qRT-PCR: Quantitative real-time PCR
RNA-seq: RNA sequencing
SPM: Spermine
STZ: Streptozotocin
T1D: Type 1 diabetes.

## Supplementary Materials

Supplementary Materials Figure S1: correlation analysis of myocardial tissue samples in the CK, DbCM, and SPM groups. Figure S2: KEGG pathway analysis of the DEGs in DbCM/CK and SPM/DbCM. (A) Enrichment pathway of DEGs in DbCM compared to CK. (B) Enrichment pathway of DEGs in SPM compared to DbCM. Figure S3: GSEA plot showing most enriched gene sets of all detected genes in DbCM and SPM mice. (A) The top five significant enriched gene sets related to metabolic pathway in the DbCM group. (B) The top five significant enriched gene sets related to metabolic pathway in the SPM group. Figure S4: KEGG analysis of the DEPs in DbCM/CK. Table S1: biochemical parameters in CK, DbCM, and SPM mouse. Table S2: heart function-related indexes in CK, DbCM, and SPM mouse. Table S3: Venn analysis identified 174 genes that differentially expressed in DbCM but reversally regulated by SPM. Table S4: KEGG analysis of the DEGs in DbCM compared to CK. Table S5: KEGG analysis of the DEGs in SPM compared to DbCM. Table S6: the DEGs in the metabolic related pathways of DbCM/CK. Table S7: the DEGs in the metabolic related pathways of SPM/DbCM. Table S8: KEGG analysis of the DEPs in DbCM compared to CK. Table S9: the DEPs in the metabolic related pathways of DbCM/CK.
