# Supplementary material for: Transcriptomics Coupled to Proteomics Reveals Novel Targets for the Protective Role of Spermine in Diabetic Cardiomyopathy
Source: Oxid Med Cell Longev. 2022 Apr 9;2022:5909378. doi: 10.1155/2022/5909378 (PMC9013312; doi:10.1155/2022/5909378)
Supplement: Supplementary Materials — Figure S1: correlation analysis of myocardial tissue samples in the CK, DbCM, and SPM groups. Figure S2: KEGG pathway analysis of the DEGs in DbCM/CK and SPM/DbCM. (A) Enrichment pathway of DEGs in DbCM compared to CK. (B) Enrichment pathway of DEGs in SPM compared to DbCM. Figure S3: GSEA plot showing most enriched gene sets of all detected genes in DbCM and SPM mice. (A) The top five significant enriched gene sets related to metabolic pathway in the DbCM group. (B) The top five significant enriched gene sets related to metabolic pathway in the SPM group. Figure S4: KEGG analysis of the DEPs in DbCM/CK. Table S1: biochemical parameters in CK, DbCM, and SPM mouse. Table S2: heart function-related indexes in CK, DbCM, and SPM mouse. Table S3: Venn analysis identified 174 genes that differentially expressed in DbCM but reversally regulated by SPM. Table S4: KEGG analysis of the DEGs in DbCM compared to CK. Table S5: KEGG analysis of the DEGs in SPM compared to DbCM. Table S6: the DEGs in the metabolic related pathways of DbCM/CK. Table S7: the DEGs in the metabolic related pathways of SPM/DbCM. Table S8: KEGG analysis of the DEPs in DbCM compared to CK. Table S9: the DEPs in the metabolic related pathways of DbCM/CK. [file 5909378.f1.zip › supplementary table.pdf]

Supplemental file

Table SI. Biochemical parameters in CK, DbCM and SPM mouse (mean ± SEM, n=10)

Group	Blood Glucose Concentration (mmol/L)	Body Weight (g)	Serum Insulin (ng/mL)	Serum Triglyceride Content (mmol/L)
CK	5.5±1.1	22.4±1.0	0.7±0.1	1.0±0.2
DbCM	26.1±3.1*	16.2±2.2*	0.4±0.1*	7.5±1.2*
SPM	24.6±5.8	17.7±1.6	0.4±0.1	6.2±0.7#

*P < 0.05 versus CK group; #P < 0.05 versus DbCM group.

CK, control check; DbCM, diabetic cardiomyopathy; SPM, spermine.

Table SII. Heart function related indexes in CK, DbCM and SPM mouse (mean ± SEM, n=10)

Group	EF (%)	FS (%)	LVIDd (mm)	LVIDs (mm)
CK-0W	78.2±4.5	48.1±3.2	2.4±0.4	2.1±0.3
DbCM-0W	76.4±4.8	47.6±6.7	2.4±0.3	2.1±0.5
SPM-0W	77.7±4.7	49.4±5.4	2.4±0.5	2.0±0.5
CK-12W	75.0±4.0	46.0±5.1	2.7±0.4	2.2±0.3
DbCM-12W	48.6±4.1*	27.2±2.5*	4.4±0.4*	3.3±0.5*
SPM-12W	68.1±5.6#	33.7±6.3#	3.2±0.5#	2.9±0.4#

*P < 0.05 versus CK-12W group; #P < 0.05 versus DbCM-12W group.

CK, control check; DbCM, diabetic cardiomyopathy; SPM, spermine; EF, ejection fraction; FS, fractional shortening; LVIDd, left ventricular internal dimension at end-diastole; LVIDs, left ventricular internal dimension at end-systole.

Table SIII. Venn analysis identified 174 genes that differentially expressed in DbCM but reversal regulated by SPM.

Gene_ID	CK-1	CK-2	CK-3	DbCM-1	DbCM-2	DbCM-3	SPM-1	SPM-2	SPM-3	KEGG_ID	symbol	ncbi_description
ENSMUSG00000000682	22.77271731	15.51138441	12.94373218	8.425011863	2.737834646	6.902498623	15.41560521	12.16070637	10.69426228	K06488	Cd52	CD52 antigen
ENSMUSG00000002111	10.2434683	6.783694526	5.785771471	4.641790254	4.31206922	4.009051857	8.379184085	6.595951844	5.295617353	K09438	Spi1	spleen focus forming virus (SFFV) proviral integration oncogene
ENSMUSG00000002325	14.10689242	11.95358574	11.42358155	7.405403474	6.276348554	6.921892999	9.049737664	8.751628539	8.959708244	K04693	Irf9	interferon regulatory factor 9
ENSMUSG00000003206	1.5359603	1.019761406	1.303695798	0.501796029	0.056718673	0.297524819	1.425065103	0.925426546	0.715872017	None	Ebi3	Epstein-Barr virus induced gene 3
ENSMUSG00000003283	3.373111559	3.17827263	2.285303675	1.468261272	1.016842184	2.201972842	3.172993036	2.897773747	2.733875116	K08893	Hck	hemopoietic cell kinase
ENSMUSG00000004698	2.914711146	3.690483135	3.966431525	2.28623019	2.126821674	2.5260694	3.185867321	3.200345485	3.369453258	K11409	Hdac9	histone deacetylase 9
ENSMUSG00000004730	11.14434453	10.99392867	9.106705695	5.45840129	4.54817899	5.354803545	15.61509346	11.27192598	6.957244046	K04591	Adgre1	adhesion G protein-coupled receptor E1
ENSMUSG00000008496	0.278105477	0.447832641	0.164924511	0.216462479	0.05535167	0.064523118	0.282358085	0.30989494	0.232872814	K09364	Pou2f2	POU domain, class 2, transcription factor 2
ENSMUSG00000014503	5.965719592	8.404039797	8.327572486	5.074289674	4.960198954	5.231770233	6.548698198	6.662354446	6.984792332	K04991	Pkd2l2	polycystic kidney disease 2-like 2
ENSMUSG00000015340	5.890592169	6.345879518	4.960006503	3.957759163	2.644579766	3.522306925	7.154212855	6.048024013	5.095025787	K08008	Cybb	cytochrome b-245, beta polypeptide
ENSMUSG00000015355	5.103919495	5.333326357	4.002885948	3.226740886	2.373097821	2.098854468	6.075893855	5.058450715	3.884643421	K06479	Cd48	CD48 antigen

ENSMUSG00000015968	0.647536622	0.816379296	0.733868456	0.359746915	0.355798388	0.388089468	0.575842305	0.507348338	0.572439896	K04851	Cacna1d	calcium channel, voltage-dependent, L type, alpha 1D subunit
ENSMUSG00000016984	0.703229595	0.759228814	0.4313878	0.366977608	0.320619751	0.252961265	0.550139828	0.645463716	0.569421221	None	Etaa1	Ewing tumor-associated antigen 1
ENSMUSG00000017950	0.053668852	0.040467836	0.045987008	0	0	0	0.349592687	0.180021009	0	K07292	Hnf4a	hepatic nuclear factor 4, alpha
ENSMUSG00000018654	1.710061508	1.475849349	1.061967641	1.072817252	0.494418094	0.581882018	1.221963278	1.107230671	1.193898625	K09220	Ikzf1	IKAROS family zinc finger 1
ENSMUSG00000018924	0.138826741	0.476290804	0.069390939	0.020902508	0.01358517	0.02375423	0.384771342	0.169502223	0.145085407	K00460	Alox15	arachidonate 15-lipoxygenase
ENSMUSG00000020143	2.22816595	2.015202242	1.877940335	1.092093237	0.944676986	1.23662283	1.749519634	1.676118344	1.457554847	K12367	Dock2	dedicator of cyto-kinesis 2
ENSMUSG00000020181	1.631547273	1.520915482	1.386031419	0.952796177	0.818002608	1.08565797	1.044505019	1.292725049	1.537334409	None	Nav3	neuron navigator 3
ENSMUSG00000020437	1.886115533	1.275367006	1.29452652	1.133815878	0.743788059	0.842945247	1.736642698	1.394680947	1.136685543	K10356	Myo1g	myosin IG
ENSMUSG00000020620	2.383340765	2.905152288	2.639293671	1.733191828	1.626854766	2.29342883	2.513523522	2.708129064	2.662433512	K05650	Abca8b	ATP-binding cassette, sub-family A (ABC1), member 8b
ENSMUSG00000021135	0.095607095	0.370064359	0.191152398	0.057580492	0	0.016359045	1.299276572	0.75427259	0.05450035	K14341	Slc10a1	solute carrier family 10 (sodium/bile acid cotransporter family), member 1
ENSMUSG00000021219	3.740533509	4.754181326	3.446161663	2.550147067	2.401205545	2.611328695	3.659970028	3.293897369	2.990867639	K16449	Rgs6	regulator of G-protein signaling 6
ENSMUSG00000021423	4.523077647	3.935105009	2.927216952	1.941748718	1.085198972	1.57770923	3.319797375	2.434163064	1.91778691	None	Ly86	lymphocyte antigen 86
ENSMUSG00000021998	15.97723751	15.03558678	10.497178	8.717912925	6.449906971	8.194003413	17.1571484	14.0490739	10.62156628	K17276	Lcp1	lymphocyte cytosolic protein 1
ENSMUSG00	3.34433	2.06098	2.37782	1.23865	1.20756	1.300544	2.7022	2.11640	1.56319	None	Epsti1	epithelial stromal interaction

000022014	6199	1427	3393	8006	179	095	33805	0148	8278			1 (breast)
ENSMUSG00	0.95230	1.02512	0.77662	0.49052	0.40463	0.503845	1.9268	1.75350	1.01190			
000022148	2552	9577	7342	3663	8539	109	93438	3105	1405	K17698	Fyb	FYN binding protein
ENSMUSG00	5.61409	5.40429	3.97145	2.56208	2.38632	2.722493	4.1927	3.78154	3.36704			
000022488	0014	6251	5288	969	0023	148	72401	0361	5168	K05750	Nckap1l	NCK associated protein 1 like
ENSMUSG00	1.31768	0.97427	1.12330	0.33472	0.71196	0.753856	1.2142	1.57164	2.25034			
000022523	6247	0237	797	0539	5365	597	31752	2156	9361	K04358	Fgf12	fibroblast growth factor 12
ENSMUSG00	1.96871	2.02596	1.51009	1.10924	0.76333	1.207613	1.5498	1.67458	1.50575		Tmem45	
000022754	3275	8782	687	5231	9689	695	20054	3288	125	None	a	transmembrane protein 45a
ENSMUSG00	13.7522	11.9227	10.1657	9.31651	6.96415	7.209762	12.799	10.4232	9.88200			hematopoietic cell specific
000022831	555	3573	5193	5114	8435	306	64995	8073	024	K06106	Hcls1	Lyn substrate 1
ENSMUSG00	3.25444	3.24966	2.81392	2.17341	1.55382	2.189261	2.5635	2.82856	2.69300			StAR-related lipid transfer
000024378	5272	1688	3066	7883	6811	985	66738	9777	3763	None	Stard4	(START) domain containing 4
ENSMUSG00	6.26824	5.11069	3.74444	2.90615	1.30906	1.994663	6.1509	3.69741	2.68714			allograft inflammatory factor
000024397	1562	4533	61	3262	6984	058	51132	3447	7548	K18617	Aif1	1
ENSMUSG00	1.92013	2.29018	2.62608	1.56081	1.40635	1.511727	2.3697	2.75488	2.49578			
000024766	667	8714	6526	8732	9388	902	0863	0718	5847	None	Lipo3	lipase, member O3
ENSMUSG00	3.63208	3.07139	2.48027	2.28182	1.33910	1.761578	4.9395	3.44729	2.74602			phosphoinositide-3-kinase
000025017	9792	2665	3639	2436	2938	274	78347	9753	5672	K12230	Pik3ap1	adaptor protein 1
ENSMUSG00	0.36634	0.16113	0.16198	0.02651	0.04825	0.036164	0.1952	0.13895	0.06693			
000025279	8183	8199	6255	8928	9263	277	63148	3314	4283	K11995	Dnase1l3	deoxyribonuclease 1-like 3
ENSMUSG00	6.23938	3.74676	4.10848	2.73228	2.52232	3.110600	3.3899	3.84165	4.50913			signal transducer and
000026104	4756	6931	5144	156	3785	515	95501	0434	9529	K11220	Stat1	activator of transcription 1
												CD55 molecule, decay
ENSMUSG00	3.39450	4.74215	4.10837	3.19836	2.12049	2.547953	3.8747	4.04029	3.85381			accelerating factor for
000026399	658	0929	3275	3866	6312	928	48005	8907	665	K04006	Cd55	complement
ENSMUSG00	8.99124	7.14776	6.87493	3.95009	2.54236	2.789285	8.0159	4.47348	4.06548			
000026548	6599	6323	5039	6336	1622	737	64072	2319	5371	None	Slamf9	SLAM family member 9
ENSMUSG00	2.37221	2.89898	2.42645	1.79507	1.93248	1.694749	2.3614	2.30263	2.64296			interferon induced with
000026896	6536	4063	7792	4485	9195	234	2548	9185	8076	K12647	Ifih1	helicase C domain 1
ENSMUSG00	3.36685	3.27504	2.63293	2.09710	2.04445	2.029313	3.8341	3.21133	2.57194	K06548	Siglec1	sialic acid binding Ig-like

000027322	1693	3506	7983	3714	6422	051	12133	1282	3797			lectin 1, sialoadhesin
ENSMUSG00000027514	2.62517819	1.433401168	1.176423721	0.885929994	0.649612188	0.894930794	1.285944462	1.624821443	1.720080223	K12965	Zbp1	Z-DNA binding protein 1
ENSMUSG00000027995	3.450857857	3.269986412	2.540164225	2.313884699	1.531008998	1.765702202	3.531706256	3.095152459	2.530089246	K10159	Tlr2	toll-like receptor 2
ENSMUSG00000027996	6.027485612	5.978026679	5.574874285	4.235982378	4.721921598	2.799773409	5.344165536	5.629662299	6.21831853	K02176	Sfrp2	secreted frizzled-related protein 2
ENSMUSG00000028268	11.18519601	7.766384237	6.207834499	4.036592187	3.245822313	4.14990916	4.927666647	6.534980111	8.198136932	None	Gbp3	guanylate binding protein 3
ENSMUSG00000028270	22.17151344	13.9266132	13.08865904	10.03680507	7.193104553	8.676841488	11.06411978	12.84616757	14.61135758	None	Gbp2	guanylate binding protein 2
ENSMUSG00000028581	25.7385259	21.0580142	16.17621289	14.26377869	10.28372813	12.1931011	18.79128714	16.64733482	14.2859462	K12387	Laptm5	lysosomal-associated protein transmembrane 5
ENSMUSG00000028780	1.814992146	2.421761552	2.277139933	1.691921135	1.072810313	0.932715835	1.617271116	1.881972612	2.239377799	K06840	Sema3c	sema domain, immunoglobulin domain (Ig), short basic domain, secreted, (semaphorin) 3C
ENSMUSG00000029322	2.759623825	2.163243865	1.529593995	1.007905594	0.588224544	1.215541213	3.542129289	4.028905485	4.361096644	None	Plac8	placenta-specific 8
ENSMUSG00000029368	1.233162315	14.60880911	0.542416075	0.173293503	0.144808295	0.126601638	118.2052969	54.31130828	0.140591611	K16141	Alb	albumin
ENSMUSG00000029469	8.66126392	12.82517624	12.76103604	8.133473908	6.722835529	8.515698219	9.466788725	10.17899078	10.87845463	K19677	Ift81	intraflagellar transport 81
ENSMUSG00000029669	9.468903697	11.08275877	9.371856634	6.734107969	7.47850516	7.262154659	8.934112421	9.599622487	9.659716474	K17355	Tspan12	tetraspanin 12
ENSMUSG00000030245	5.93388254	6.055240455	5.943828882	3.832847116	4.006636884	4.233921764	5.984335588	6.353348987	5.851863889	None	Golt1b	golgi transport 1B
ENSMUSG00000030474	1.790639717	1.70251621	1.645409657	1.04847724	0.707986471	0.866560479	3.412112712	2.242362721	1.340372591	None	Siglece	sialic acid binding Ig-like lectin E
ENSMUSG00000030560	16.70090872	17.53329307	17.45345468	11.08729371	10.51928827	11.43590233	19.79744537	15.73429688	14.07121182	K01275	Ctsc	cathepsin C

ENSMUSG00000030579	38.59711292	41.00089946	32.76054332	23.78537298	16.66483933	21.85436912	37.51330354	27.25362484	24.26936194	K07992	Tyrobp	TYRO protein tyrosine kinase binding protein
ENSMUSG00000030921	3.813483335	4.876046897	4.078221972	2.710657798	2.39526974	3.459840457	4.027958659	4.0147578	3.926424323	None	Trim30a	tripartite motif-containing 30A
ENSMUSG00000031389	0.610570984	0.625860552	0.350301412	0.391705492	0.145474978	0.240742088	0.507913778	0.478705558	0.479203348	K20122	Arhgap4	Rho GTPase activating protein 4
ENSMUSG00000032374	5.432377938	6.626152378	6.690161732	4.543282417	3.761378653	4.561668178	5.803774188	6.422642426	6.566392034	K13645	Plod2	procollagen lysine, 2-oxoglutarate 5-dioxygenase 2
ENSMUSG00000032915	0.584372696	0.412943486	0.277726749	0.122603895	0.046872923	0.122938857	0.445380624	0.512854123	0.455080265	K08445	Adgre4	adhesion G protein-coupled receptor E4
ENSMUSG00000033220	6.219716763	5.374642755	3.884691155	4.060817796	2.596158189	3.032605126	6.259608487	4.786984996	3.827905105	K07860	Rac2	RAS-related C3 botulinum substrate 2
ENSMUSG00000033355	12.25117241	10.04936741	8.11085641	6.582063573	4.94685076	5.063656892	7.221788674	7.806051829	8.545912889	None	Rtp4	receptor transporter protein 4
ENSMUSG00000034687	0.200474469	0.209772411	0.49153112	0.07466698	0.06195105	0.160680549	0.166029796	0.265582332	0.318779591	None	Fras1	Fraser extracellular matrix complex subunit 1
ENSMUSG00000036334	0.722992157	0.910261441	0.795697449	0.456902407	0.447866035	0.54761109	0.64343506	0.685265217	0.674293205	None	Igsf10	immunoglobulin superfamily, member 10
ENSMUSG00000036446	39.90525343	39.44314646	43.82559938	27.41914604	26.53694816	28.50677903	35.28693626	36.90169483	37.75723255	K08122	Lum	lumican
ENSMUSG00000036501	3.961582815	4.171443389	5.320406514	2.666073257	2.623616151	3.089716185	4.266382871	4.228097386	4.258037525	None	Fam13b	family with sequence similarity 13, member B
ENSMUSG00000037731	4.648833691	3.659598779	3.029451996	2.624826105	1.849445073	2.044375908	3.505704754	3.264443333	2.714025393	None	Themis2	thymocyte selection associated family member 2
ENSMUSG00000037902	17.00723457	15.25679073	13.10603152	11.46309104	10.1746919	10.64865386	16.6172052	13.93023004	11.78547488	K06551	Sirpa	signal-regulatory protein alpha
ENSMUSG00000038642	32.08588236	29.33326751	26.3759415	19.60281122	14.92626192	14.04203699	35.9688153	28.99925622	20.09598851	K01368	Ctss	cathepsin S
ENSMUSG00000038668	3.313186582	3.080683952	3.4159144	2.366346153	2.216878889	2.22079926	2.776531278	3.235866209	3.219520118	K04289	Lpar1	lysophosphatidic acid receptor 1

ENSMUSG00000039031	6.641040111	8.597990788	7.948358608	5.399519604	4.490841955	5.140907254	5.609918645	6.727655813	7.388626349	None	Arhgap18	Rho GTPase activating protein 18
ENSMUSG00000039145	1.753350636	1.660744873	1.771992617	1.17893205	1.099977285	1.426077797	1.511772598	1.615168561	1.834678118	K08794	Camk1d	calcium/calmodulin-dependent protein kinase ID
ENSMUSG00000039232	1.322347167	1.649726162	1.345954745	0.935165565	0.737193245	0.932477338	0.974469719	1.377436382	1.538051338	K08487	Stx11	syntaxin 11
ENSMUSG00000040653	36.91932472	44.36063429	41.60864727	30.68966541	26.51148267	29.7599475	42.45704922	41.8640845	41.42601393	K17556	Ppp1r14c	protein phosphatase 1, regulatory (inhibitor) subunit 14c
ENSMUSG00000040747	7.180147753	6.367040151	5.738423528	4.205813701	2.686518064	4.352567208	8.685207341	7.55500432	5.271297641	K06489	Cd53	CD53 antigen
ENSMUSG00000042302	10.10723881	14.08037683	13.35712045	8.75160154	8.301653486	9.849185902	12.91775585	13.11553873	12.90216404	None	Ehbp1	EH domain binding protein 1
ENSMUSG00000043629	0.394964176	0.492894015	0.560116952	0.221276092	0.143814005	0.227889902	0.459880688	0.362324295	0.44505875	None	1700019D03Rik	RIKEN cDNA 1700019D03 gene
ENSMUSG00000044768	0.153227549	0.135522851	0.125026394	0.093530154	0.070514158	0.094952796	0.119222685	0.135354684	0.150299111	None	D1Ertd622e	DNA segment, Chr 1, ERATO Doi 622, expressed
ENSMUSG00000044811	4.462615023	4.064317515	3.910086826	2.312179929	1.207341999	2.021253775	3.872508153	3.007871283	2.494011312	K06719	Cd300c2	CD300C molecule 2
ENSMUSG00000044951	25.04500202	25.99278652	25.46899321	16.54519018	15.76707856	18.61755369	30.53914912	26.5330765	22.46003354	K00907	Mylk4	myosin light chain kinase family, member 4
ENSMUSG00000045868	0.308250866	0.251565683	0.207909278	0.123019555	0.090856953	0.162044473	0.185943985	0.345318879	0.328145973	None	Gvin1	GTPase, very large interferon inducible 1
ENSMUSG00000046879	5.725168442	4.046033775	3.734116209	2.194452373	2.263196443	3.210633388	4.018459108	3.745929025	3.946837986	K14139	Irgm1	immunity-related GTPase family M member 1
ENSMUSG00000048865	2.899713468	3.231111067	2.28570185	1.906973635	1.330723863	1.534108024	2.753440519	2.335251047	1.709966138	None	Arhgap30	Rho GTPase activating protein 30
ENSMUSG00000051457	2.046094015	1.241885764	0.839609971	0.827352298	0.620783836	0.74148039	2.819893477	2.240579092	1.536478879	K06477	Spn	sialophorin
ENSMUSG00000052336	4.704252121	3.04481509	2.009078501	0.93020113	0.77209604	0.815119558	1.523971651	1.489059424	1.414364751	K04192	Cx3cr1	chemokine (C-X3-C motif) receptor 1

ENSMUSG00000052889	2.994627967	3.023341305	2.752560073	1.694610503	1.396390563	1.796845478	3.072703202	2.548292589	2.186351636	K19662	Prkcb	protein kinase C, beta
ENSMUSG00000053063	2.379206081	2.320485531	1.985478026	1.2460041	1.103373811	0.796497776	2.478602675	1.991668255	1.48401773	K17516	Clec12a	C-type lectin domain family 12, member a
ENSMUSG00000053318	0.826508645	0.425607473	0.584414756	0.197288677	0.039453496	0.17246516	0.738950411	0.814124668	0.497960402	K16853	Slamf8	SLAM family member 8
ENSMUSG00000054072	15.934085	13.66842967	12.97238816	9.268969502	5.127633413	8.470829981	14.93585783	15.80064183	16.69621107	None	Iigp1	interferon inducible GTPase 1
ENSMUSG00000055413	16.63489027	11.22341817	10.20907589	8.624671153	6.567983138	7.30149756	12.33781829	12.009731	9.867439255		H2-Q5	histocompatibility 2, Q region locus 5
ENSMUSG00000055541	0.999186562	0.594606925	0.543499348	0.457228577	0.249236538	0.293327169	0.801374284	0.772831142	0.618907828	K06725	Lair1	leukocyte-associated Ig-like receptor 1
ENSMUSG00000055660	1.359895744	1.537466935	1.364491304	0.856930406	0.985743211	0.982459214	1.1527887	1.414399043	1.550403635	None	Mettl4	methyltransferase like 4
ENSMUSG00000056290	0.414842442	0.496935197	0.467649878	0.159474713	0.086372855	0.181231798	0.449808932	0.356461553	0.293502149	None	Ms4a4b	membrane-spanning 4-domains, subfamily A, member 4B
ENSMUSG00000059498	26.93841705	24.87714385	22.73512984	18.39215829	14.5289914	14.69894826	34.71738276	25.11406144	16.77142066	K16824	Fcgr3	Fc receptor, IgG, low affinity III
ENSMUSG00000063388	9.894001959	2.781363913	4.904531218	1.210633374	0.640130555	1.422436981	2.534350055	2.252718648	2.304694105	None	BC023105	cDNA sequence BC023105
ENSMUSG00000063594	2.13155494	1.84279851	2.461517747	0.940681749	0.82715771	0.848926585	1.544309312	1.760344341	1.641059444	K04544	Gng8	guanine nucleotide binding protein (G protein), gamma 8
ENSMUSG00000070873	2.204185546	1.548427187	1.490118597	0.823716384	0.636222693	0.976795565	1.502820498	1.355294442	1.265524488	K06512	Lilra5	leukocyte immunoglobulin-like receptor, subfamily A (with TM domain), member 5
ENSMUSG00000071342	10.00814365	10.157596	12.39738776	7.417408268	6.843403146	8.376180837	12.26863543	11.2679232	10.64537206	None	Lsmem1	leucine-rich single-pass membrane protein 1
ENSMUSG00000074151	2.978921793	1.879036212	1.782363685	1.491753046	1.083979325	1.389443784	1.862366806	2.11804481	2.322859868		Nlrc5	NLR family, CARD domain containing 5

ENSMUSG00000079227	1.081309626	1.047780984	0.650201618	0.379476932	0.083537154	0.260835998	0.766864763	0.675772654	0.475041401	K04180	Ccr5	chemokine (C-C motif) receptor 5
ENSMUSG00000083816	0.442958437	0.361836817	0.474444955	0.119096832	0.123847397	0.05413807	0.396032542	0.56460856	0.571145107		Gm13033	prostaglandin-endoperoxide synthase 2 pseudogene
ENSMUSG00000090272	5.249254252	8.669205126	6.237663173	4.697400092	3.24682211	4.321452542	6.611749599	6.728513579	6.978923166	None	Mndal	myeloid nuclear differentiation antigen like
ENSMUSG00000091649	3.869713339	2.683591713	2.682510705	1.594831313	1.464960941	0.942455096	3.106213005	2.732463485	2.200543698	None	Phf11b	PHD finger protein 11B
ENSMUSG00000093805	0.896901332	0.99436103	1.068564379	0.545719012	0.348667731	0.620171284	1.065519588	1.038540012	0.910488215		Gal3st2b	galactose-3-O-sulfotransferase 2B
ENSMUSG00000094796	0.649996377	0.903815493	0.335373587	0.142064974	0.041036583	0.161446853	0.412420095	0.413549454	0.278891447		BC147527	cDNA sequence BC147527
ENSMUSG00000095654	1.600563924	2.173778553	1.824614644	1.099250762	0.769392854	0.840821959	1.28037221	2.083455239	2.027541153		Plscr5	phospholipid scramblase family, member 5
ENSMUSG00000101086	1.78828226	1.616850074	1.762875134	0.83206805	0.91143728	1.243074824	1.476702847	1.702885106	1.734398257		Gm28651	predicted gene 28651
ENSMUSG00000103649	2.827792728	2.421272276	2.223811402	0.993458243	0.774814168	0.999967388	2.528223525	2.974572174	2.292578426			
ENSMUSG00000105449	0.056272807	0.121853965	0.043272786	0.035846204	0.008471829	0.003703336	0.077402166	0.109768544	0.152165034			
ENSMUSG00000105504	8.164952862	5.263663026	4.121212119	3.613003437	2.683657023	2.940420782	4.219180552	5.552791166	6.014670828		Gbp5	guanylate binding protein 5
ENSMUSG00000113010	0.38647953	0.543977502	0.72119532	0.058190455	0.025213155	0.044086241	0.460715823	0.362982269	0.097915883		Gm34084	predicted gene, 34084
ENSMUSG00000113216	0.48626598	0.796533615	0.385534597	0.20828361	0.082042303	0.157799785	0.337307736	0.330715156	0.318613228		Gm40841	predicted gene, 40841
ENSMUSG00000000296	7.133561159	8.951992653	8.562619073	10.24021515	11.05901389	9.996689858	7.840869048	7.37224019	7.530465044	None	Tpd52l1	tumor protein D52-like 1
ENSMUSG00000001739	6.822305181	5.846900136	8.648168664	11.21187096	11.30463997	9.281590721	7.844546788	7.943344689	8.068716662	K06087	Cldn15	claudin 15
ENSMUSG00	53.5117	52.8090	58.3211	81.3093	95.6044	78.10630	69.504	65.0465	60.6493	K20254	Plin4	perilipin 4

000002831	0326	07	0483	399	186	406	5367	5717	5496			
ENSMUSG00000003477	25.02817991	26.46990988	30.98021296	83.92356779	82.47000452	74.70460531	59.41223561	56.89975689	52.3661326	K00562	Inmt	indolethylamine N-methyltransferase
ENSMUSG00000011305	107.4363794	100.0817258	117.21595	145.1674886	172.6801728	145.8547761	104.6817104	112.086171	116.6795691	K20255	Plin5	perilipin 5
ENSMUSG00000013275	27.97508394	27.7038909	27.91621857	41.33284849	43.08745066	41.84888963	27.64057565	31.7711778	34.28340057	K15122	Slc41a1	solute carrier family 41, member 1
ENSMUSG00000015957	3.866676742	3.930070627	3.846051615	6.0626327	6.325323663	6.002627166	4.366801007	4.214100382	4.198437867	K01384	Wnt11	wingless-type MMTV integration site family, member 11
ENSMUSG00000019066	12.52189699	10.20543415	10.68534036	15.2772856	16.95285537	13.69486989	10.58750095	10.79064274	11.7740952	K07884	Rab3d	RAB3D, member RAS oncogene family
ENSMUSG00000019139	18.37988527	17.67356689	17.7632257	22.25989413	24.10090703	21.93683929	16.77609142	16.13126547	15.93660212	K01858	Isyna1	myo-inositol 1-phosphate synthase A1
ENSMUSG00000020782	7.971964196	6.133263502	6.812392051	8.54570549	10.16592716	8.539231386	5.902277344	6.104614097	6.544749686	K06094	Llgl2	LLGL2 scribble cell polarity complex component
ENSMUSG00000021280	1.262198273	1.695659146	1.917138875	1.951996384	2.403275402	2.469435687	1.172223881	1.433578189	1.794126652	None	Exoc3l4	exocyst complex component 3-like 4
ENSMUSG00000022231	1.590331026	1.671195445	1.756840683	3.023391368	2.873310593	3.806458784	2.232368278	2.542678091	2.613268847	K06841	Sema5a	sema domain, seven thrombospondin repeats (type 1 and type 1-like), transmembrane domain (TM) and short cytoplasmic domain, (semaphorin) 5A
ENSMUSG00000022579	108.508729	87.70827727	90.42170301	116.5887337	125.6173388	112.1443743	102.1719608	89.70501813	79.89586375	K20001	Gpihbp1	GPI-anchored HDL-binding protein 1
ENSMUSG00000023439	12.40991493	9.514523069	7.298656257	11.89296064	15.17553275	13.29852131	9.459299472	8.473571352	8.829853854	K07825	Gnb3	guanine nucleotide binding protein (G protein), beta 3
ENSMUSG00000024197	72.66705816	64.5789719	72.44178743	86.14080772	104.3751968	88.01811268	59.39895977	65.43117389	69.3093886	K20287	Plin3	perilipin 3
ENSMUSG00	1.46158	1.26138	0.96946	3.20710	3.21979	2.903866	2.1118	1.86066	2.01382	K06855	Mapk4	mitogen-activated protein

000024558	8521	4451	0983	403	5037	062	59114	2549	6318			kinase 4
ENSMUSG00000024561	15.5869955	15.08531487	15.90604951	20.36645154	21.87344068	22.15237991	15.76638888	15.22426654	15.60315827	K11589	Mbd1	methyl-CpG binding domain protein 1
ENSMUSG00000024835	0.991964127	0.95054402	0.814564357	1.69358471	1.612066069	4.697940006	1.156108768	1.06474799	0.706830282	K13886	Coro1b	coronin, actin binding protein 1B
ENSMUSG00000025178	11.99916966	11.27342043	10.62997423	15.72315053	14.97911439	13.05027541	11.2116802	10.88649055	11.13048612	K13711	Pi4k2a	phosphatidylinositol 4-kinase type 2 alpha
ENSMUSG00000025509	322.3175677	289.5387776	285.927942	463.8335701	474.1939309	426.5091009	315.9424659	324.0920493	335.4092728	K16816	Pnpla2	patatin-like phospholipase domain containing 2
ENSMUSG00000025792	8.431099662	8.478461667	9.205107681	14.39934275	13.15214357	11.47024605	10.47920116	9.610890096	9.486563168	K13577	Slc25a10	solute carrier family 25 (mitochondrial carrier, dicarboxylate transporter), member 10
ENSMUSG00000027523	3.99930754	4.956771601	4.819641118	6.18742291	10.29808205	5.596260683	4.249849586	4.122222962	3.984794411	K04632	Gnas	GNAS (guanine nucleotide binding protein, alpha stimulating) complex locus
ENSMUSG00000027547	0.34939842	0.353760518	0.395417557	0.560813907	0.587054482	0.682445965	0.306489998	0.346730471	0.407114285	K19871	Sall4	spalt like transcription factor 4
ENSMUSG00000027999	17.45940222	17.55177408	16.29166591	21.2305309	22.49958879	20.46444385	15.12011741	14.44527085	15.35587438	K01047	Pla2g12a	phospholipase A2, group XIIA
ENSMUSG00000028909	2.256390215	1.93129868	1.944745601	4.06303925	4.738325973	3.793016459	3.195052469	3.110077621	3.111320042	K16662	Ptpru	protein tyrosine phosphatase, receptor type, U
ENSMUSG00000029001	11.26359267	9.74772588	11.46102332	13.47640889	14.31456516	12.9684654	9.972268292	10.4929197	10.38855936	K10103	Fbxo44	F-box protein 44
ENSMUSG00000029123	0.773704153	0.842276327	0.831463182	1.674588253	1.68460037	1.199750047	0.769560631	0.817497372	1.029107826	K08793	Stk32b	serine/threonine kinase 32B
ENSMUSG00000029161	1.252386649	1.734195498	1.066503125	2.269777492	3.063870368	2.182605209	1.098976571	1.241590669	1.630550697	None	Cgref1	cell growth regulator with EF hand domain 1
ENSMUSG00000031155	1.651725947	1.985318379	1.683889051	2.733158736	2.128434094	2.504421833	1.549852533	1.612741759	1.833397108	K08806	Pim2	proviral integration site 2

ENSMUSG00000031765	135.6844972	127.7131327	126.5198789	255.413837	245.2238383	199.8091221	164.49385	165.0403544	165.4868221	K14739	Mt1	metallothionein 1
ENSMUSG00000031808	98.73456544	98.39730907	106.6672583	149.9005242	163.9293111	150.19723	106.1107265	114.4846454	115.9487556	K08745	Slc27a1	solute carrier family 27 (fatty acid transporter), member 1
ENSMUSG00000031840	0.128958979	0.07563012	0.042972435	0.420696548	0.967498092	2.059473426	0.115297392	0.08074567	0.040840237	K07882	Rab3a	RAB3A, member RAS oncogene family
ENSMUSG00000032860	14.08375391	13.9332734	14.1236165	16.94916027	18.92299584	15.95466549	11.63843756	13.20662344	14.79466396	K04269	P2ry2	purinergic receptor P2Y, G-protein coupled 2
ENSMUSG00000033007	0.087777853	0.121342813	0.188034615	0.415369739	0.429483919	0.557863684	0.134535239	0.223768747	0.202532047	K04831	Asic4	acid-sensing (proton-gated) ion channel family member 4
ENSMUSG00000033249	3.442416214	3.823430904	2.826098624	5.023831746	5.664594051	4.689138285	3.86866176	3.829174196	3.380181289	K09417	Hsf4	heat shock transcription factor 4
ENSMUSG00000034245	12.47980715	10.31312039	11.820481	13.60871423	15.75271899	14.50550312	11.72124959	10.79758007	10.43154779	K11418	Hdac11	histone deacetylase 11
ENSMUSG00000034472	3.213419124	2.701206431	2.926835729	7.866714503	7.744924773	6.618427247	6.054576503	5.264818841	4.986549294	K07844	Rasd2	RASD family, member 2
ENSMUSG00000036181	157.441058	152.6451432	135.0803787	307.7735457	262.5266705	289.035921	192.83878	202.2123652	208.8917368	K11275	Hist1h1c	histone cluster 1, H1c
ENSMUSG00000037095	49.38121419	48.74796302	51.78721683	89.76483884	99.20273239	84.79953922	64.94005482	66.99178074	70.72357153	None	Lrg1	leucine-rich alpha-2-glycoprotein 1
ENSMUSG00000037411	17.83794067	15.70180249	20.94114837	32.39321504	34.73744671	32.56500217	17.99389834	22.24053805	26.59240597	K03982	Serpine1	serine (or cysteine) peptidase inhibitor, clade E, member 1
ENSMUSG00000038178	7.212907045	6.670481291	6.499344739	10.27872837	10.6355967	9.847002725	5.528132058	6.198487422	7.110728186	K08229	Slc43a2	solute carrier family 43, member 2
ENSMUSG00000038486	1.242358394	1.126843412	1.172530752	2.137785391	2.001054169	1.564613962	1.44879925	1.413235535	1.202327521	K06258	Sv2a	synaptic vesicle glycoprotein 2 a
ENSMUSG00000039450	13.94083252	14.90318815	13.75343597	18.73129959	18.01041283	17.02944391	10.10857897	9.6223396	9.212288394	K03331	Dcxr	dicarbonyl L-xylulose reductase
ENSMUSG00000040740	190.2388697	181.0824702	180.5984019	236.815335	253.9810578	217.5152693	140.7001275	151.1800578	165.0804583	K15117	Slc25a34	solute carrier family 25, member 34

ENSMUSG00	8.64810	6.65011	6.26734	10.6232	9.48856	9.338988	6.6325	6.63606	6.67458			transmembrane and
000041143	265	6763	4448	3643	5693	432	30523	2704	4621	None	Tmco4	coiled-coil domains 4
ENSMUSG00	7.60679	5.75719	6.63720	11.9958	10.8593	10.88975	6.8857	6.46730	6.18172			
000041889	9864	8079	5935	815	9004	152	54679	2239	4625	None	Shisa4	shisa family member 4
ENSMUSG00	23.0779	27.2995	28.6890	37.1447	36.9726	33.94143	22.516	27.0607	31.2674		A530016	RIKEN cDNA A530016L24
000043122	4972	467	8885	7486	9991	797	08761	9636	4632	None	L24Rik	gene
ENSMUSG00	2.22955	2.30816	2.22883	3.25459	3.12632	3.349694	2.5160	2.25999	2.36365			leukocyte receptor cluster
000043432	7466	3616	6965	6897	8671	06	14056	2532	3599	None	Leng9	(LRC) member 9
ENSMUSG00	5.77007	6.39567	6.62190	9.15687	10.4571	8.727736	6.6445	6.39473	5.92056			
000045573	6259	7513	6924	7893	776	911	45976	3872	5985	K18832	Penk	preproenkephalin
ENSMUSG00	2.61375	2.44513	2.71488	3.39789	3.21901	3.534227	2.2456	2.07165	2.68919		B430212	RIKEN cDNA B430212C06
000046415	8753	3591	1472	6645	7174	34	69084	1302	646		C06Rik	gene
ENSMUSG00	7.63945	6.61085	6.26730	9.18939	13.9560	9.199876	5.0298	6.37559	7.61306			RAS,
000049892	2026	1765	6921	6249	6794	171	09943	9927	6288	K07843	Rasd1	dexamethasone-induced 1
ENSMUSG00	0.87115	0.87929	0.86171	1.19430	1.12169	1.270945	0.8034	0.80096	0.80153			
000050737	969	6053	1028	5777	8471	752	67172	9594	0238	K15729	Ptges	prostaglandin E synthase
ENSMUSG00	3.31575	3.92268	3.65757	9.85563	7.10476	6.798399	5.0089	4.61754	5.59434			cytochrome c oxidase
000051811	2129	2208	8609	9499	5256	733	65428	8119	4486	K02267	Cox6b2	subunit VIb polypeptide 2
ENSMUSG00	15.2487	14.6103	14.3510	18.1628	18.6916	17.44771	14.288	13.5830	12.3726			ELMO/CED-12 domain
000056698	8682	8869	8057	128	3998	393	81938	6946	2213	None	Elmod3	containing 3
ENSMUSG00	0.21267	0.48331	0.30119	0.72048	1.26605	0.773296	0.0792	0.39949	0.42095		Gm1003	
000057913	6388	935	4184	8499	5774	489	27496	2092	5275		2	predicted gene 10032
ENSMUSG00	1.49998	1.38980	1.33978	1.99786	1.84999	1.746484	1.3569	1.35043	1.45039			
000071454	0939	3808	6551	2004	5141	374	47486	2761	2014	None	Dtnb	dystrobrevin, beta
ENSMUSG00	32.8546	35.0159	39.6635	75.2903	80.1070	69.38922	57.066	54.2974	50.2566			
000072949	1548	0604	74	2821	841	867	04613	6144	8547	K01068	Acot1	acyl-CoA thioesterase 1
ENSMUSG00	1.23966	2.95655	1.84512	6.24242	5.55329	6.245611	3.3496	2.71668	3.45480			immunoglobulin heavy
000076612	3336	2076	4505	024	0748	265	39323	6077	1437		Ighg2c	constant gamma 2C
ENSMUSG00	1.36652	1.52998	1.75149	2.68959	2.52356	3.232452	1.2983	1.89498	2.47449			
000078234	1388	1724	4143	6088	3227	623	54667	4109	8797	None	Klhdc7a	kelch domain containing 7A
ENSMUSG00	34.5860	37.6966	36.4978	54.6698	65.9938	65.63155	53.892	45.5767	39.1381	K15464	Bnip3	BCL2/adenovirus E1B

000078566	7368	186	3993	0022	239	783	33831	9236	1049		interacting protein 3
ENSMUSG00000095134	0.267975657	0.447902416	0.508989211	1.129740336	0.891592097	0.917050324	0.43125659	0.478198116	0.203677591	Mid1-ps1	midline 1, pseudogene 1
ENSMUSG00000103156	0.83608405	1.323905245	0.94725571	1.762394923	2.945400714	2.622763926	0.747511422	1.151702409	1.006167297	Gm38293	predicted gene, 38293
ENSMUSG00000116953	0.390881508	0.556241461	0.183884796	2.120447656	0.641258 32	0.295070163	0.246686478	0.237546045	0.316754012		

Table SIV. KEGG analysis of the DEGs in DbCM compared to CK.

class_A	class_B	Term	ID	Input number	Background number	P-Value	Input
Human Diseases	Cancers: Overview	Pathways in cancer	mmu 05200	47	398	4.80095 E-13	ENSMUSG00000032766|ENSMUSG00000030170|ENSMUSG00000002111|ENSMUSG00000023067|ENSMUSG00000031520|ENSMUSG00000027985|ENSMUSG00000020826|ENSMUSG00000022523|ENSMUSG00000030110|ENSMUSG00000045382|ENSMUSG00000005672|ENSMUSG00000028645|ENSMUSG00000024421|ENSMUSG00000041417|ENSMUSG00000027347|ENSMUSG00000027669|ENSMUSG00000022994|ENSMUSG00000050147|ENSMUSG00000022952|ENSMUSG00000026104|ENSMUSG00000021025|ENSMUSG00000038668|ENSMUSG00000027111|ENSMUSG00000063594|ENSMUSG00000042826|ENSMUSG00000031274|ENSMUSG00000021277|ENSMUSG00000067158|ENSMUSG00000031740|ENSMUSG00000086451|ENSMUSG00000027523|ENSMUSG00000031628|ENSMUSG00000066687|ENSMUSG00000026029|ENSMUSG00000083816|ENSMUSG00000024621|ENSMUSG00000023439|ENSMUSG00000033220|ENSMUSG00000017737|ENSMUSG00000031503|ENSMUSG00000031502|ENSMUSG00000031659|ENSMUSG00000015957|ENSMUSG00000007659|ENSMUSG00000071042|ENSMUSG00000052889|ENSMUSG00000049115
Environmental Information Processing	Signal transduction	PI3K-Akt signaling pathway	mmu 04151	42	350	5.2252E -12	ENSMUSG00000032766|ENSMUSG00000048756|ENSMUSG00000048126|ENSMUSG00000020241|ENSMUSG00000019970|ENSMUSG00000001930|ENSMUSG00000027954|ENSMUSG00000022523|ENSMUSG00000025017|ENSMUSG00000029661|ENSMUSG00000023067|ENSMUSG00000001281|ENSMUSG00000005672|ENSMUSG00000031502|ENSMUSG00000041417|ENSMUSG00000029120|ENSMUSG00000024621|ENSMUSG00000042284|ENSMUSG00000042453|ENSMUSG00000001119|ENSMUSG00000024421|ENSMUSG00000023885|ENSMUSG0

							0000038668|ENSMUSG00000001506|ENSMUSG00000027111|ENSMUSG00000063594|ENSMUSG00000042826|ENSMUSG00000031274|ENSMUSG00000031490|ENSMUSG00000026971|ENSMUSG00000020108|ENSMUSG00000067158|ENSMUSG00000022817|ENSMUSG00000031520|ENSMUSG00000023439|ENSMUSG00000027995|ENSMUSG00000031503|ENSMUSG00000055980|ENSMUSG00000027669|ENSMUSG00000007659|ENSMUSG00000092368|ENSMUSG00000039115
Organismal Systems	Digestive system	Protein digestion and absorption	mmu 04974	22	90	5.26878E-12	ENSMUSG00000048126|ENSMUSG00000020241|ENSMUSG00000058806|ENSMUSG00000029675|ENSMUSG00000061119|ENSMUSG00000010095|ENSMUSG00000029661|ENSMUSG00000026042|ENSMUSG00000026043|ENSMUSG00000001119|ENSMUSG00000001506|ENSMUSG00000027966|ENSMUSG00000031274|ENSMUSG00000028339|ENSMUSG00000026837|ENSMUSG00000067158|ENSMUSG00000004098|ENSMUSG00000000958|ENSMUSG00000033161|ENSMUSG00000022371|ENSMUSG00000031503|ENSMUSG00000031502
Metabolism	Global and overview maps	Metabolic pathways	mmu 01100	91	1298	3.36098E-11	ENSMUSG00000101111|ENSMUSG00000040364|ENSMUSG00000036880|ENSMUSG00000026003|ENSMUSG00000032420|ENSMUSG00000039450|ENSMUSG00000029925|ENSMUSG00000053604|ENSMUSG00000052102|ENSMUSG00000025509|ENSMUSG00000074207|ENSMUSG00000069835|ENSMUSG00000029330|ENSMUSG00000033793|ENSMUSG00000025190|ENSMUSG00000035824|ENSMUSG00000092329|ENSMUSG00000019916|ENSMUSG00000039347|ENSMUSG00000000320|ENSMUSG00000013584|ENSMUSG00000020638|ENSMUSG00000027227|ENSMUSG00000020534|ENSMUSG00000027875|ENSMUSG00000024843|ENSMUSG00000048087|ENSMUSG00000030972|ENSMUSG00000072949|ENSMUSG00000001270|ENSMUSG00000021608|ENSMUSG00000032315|ENSMUSG00000074264|ENSMUSG00000030483|ENSMUSG00000020407|ENSMUSG00000101249|ENSMUSG00000005547|ENSMUSG00000024827|ENSMUSG00000019139|ENSMUSG00000024903|ENSMUSG00000020520|ENSMUSG00000030541|ENSMUSG00000083816|ENSMUSG00000026687|ENSMUSG00000033308|ENSMUSG00000107653|ENSMUSG00000064367|ENSMUSG00000032271|ENSMUSG00000021456|ENSMUSG00000039497|ENSMUSG00000020826|ENSMUSG00000021226|ENSMUSG00000047250|ENSMUSG00000059447|ENSMUSG0

							0000018924|ENSMUSG00000021903|ENSMUSG00000015090|ENSMUSG00000025428|ENSMUSG00000024421|ENSMUSG00000029162|ENSMUSG00000032456|ENSMUSG00000042010|ENSMUSG00000022040|ENSMUSG00000022210|ENSMUSG00000000326|ENSMUSG00000024525|ENSMUSG00000011179|ENSMUSG00000102070|ENSMUSG00000039783|ENSMUSG00000026675|ENSMUSG00000006344|ENSMUSG00000063558|ENSMUSG00000050737|ENSMUSG00000030036|ENSMUSG00000026473|ENSMUSG00000034570|ENSMUSG00000020937|ENSMUSG00000032350|ENSMUSG00000020777|ENSMUSG00000024640|ENSMUSG00000025745|ENSMUSG00000024066|ENSMUSG00000038732|ENSMUSG00000051811|ENSMUSG00000085337|ENSMUSG00000057880|ENSMUSG00000018574|ENSMUSG00000027999|ENSMUSG00000025178|ENSMUSG00000041193|ENSMUSG00000041202
Environmental Information Processing	Signaling molecules and interaction	ECM-receptor interaction	mmu 04512	20	83	6.02895 E-11	ENSMUSG00000022817|ENSMUSG00000048126|ENSMUSG00000031274|ENSMUSG00000020241|ENSMUSG00000031503|ENSMUSG00000001930|ENSMUSG00000042284|ENSMUSG00000067158|ENSMUSG00000042453|ENSMUSG00000029661|ENSMUSG00000001119|ENSMUSG00000024421|ENSMUSG00000023885|ENSMUSG00000039115|ENSMUSG00000001281|ENSMUSG00000026971|ENSMUSG00000038486|ENSMUSG00000001506|ENSMUSG00000027111|ENSMUSG00000031502
Human Diseases	Infectious diseases: Parasitic	Leishmaniasis	mmu 05140	18	67	1.20309 E-10	ENSMUSG00000083816|ENSMUSG00000037649|ENSMUSG00000026480|ENSMUSG00000037548|ENSMUSG00000027995|ENSMUSG00000036594|ENSMUSG00000060586|ENSMUSG00000021025|ENSMUSG00000015947|ENSMUSG00000059498|ENSMUSG00000022965|ENSMUSG00000026104|ENSMUSG00000079547|ENSMUSG00000076617|ENSMUSG00000073421|ENSMUSG00000052889|ENSMUSG00000020826|ENSMUSG00000071715
Cellular Processes	Transport and catabolism	Phagosome	mmu 04145	28	192	3.65145 E-10	ENSMUSG00000053317|ENSMUSG00000059498|ENSMUSG00000060586|ENSMUSG00000020695|ENSMUSG00000052688|ENSMUSG00000030707|ENSMUSG00000079293|ENSMUSG00000037548|ENSMUSG00000015947|ENSMUSG00000079547|ENSMUSG00000038642|ENSMUSG00000022817|ENSMUSG00000071715|ENSMUSG00000026480|ENSMUSG00000043091|ENSMUSG00000015340|ENSMUSG00000023885|ENSMUSG00000058672|ENSMUSG00000055413|ENSM

							USG00000076617|ENSMUSG00000062591|ENSMUSG00000029580|ENSMUSG00000073421|ENSMUSG00000033793|ENSMUSG00000037649|ENSMUSG00000027995|ENSMUSG00000036594|ENSMUSG00000039347
Human Diseases	Infectious diseases: Bacterial	Tuberculosis	mmu 05152	27	179	3.85665E-10	ENSMUSG00000059498|ENSMUSG00000060586|ENSMUSG00000020695|ENSMUSG00000032089|ENSMUSG00000057897|ENSMUSG00000020826|ENSMUSG00000058715|ENSMUSG00000022504|ENSMUSG00000030707|ENSMUSG00000026029|ENSMUSG00000079293|ENSMUSG00000037548|ENSMUSG00000015947|ENSMUSG00000026104|ENSMUSG00000079547|ENSMUSG00000038642|ENSMUSG00000030142|ENSMUSG00000030789|ENSMUSG00000076617|ENSMUSG00000031628|ENSMUSG00000073421|ENSMUSG00000033793|ENSMUSG00000037649|ENSMUSG00000024610|ENSMUSG00000027995|ENSMUSG00000036594|ENSMUSG00000022965
Human Diseases	Infectious diseases: Viral	Herpes simplex infection	mmu 05168	30	224	5.45923E-10	ENSMUSG00000066861|ENSMUSG00000066800|ENSMUSG00000060586|ENSMUSG00000055116|ENSMUSG00000026029|ENSMUSG00000037548|ENSMUSG00000070729|ENSMUSG00000032012|ENSMUSG00000026104|ENSMUSG00000021025|ENSMUSG00000052776|ENSMUSG00000020893|ENSMUSG00000055866|ENSMUSG00000021134|ENSMUSG00000022965|ENSMUSG00000021277|ENSMUSG00000079547|ENSMUSG00000055413|ENSMUSG00000076617|ENSMUSG00000031628|ENSMUSG00000073421|ENSMUSG00000026896|ENSMUSG00000037649|ENSMUSG00000034459|ENSMUSG00000002325|ENSMUSG00000024610|ENSMUSG00000027995|ENSMUSG00000036594|ENSMUSG00000032690|ENSMUSG00000040033
Human Diseases	Infectious diseases: Parasitic	Toxoplasmosis	mmu 05145	21	113	1.2861E-09	ENSMUSG00000024421|ENSMUSG00000037649|ENSMUSG00000041417|ENSMUSG00000026029|ENSMUSG00000037548|ENSMUSG00000027995|ENSMUSG00000026104|ENSMUSG00000046879|ENSMUSG00000036594|ENSMUSG00000060586|ENSMUSG00000021025|ENSMUSG00000032089|ENSMUSG00000020826|ENSMUSG00000022965|ENSMUSG00000007659|ENSMUSG00000079547|ENSMUSG00000076617|ENSMUSG00000031628|ENSMUSG00000073421|ENSMUSG00000027111|ENSMUSG00000022504
Human Diseases	Endocrine and	AGE-RAGE	mmu 04933	18	103	4.40814E-08	ENSMUSG00000020937|ENSMUSG00000041417|ENSMUSG00000033863|ENSMUSG00000037411|ENSMUSG00000031520|ENSMUSG00000031503|ENSMUSG0

	metabolic diseases	signaling pathway in diabetic complications					0000031502|ENSMUSG00000029661|ENSMUSG00000067158|ENSMUSG00000031740|ENSMUSG00000026043|ENSMUSG00000026104|ENSMUSG00000001506|ENSMUSG00000015340|ENSMUSG00000052889|ENSMUSG00000031628|ENSMUSG00000049115|ENSMUSG00000031274
Human Diseases	Infectious diseases: Viral	Influenza A	mmu 05164	23	172	5.60364 E-08	ENSMUSG00000066861|ENSMUSG00000066800|ENSMUSG00000060586|ENSMUSG00000022504|ENSMUSG00000041417|ENSMUSG00000037548|ENSMUSG00000027509|ENSMUSG00000026104|ENSMUSG00000021025|ENSMUSG00000052776|ENSMUSG00000022965|ENSMUSG00000079547|ENSMUSG00000076617|ENSMUSG00000029580|ENSMUSG00000073421|ENSMUSG00000026896|ENSMUSG00000037649|ENSMUSG00000002325|ENSMUSG00000036594|ENSMUSG00000032690|ENSMUSG00000020641|ENSMUSG00000040033|ENSMUSG00000052889
Cellular Processes	Cellular community - eukaryotes	Focal adhesion	mmu 04510	25	203	6.44509 E-08	ENSMUSG00000048126|ENSMUSG00000020241|ENSMUSG00000001930|ENSMUSG00000029661|ENSMUSG00000001281|ENSMUSG00000041417|ENSMUSG00000042284|ENSMUSG00000042453|ENSMUSG00000001119|ENSMUSG00000024421|ENSMUSG00000023885|ENSMUSG00000044951|ENSMUSG00000001506|ENSMUSG00000027111|ENSMUSG00000031274|ENSMUSG00000067158|ENSMUSG00000029580|ENSMUSG00000026971|ENSMUSG00000022817|ENSMUSG00000031520|ENSMUSG00000033220|ENSMUSG00000031503|ENSMUSG00000031502|ENSMUSG00000039115|ENSMUSG00000052889
Organismal Systems	Development	Osteoclast differentiation	mmu 04380	20	133	7.38699 E-08	ENSMUSG00000059498|ENSMUSG00000041417|ENSMUSG00000031264|ENSMUSG00000002111|ENSMUSG00000026480|ENSMUSG00000061132|ENSMUSG00000000409|ENSMUSG00000024621|ENSMUSG00000015340|ENSMUSG00000002325|ENSMUSG00000015947|ENSMUSG00000037902|ENSMUSG00000040033|ENSMUSG00000002983|ENSMUSG00000026104|ENSMUSG00000021025|ENSMUSG00000030579|ENSMUSG00000071715|ENSMUSG00000022965|ENSMUSG00000070873
Organismal Systems	Development	Axon guidance	mmu 04360	23	176	8.16689 E-08	ENSMUSG00000030170|ENSMUSG00000028064|ENSMUSG00000003934|ENSMUSG00000027954|ENSMUSG00000029095|ENSMUSG00000057897|ENSMUSG0

							0000028780|ENSMUSG00000045382|ENSMUSG00000041417|ENSMUSG00000021904|ENSMUSG00000006494|ENSMUSG00000022231|ENSMUSG00000031398|ENSMUSG00000066877|ENSMUSG00000031558|ENSMUSG00000005958|ENSMUSG00000056427|ENSMUSG00000032475|ENSMUSG00000033220|ENSMUSG00000020902|ENSMUSG00000001227|ENSMUSG00000019647|ENSMUSG00000052133
Environmental Information Processing	Signaling molecules and interaction	Cell adhesion molecules (CAMs)	mmu04514	23	176	8.16689E-08	ENSMUSG00000060586|ENSMUSG00000001281|ENSMUSG00000001029|ENSMUSG00000037548|ENSMUSG00000032012|ENSMUSG00000071552|ENSMUSG00000079547|ENSMUSG00000076617|ENSMUSG00000048163|ENSMUSG00000027111|ENSMUSG00000055413|ENSMUSG00000016494|ENSMUSG00000073421|ENSMUSG00000053977|ENSMUSG00000047085|ENSMUSG00000037649|ENSMUSG00000036594|ENSMUSG00000027322|ENSMUSG00000051457|ENSMUSG00000021614|ENSMUSG00000039115|ENSMUSG00000021638|ENSMUSG00000001739
Organismal Systems	Immune system	Chemokine signaling pathway	mmu04062	23	198	5.44049E-07	ENSMUSG00000032766|ENSMUSG00000041112|ENSMUSG00000029417|ENSMUSG00000045382|ENSMUSG00000022994|ENSMUSG00000041417|ENSMUSG00000027669|ENSMUSG00000026104|ENSMUSG00000021025|ENSMUSG00000020676|ENSMUSG00000063594|ENSMUSG00000048756|ENSMUSG00000003283|ENSMUSG00000026180|ENSMUSG00000020395|ENSMUSG00000023439|ENSMUSG00000033220|ENSMUSG00000059456|ENSMUSG00000031659|ENSMUSG00000052336|ENSMUSG00000040033|ENSMUSG00000052889|ENSMUSG00000020143
Human Diseases	Infectious diseases: Parasitic	Amoebiasis	mmu05146	17	111	5.49253E-07	ENSMUSG00000044734|ENSMUSG00000041417|ENSMUSG00000026073|ENSMUSG00000027995|ENSMUSG00000031503|ENSMUSG00000031502|ENSMUSG00000029661|ENSMUSG00000067158|ENSMUSG00000052688|ENSMUSG00000026043|ENSMUSG00000024421|ENSMUSG00000020826|ENSMUSG00000027523|ENSMUSG00000001506|ENSMUSG00000052889|ENSMUSG00000031628|ENSMUSG00000031274
Human Diseases	Immune diseases	Asthma	mmu05310	9	26	9.40619E-07	ENSMUSG00000037649|ENSMUSG00000037548|ENSMUSG00000036594|ENSMUSG00000060586|ENSMUSG00000079547|ENSMUSG00000076617|ENSMUSG00000058715|ENSMUSG00000073421|ENSMUSG00000020676

Organismal Systems	Immune system	Leukocyte transendothelial migration	mmu 04670	17	121	1.60151E-06	ENSMUSG00000059456|ENSMUSG00000056917|ENSMUSG00000041417|ENSMUSG00000026480|ENSMUSG00000020395|ENSMUSG00000033220|ENSMUSG00000052889|ENSMUSG00000017737|ENSMUSG00000032011|ENSMUSG00000015340|ENSMUSG00000031740|ENSMUSG00000068036|ENSMUSG00000045382|ENSMUSG00000029580|ENSMUSG00000021638|ENSMUSG00000001739|ENSMUSG00000071715
Organismal Systems	Immune system	Platelet activation	mmu 04611	17	123	1.95885E-06	ENSMUSG00000031659|ENSMUSG00000041417|ENSMUSG00000031264|ENSMUSG00000029661|ENSMUSG00000050147|ENSMUSG00000047250|ENSMUSG00000001930|ENSMUSG00000027347|ENSMUSG00000034881|ENSMUSG00000026043|ENSMUSG00000058715|ENSMUSG00000027523|ENSMUSG00000044951|ENSMUSG00000029580|ENSMUSG00000022994|ENSMUSG00000001506|ENSMUSG00000029925
Organismal Systems	Environmental adaptation	Circadian entrainment	mmu 04713	15	98	2.53228E-06	ENSMUSG00000032766|ENSMUSG00000055866|ENSMUSG00000024112|ENSMUSG00000023439|ENSMUSG00000027669|ENSMUSG00000022994|ENSMUSG00000049892|ENSMUSG00000031659|ENSMUSG00000020866|ENSMUSG00000057897|ENSMUSG00000015968|ENSMUSG00000027523|ENSMUSG00000063594|ENSMUSG00000052889|ENSMUSG00000020893
Organismal Systems	Nervous system	GABAergic synapse	mmu 04727	14	89	4.09241E-06	ENSMUSG00000032766|ENSMUSG00000031340|ENSMUSG00000031343|ENSMUSG00000023439|ENSMUSG00000026473|ENSMUSG00000022994|ENSMUSG00000097675|ENSMUSG00000031659|ENSMUSG00000027669|ENSMUSG00000057880|ENSMUSG00000026407|ENSMUSG00000015968|ENSMUSG00000052889|ENSMUSG00000063594
Human Diseases	Infectious diseases: Bacterial	Staphylococcus aureus infection	mmu 05150	11	53	4.34897E-06	ENSMUSG00000037649|ENSMUSG00000037548|ENSMUSG00000036594|ENSMUSG00000060586|ENSMUSG00000015947|ENSMUSG00000059498|ENSMUSG00000056529|ENSMUSG00000079547|ENSMUSG00000076617|ENSMUSG00000048163|ENSMUSG00000073421
Human Diseases	Cardiovascular diseases	Dilated cardiomyopathy	mmu 05414	14	90	4.5941E-06	ENSMUSG00000027523|ENSMUSG00000053093|ENSMUSG00000026407|ENSMUSG00000022817|ENSMUSG00000042284|ENSMUSG00000026971|ENSMUSG00000031659|ENSMUSG00000015968|ENSMUSG00000039115|ENSMUSG00000001281|ENSMUSG00000022994|ENSMUSG00000029580|ENSMUSG00000027111|ENSMUSG00000031799

Human Diseases	Drug resistance: Antineoplastic	Platinum drug resistance	mmu 01524	13	78	5.12739 E-06	ENSMUSG00000033318|ENSMUSG00000027890|ENSMUSG00000003549|ENSMUSG00000041417|ENSMUSG00000028329|ENSMUSG00000023067|ENSMUSG00000026688|ENSMUSG00000020914|ENSMUSG00000058135|ENSMUSG00000008540|ENSMUSG00000007659|ENSMUSG00000031628|ENSMUSG00000026029
Organismal Systems	Immune system	Intestinal immune network for IgA production	mmu 04672	10	44	5.89574 E-06	ENSMUSG00000037649|ENSMUSG00000037548|ENSMUSG00000036594|ENSMUSG00000060586|ENSMUSG00000045382|ENSMUSG00000079547|ENSMUSG00000076617|ENSMUSG00000001281|ENSMUSG00000073421|ENSMUSG00000010142
Environmental Information Processing	Signal transduction	Ras signaling pathway	mmu 04014	23	231	5.90149 E-06	ENSMUSG00000032766|ENSMUSG00000052142|ENSMUSG00000027954|ENSMUSG00000022523|ENSMUSG00000005672|ENSMUSG00000041417|ENSMUSG00000027347|ENSMUSG00000027669|ENSMUSG00000024621|ENSMUSG00000032035|ENSMUSG00000063594|ENSMUSG00000042826|ENSMUSG00000068036|ENSMUSG00000030742|ENSMUSG00000031520|ENSMUSG00000023439|ENSMUSG00000027999|ENSMUSG00000033220|ENSMUSG00000041193|ENSMUSG00000041202|ENSMUSG00000007659|ENSMUSG00000071042|ENSMUSG00000052889
Human Diseases	Infectious diseases: Viral	Hepatitis C	mmu 05160	17	137	7.23607 E-06	ENSMUSG00000066861|ENSMUSG00000018899|ENSMUSG00000022383|ENSMUSG00000034459|ENSMUSG00000029120|ENSMUSG00000021277|ENSMUSG00000023067|ENSMUSG00000066800|ENSMUSG00000002325|ENSMUSG00000040033|ENSMUSG00000032690|ENSMUSG00000026104|ENSMUSG00000021025|ENSMUSG00000052776|ENSMUSG00000021638|ENSMUSG00000001739|ENSMUSG00000041417
Human Diseases	Cardiovascular diseases	Viral myocarditis	mmu 05416	14	96	8.92314 E-06	ENSMUSG00000037649|ENSMUSG00000026029|ENSMUSG00000053093|ENSMUSG00000033220|ENSMUSG00000037548|ENSMUSG00000036594|ENSMUSG00000060586|ENSMUSG00000026399|ENSMUSG00000055413|ENSMUSG00000079547|ENSMUSG00000076617|ENSMUSG00000029580|ENSMUSG00000073421|ENSMUSG00000031628
Metabolism	Lipid	Fatty	mmu	8	27	9.90594	ENSMUSG00000028937|ENSMUSG00000072949|ENSMUSG00000059447|ENSM

	metabolism	acid elongation	00062			E-06	USG00000063275|ENSMUSG00000028497|ENSMUSG00000025745|ENSMUSG00000021226|ENSMUSG00000036880
Human Diseases	Immune diseases	Inflammatory bowel disease (IBD)	mmu 05321	11	59	1.06666 E-05	ENSMUSG00000037649|ENSMUSG00000037548|ENSMUSG00000027995|ENSMUSG00000036594|ENSMUSG00000060586|ENSMUSG00000022965|ENSMUSG00000026104|ENSMUSG00000079547|ENSMUSG00000076617|ENSMUSG00000073421|ENSMUSG00000030745
Human Diseases	Infectious diseases: Viral	HTLV-I infection	mmu 05166	26	295	1.13029 E-05	ENSMUSG00000030170|ENSMUSG00000002111|ENSMUSG00000060586|ENSMUSG00000063889|ENSMUSG00000022994|ENSMUSG00000033863|ENSMUSG00000028645|ENSMUSG00000041417|ENSMUSG00000037548|ENSMUSG00000032035|ENSMUSG00000002983|ENSMUSG00000021025|ENSMUSG00000076617|ENSMUSG00000028211|ENSMUSG00000029521|ENSMUSG00000079547|ENSMUSG00000055413|ENSMUSG00000073421|ENSMUSG00000037649|ENSMUSG00000000409|ENSMUSG00000023067|ENSMUSG00000036594|ENSMUSG00000031659|ENSMUSG00000015957|ENSMUSG00000007659|ENSMUSG00000026073
Organismal Systems	Immune system	B cell receptor signaling pathway	mmu 04662	12	75	1.69239 E-05	ENSMUSG00000025491|ENSMUSG00000041417|ENSMUSG00000031264|ENSMUSG00000026288|ENSMUSG00000061132|ENSMUSG00000021025|ENSMUSG00000033220|ENSMUSG00000036526|ENSMUSG00000025017|ENSMUSG00000040592|ENSMUSG00000071042|ENSMUSG00000052889
Human Diseases	Cancers: Overview	Transcriptional misregulation in cancer	mmu 05202	19	180	1.77126 E-05	ENSMUSG00000055866|ENSMUSG00000017737|ENSMUSG00000037169|ENSMUSG00000024621|ENSMUSG00000002111|ENSMUSG00000030795|ENSMUSG00000043613|ENSMUSG00000048416|ENSMUSG00000023067|ENSMUSG00000020427|ENSMUSG00000066687|ENSMUSG00000025223|ENSMUSG00000015947|ENSMUSG00000022952|ENSMUSG00000007659|ENSMUSG00000001281|ENSMUSG00000013089|ENSMUSG00000026073|ENSMUSG00000014030
Metabolism	Lipid metabolism	Arachidonic acid metabolism	mmu 00590	13	90	2.02657 E-05	ENSMUSG00000083816|ENSMUSG00000000320|ENSMUSG00000027999|ENSMUSG00000041193|ENSMUSG00000022040|ENSMUSG00000006344|ENSMUSG00000047250|ENSMUSG00000018924|ENSMUSG00000041202|ENSMUSG00000030483|ENSMUSG00000015090|ENSMUSG00000050737|ENSMUSG000000299

<table>
<tr><td></td><td></td><td></td><td></td><td></td><td></td><td></td><td>25</td></tr>
<tr><td>Cellular Processes</td><td>Cell motility</td><td>Regulation of actin cytoskeleton</td><td>mmu 04810</td><td>21</td><td>218</td><td>2.31977 E-05</td><td>ENSMUSG00000042826|ENSMUSG00000022488|ENSMUSG00000022443|ENSMUSG00000041417|ENSMUSG00000030789|ENSMUSG00000033220|ENSMUSG00000020900|ENSMUSG00000046993|ENSMUSG00000042284|ENSMUSG00000026971|ENSMUSG00000049775|ENSMUSG00000022523|ENSMUSG00000025372|ENSMUSG00000034480|ENSMUSG00000044951|ENSMUSG00000022817|ENSMUSG00000039115|ENSMUSG00000001281|ENSMUSG00000037946|ENSMUSG00000029580|ENSMUSG00000027111</td></tr>
<tr><td>Organismal Systems</td><td>Immune system</td><td>Natural killer cell mediated cytotoxicity</td><td>mmu 04650</td><td>15</td><td>121</td><td>2.48136 E-05</td><td>ENSMUSG00000001029|ENSMUSG00000005763|ENSMUSG00000041417|ENSMUSG00000000409|ENSMUSG00000033220|ENSMUSG00000059456|ENSMUSG00000015355|ENSMUSG00000054520|ENSMUSG00000030579|ENSMUSG00000033024|ENSMUSG00000030742|ENSMUSG00000022965|ENSMUSG00000031628|ENSMUSG00000052889|ENSMUSG00000058715</td></tr>
<tr><td>Environmental Information Processing</td><td>Signal transduction</td><td>HIF-1 signaling pathway</td><td>mmu 04066</td><td>14</td><td>110</td><td>3.52656 E-05</td><td>ENSMUSG00000041417|ENSMUSG00000028645|ENSMUSG00000005413|ENSMUSG00000037411|ENSMUSG00000023067|ENSMUSG00000031490|ENSMUSG00000015340|ENSMUSG00000020826|ENSMUSG00000022965|ENSMUSG00000057897|ENSMUSG00000092368|ENSMUSG00000026773|ENSMUSG00000052889|ENSMUSG00000006494</td></tr>
<tr><td>Organismal Systems</td><td>Digestive system</td><td>Bile secretion</td><td>mmu 04976</td><td>11</td><td>71</td><td>4.90723 E-05</td><td>ENSMUSG00000021135|ENSMUSG00000037583|ENSMUSG00000060961|ENSMUSG00000031659|ENSMUSG00000029802|ENSMUSG00000023829|ENSMUSG00000027523|ENSMUSG00000033161|ENSMUSG00000022994|ENSMUSG00000038776|ENSMUSG00000028645</td></tr>
<tr><td>Human Diseases</td><td>Cancers: Specific types</td><td>Small cell lung cancer</td><td>mmu 05222</td><td>12</td><td>85</td><td>5.11077 E-05</td><td>ENSMUSG00000024421|ENSMUSG00000083816|ENSMUSG00000041417|ENSMUSG00000021277|ENSMUSG00000031503|ENSMUSG00000031502|ENSMUSG00000067158|ENSMUSG00000020826|ENSMUSG00000007659|ENSMUSG00000021025|ENSMUSG00000027111|ENSMUSG00000031274</td></tr>
<tr><td>Metabolism</td><td>Amino acid metabolism</td><td>Tryptophan metabolism</td><td>mmu 00380</td><td>9</td><td>47</td><td>5.60128 E-05</td><td>ENSMUSG00000026687|ENSMUSG00000039783|ENSMUSG00000027187|ENSMUSG00000003477|ENSMUSG00000024087|ENSMUSG00000025745|ENSMUSG00000063558|ENSMUSG00000032315|ENSMUSG00000024903</td></tr>
</table>

Organismal Systems	Endocrine system	Aldosterone synthesis and secretion	mmu 04925	12	86	5.65998 E-05	ENSMUSG00000024112|ENSMUSG00000053214|ENSMUSG00000022994|ENSMUSG00000039145|ENSMUSG00000031659|ENSMUSG00000026407|ENSMUSG00000020866|ENSMUSG00000057897|ENSMUSG00000015968|ENSMUSG00000027523|ENSMUSG00000052889|ENSMUSG00000049115
Organismal Systems	Immune system	Hematopoietic cell lineage	mmu 04640	12	87	6.25942 E-05	ENSMUSG00000030798|ENSMUSG00000026073|ENSMUSG00000024621|ENSMUSG00000042284|ENSMUSG00000060586|ENSMUSG00000015947|ENSMUSG00000026399|ENSMUSG00000005540|ENSMUSG00000016494|ENSMUSG00000005672|ENSMUSG00000053977|ENSMUSG00000027111
Environmental Information Processing	Signal transduction	Calcium signaling pathway	mmu 04020	18	182	6.35109 E-05	ENSMUSG00000019828|ENSMUSG00000020937|ENSMUSG00000024112|ENSMUSG00000086451|ENSMUSG00000059456|ENSMUSG00000015968|ENSMUSG00000031659|ENSMUSG00000020866|ENSMUSG00000026407|ENSMUSG00000034881|ENSMUSG00000057897|ENSMUSG00000020788|ENSMUSG00000020826|ENSMUSG00000044951|ENSMUSG00000056529|ENSMUSG00000052889|ENSMUSG00000049115|ENSMUSG00000027523
Organismal Systems	Nervous system	Serotonergic synapse	mmu 04726	15	133	6.66671 E-05	ENSMUSG00000061740|ENSMUSG00000032766|ENSMUSG00000083816|ENSMUSG00000023439|ENSMUSG00000027669|ENSMUSG00000026407|ENSMUSG00000000320|ENSMUSG00000047250|ENSMUSG00000018924|ENSMUSG00000015968|ENSMUSG00000027523|ENSMUSG00000031628|ENSMUSG00000052889|ENSMUSG00000060882|ENSMUSG00000063594
Organismal Systems	Nervous system	Retrograde endocannabinoid signaling	mmu 04723	13	103	7.17245 E-05	ENSMUSG00000032766|ENSMUSG00000019828|ENSMUSG00000031340|ENSMUSG00000031343|ENSMUSG00000023439|ENSMUSG00000027669|ENSMUSG00000022994|ENSMUSG00000083816|ENSMUSG00000031659|ENSMUSG00000026407|ENSMUSG00000015968|ENSMUSG00000052889|ENSMUSG00000063594
Metabolism	Lipid metabolism	Fatty acid degradation	mmu 00071	9	49	7.44547 E-05	ENSMUSG00000026687|ENSMUSG00000074207|ENSMUSG00000018574|ENSMUSG00000026003|ENSMUSG00000059447|ENSMUSG00000020777|ENSMUSG00000025745|ENSMUSG00000024900|ENSMUSG00000036880
Environmental	Signal transducti	NF-kappa B	mmu 04064	13	104	7.84094 E-05	ENSMUSG00000083816|ENSMUSG00000031264|ENSMUSG00000000409|ENSMUSG00000061132|ENSMUSG00000021277|ENSMUSG00000056130|ENSMUSG0

Information Processing	on	signaling pathway					0000021025|ENSMUSG00000036526|ENSMUSG00000002983|ENSMUSG00000007659|ENSMUSG00000030742|ENSMUSG00000052889|ENSMUSG00000015312
Metabolism	Lipid metabolism	Biosynthesis of unsaturated fatty acids	mmu 01040	7	28	8.9664E-05	ENSMUSG00000028937|ENSMUSG00000072949|ENSMUSG00000063275|ENSMUSG00000028497|ENSMUSG00000020777|ENSMUSG00000021226|ENSMUSG00000025745
Human Diseases	Cancers: Overview	Proteoglycans in cancer	mmu 05205	19	208	0.000106601	ENSMUSG00000031543|ENSMUSG00000030170|ENSMUSG00000025351|ENSMUSG00000041417|ENSMUSG00000022831|ENSMUSG00000036446|ENSMUSG00000031628|ENSMUSG00000027995|ENSMUSG00000017737|ENSMUSG00000023067|ENSMUSG00000022817|ENSMUSG00000029661|ENSMUSG00000015957|ENSMUSG00000031740|ENSMUSG00000057897|ENSMUSG00000092368|ENSMUSG00000029580|ENSMUSG00000052889|ENSMUSG00000001506
Human Diseases	Cancers: Overview	Chemical carcinogenesis	mmu 05204	12	93	0.00011137	ENSMUSG00000033318|ENSMUSG00000083816|ENSMUSG00000024087|ENSMUSG00000030711|ENSMUSG00000026688|ENSMUSG00000027890|ENSMUSG00000058135|ENSMUSG00000008540|ENSMUSG00000030483|ENSMUSG00000074207|ENSMUSG00000032315|ENSMUSG00000038776
Human Diseases	Substance dependence	Morphine addiction	mmu 05032	12	93	0.00011137	ENSMUSG00000032766|ENSMUSG00000031340|ENSMUSG00000031343|ENSMUSG00000023439|ENSMUSG00000027669|ENSMUSG00000022994|ENSMUSG00000031659|ENSMUSG00000027523|ENSMUSG00000075270|ENSMUSG00000052889|ENSMUSG00000019990|ENSMUSG00000063594
Metabolism	Global and overview maps	Fatty acid metabolism	mmu 01212	9	52	0.000111512	ENSMUSG00000063275|ENSMUSG00000018574|ENSMUSG00000026003|ENSMUSG00000059447|ENSMUSG00000028497|ENSMUSG00000020777|ENSMUSG00000036880|ENSMUSG00000024900|ENSMUSG00000025745
Organismal Systems	Endocrine system	Oxytocin signaling pathway	mmu 04921	16	158	0.00012366	ENSMUSG00000035064|ENSMUSG00000083816|ENSMUSG00000041417|ENSMUSG00000022994|ENSMUSG00000039145|ENSMUSG00000023067|ENSMUSG00000031659|ENSMUSG00000044216|ENSMUSG00000026407|ENSMUSG00000057897|ENSMUSG00000015968|ENSMUSG00000044951|ENSMUSG00000029580|ENSMUSG00000052889|ENSMUSG00000042529|ENSMUSG00000027523

Cellular Processes	Cell growth and death	p53 signaling pathway	mmu 04115	10	68	0.000157534	ENSMUSG00000036390|ENSMUSG00000026029|ENSMUSG00000037411|ENSMUSG00000023067|ENSMUSG00000020427|ENSMUSG00000026389|ENSMUSG00000019851|ENSMUSG00000031628|ENSMUSG00000015312|ENSMUSG00000029521
Environmental Information Processing	Signal transduction	Rap1 signaling pathway	mmu 04015	19	215	0.000158479	ENSMUSG00000042826|ENSMUSG00000056917|ENSMUSG00000041417|ENSMUSG00000024621|ENSMUSG00000033220|ENSMUSG00000052889|ENSMUSG00000027523|ENSMUSG00000050147|ENSMUSG00000031659|ENSMUSG00000027954|ENSMUSG00000030742|ENSMUSG00000005672|ENSMUSG00000068036|ENSMUSG00000071042|ENSMUSG00000038668|ENSMUSG00000022523|ENSMUSG00000022994|ENSMUSG00000029580|ENSMUSG00000031520
Organismal Systems	Nervous system	Cholinergic synapse	mmu 04725	13	113	0.000167123	ENSMUSG00000032766|ENSMUSG00000041417|ENSMUSG00000023439|ENSMUSG00000027669|ENSMUSG00000022994|ENSMUSG00000031659|ENSMUSG00000044216|ENSMUSG00000026407|ENSMUSG00000057897|ENSMUSG00000015968|ENSMUSG00000052889|ENSMUSG00000042529|ENSMUSG00000063594
Environmental Information Processing	Signal transduction	MAPK signaling pathway	mmu 04010	21	254	0.000173409	ENSMUSG00000042826|ENSMUSG00000036390|ENSMUSG00000024112|ENSMUSG00000031530|ENSMUSG00000033220|ENSMUSG00000061288|ENSMUSG00000015968|ENSMUSG00000028862|ENSMUSG00000027347|ENSMUSG00000022523|ENSMUSG00000026407|ENSMUSG00000020866|ENSMUSG00000037337|ENSMUSG00000002983|ENSMUSG00000031506|ENSMUSG00000071042|ENSMUSG00000031628|ENSMUSG00000026074|ENSMUSG00000052889|ENSMUSG00000026073|ENSMUSG00000015312
Organismal Systems	Immune system	Antigen processing and presentation	mmu 04612	12	98	0.000174072	ENSMUSG00000037649|ENSMUSG00000024610|ENSMUSG00000037548|ENSMUSG00000036594|ENSMUSG00000060586|ENSMUSG00000055413|ENSMUSG00000038642|ENSMUSG00000079547|ENSMUSG00000076617|ENSMUSG00000073421|ENSMUSG00000053977|ENSMUSG00000022504
Metabolism	Amino acid metabolism	Valine, leucine and isoleucin	mmu 00280	9	56	0.000183868	ENSMUSG00000022186|ENSMUSG00000026687|ENSMUSG00000027875|ENSMUSG00000059447|ENSMUSG00000057880|ENSMUSG00000025745|ENSMUSG00000063558|ENSMUSG00000024903|ENSMUSG00000036880

		e degradation					
Human Diseases	Cardiovascular diseases	Hypertrophic cardiomyopathy (HCM)	mmu 05410	11	84	0.000189313	ENSMUSG00000053093|ENSMUSG00000026407|ENSMUSG00000022817|ENSMUSG00000042284|ENSMUSG00000026971|ENSMUSG00000015968|ENSMUSG00000039115|ENSMUSG00000001281|ENSMUSG00000029580|ENSMUSG00000027111|ENSMUSG00000031799
Metabolism	Metabolism of other amino acids	Glutathione metabolism	mmu 00480	9	57	0.000207053	ENSMUSG00000033318|ENSMUSG00000030541|ENSMUSG00000027890|ENSMUSG00000006344|ENSMUSG00000026688|ENSMUSG00000058135|ENSMUSG00000008540|ENSMUSG00000011179|ENSMUSG00000032350
Human Diseases	Immune diseases	Rheumatoid arthritis	mmu 05323	11	85	0.000207849	ENSMUSG00000037649|ENSMUSG00000037548|ENSMUSG00000027995|ENSMUSG00000036594|ENSMUSG00000060586|ENSMUSG00000043613|ENSMUSG00000079547|ENSMUSG00000076617|ENSMUSG00000039347|ENSMUSG00000073421|ENSMUSG00000033793
Organismal Systems	Endocrine system	Insulin secretion	mmu 04911	11	86	0.000227897	ENSMUSG00000031840|ENSMUSG00000022994|ENSMUSG00000096146|ENSMUSG00000026407|ENSMUSG00000031659|ENSMUSG00000057897|ENSMUSG00000015968|ENSMUSG00000027523|ENSMUSG00000033161|ENSMUSG00000052889|ENSMUSG00000028645
Organismal Systems	Circulatory system	Adrenergic signaling in cardiomyocytes	mmu 04261	15	152	0.000257	ENSMUSG00000041417|ENSMUSG00000061086|ENSMUSG00000029120|ENSMUSG00000026407|ENSMUSG00000032511|ENSMUSG00000031659|ENSMUSG00000053093|ENSMUSG00000057897|ENSMUSG00000015968|ENSMUSG00000063889|ENSMUSG00000027523|ENSMUSG00000033161|ENSMUSG00000022994|ENSMUSG00000049115|ENSMUSG00000031799
Organismal Systems	Digestive system	Pancreatic secretion	mmu 04972	12	103	0.000264714	ENSMUSG00000027999|ENSMUSG00000056025|ENSMUSG00000019066|ENSMUSG00000022994|ENSMUSG00000060961|ENSMUSG00000031659|ENSMUSG00000041193|ENSMUSG00000041202|ENSMUSG00000020788|ENSMUSG00000027523|ENSMUSG00000033161|ENSMUSG00000052889
Human	Cardiovasc	Arrhyth	mmu	10	74	0.00029	ENSMUSG00000022817|ENSMUSG00000026407|ENSMUSG00000027985|ENSM

Diseases	ular diseases	mogenic right ventricular cardiomyopathy (ARVC)	05412			2067	USG00000042284|ENSMUSG00000026971|ENSMUSG00000015968|ENSMUSG00000039115|ENSMUSG00000001281|ENSMUSG00000029580|ENSMUSG00000027111
Organismal Systems	Immune system	Fc gamma R-mediated phagocytosis	mmu 04666	11	90	0.000325232	ENSMUSG00000069662|ENSMUSG00000041417|ENSMUSG00000026288|ENSMUSG00000046993|ENSMUSG00000036995|ENSMUSG00000033220|ENSMUSG00000003283|ENSMUSG00000030742|ENSMUSG00000052889|ENSMUSG00000020143|ENSMUSG00000015947
Human Diseases	Immune diseases	Primary immunodeficiency	mmu 05340	7	37	0.000400097	ENSMUSG00000031264|ENSMUSG00000000409|ENSMUSG00000061132|ENSMUSG00000029591|ENSMUSG00000010142|ENSMUSG00000053977|ENSMUSG00000022504
Human Diseases	Endocrine and metabolic diseases	Insulin resistance	mmu 04931	12	111	0.000491942	ENSMUSG00000031808|ENSMUSG00000033871|ENSMUSG00000041417|ENSMUSG00000022383|ENSMUSG00000042010|ENSMUSG00000055980|ENSMUSG00000033083|ENSMUSG00000021025|ENSMUSG00000046794|ENSMUSG00000052889|ENSMUSG00000024900|ENSMUSG00000028645
Metabolism	Xenobiotics biodegradation and metabolism	Metabolism of xenobiotics by cytochrome P450	mmu 00980	9	65	0.000494966	ENSMUSG00000033318|ENSMUSG00000027890|ENSMUSG00000024087|ENSMUSG00000074207|ENSMUSG00000026688|ENSMUSG00000058135|ENSMUSG00000008540|ENSMUSG00000032315|ENSMUSG00000038776
Organismal Systems	Circulatory system	Vascular smooth muscle contracti	mmu 04270	13	128	0.000504068	ENSMUSG00000027523|ENSMUSG00000027999|ENSMUSG00000041193|ENSMUSG00000022994|ENSMUSG00000031659|ENSMUSG00000026407|ENSMUSG00000041202|ENSMUSG00000086451|ENSMUSG00000015968|ENSMUSG00000044951|ENSMUSG00000052889|ENSMUSG00000049115|ENSMUSG000000410

<table>
<tr><th></th><th></th><th></th><th></th><th></th><th></th><th></th><th></th></tr>
<tr><td></td><td></td><td>on</td><td></td><td></td><td></td><td></td><td>46</td></tr>
<tr><td>Organismal Systems</td><td>Circulatory system</td><td>Cardiac muscle contraction</td><td>mmu 04260</td><td>10</td><td>80</td><td>0.000511475</td><td>ENSMUSG00000061086|ENSMUSG00000053093|ENSMUSG00000026407|ENSMUSG00000101249|ENSMUSG00000051811|ENSMUSG00000102070|ENSMUSG00000015968|ENSMUSG00000033161|ENSMUSG00000101111|ENSMUSG00000031799</td></tr>
<tr><td>Metabolism</td><td>Xenobiotics biodegradation and metabolism</td><td>Drug metabolism - cytochrome P450</td><td>mmu 00982</td><td>9</td><td>67</td><td>0.00060357</td><td>ENSMUSG00000028088|ENSMUSG00000033318|ENSMUSG00000040170|ENSMUSG00000074207|ENSMUSG00000026688|ENSMUSG00000027890|ENSMUSG00000058135|ENSMUSG00000008540|ENSMUSG00000063558</td></tr>
<tr><td>Organismal Systems</td><td>Nervous system</td><td>Glutamatergic synapse</td><td>mmu 04724</td><td>12</td><td>115</td><td>0.000656523</td><td>ENSMUSG00000032766|ENSMUSG00000019828|ENSMUSG00000023439|ENSMUSG00000026473|ENSMUSG00000022994|ENSMUSG00000001985|ENSMUSG00000031659|ENSMUSG00000027669|ENSMUSG00000015968|ENSMUSG00000027523|ENSMUSG00000052889|ENSMUSG00000063594</td></tr>
<tr><td>Cellular Processes</td><td>Transport and catabolism</td><td>Peroxisome</td><td>mmu 04146</td><td>10</td><td>83</td><td>0.000664197</td><td>ENSMUSG00000024066|ENSMUSG00000030541|ENSMUSG00000053898|ENSMUSG00000027187|ENSMUSG00000022040|ENSMUSG00000022210|ENSMUSG00000026853|ENSMUSG00000020777|ENSMUSG00000020826|ENSMUSG00000022982</td></tr>
<tr><td>Organismal Systems</td><td>Endocrine system</td><td>Thyroid hormone signaling pathway</td><td>mmu 04919</td><td>12</td><td>117</td><td>0.000754727</td><td>ENSMUSG00000020937|ENSMUSG00000041417|ENSMUSG00000026799|ENSMUSG00000007682|ENSMUSG00000033083|ENSMUSG00000026104|ENSMUSG00000029580|ENSMUSG00000028645|ENSMUSG00000052889|ENSMUSG00000033161|ENSMUSG00000034297|ENSMUSG00000038622</td></tr>
<tr><td>Organismal Systems</td><td>Endocrine system</td><td>PPAR signaling pathway</td><td>mmu 03320</td><td>10</td><td>85</td><td>0.000785527</td><td>ENSMUSG00000031808|ENSMUSG00000022383|ENSMUSG00000026003|ENSMUSG00000027875|ENSMUSG00000002289|ENSMUSG00000002831|ENSMUSG00000011305|ENSMUSG00000020777|ENSMUSG00000028773|ENSMUSG00000024900</td></tr>
<tr><td>Human Diseases</td><td>Cancers: Specific types</td><td>Acute myeloid leukemia</td><td>mmu 05221</td><td>8</td><td>57</td><td>0.000928978</td><td>ENSMUSG00000041417|ENSMUSG00000002111|ENSMUSG00000027985|ENSMUSG00000031490|ENSMUSG00000031155|ENSMUSG00000022952|ENSMUSG00000066687|ENSMUSG00000005672</td></tr>
<tr><td>Organismal</td><td>Endocrine</td><td>Regulatio</td><td>mmu</td><td>8</td><td>57</td><td>0.00092</td><td>ENSMUSG00000083816|ENSMUSG00000041417|ENSMUSG00000055980|ENSM</td></tr>
</table>

Systems	system	n of lipolysis in adipocytes	04923			8978	USG00000031659|ENSMUSG00000047250|ENSMUSG00000027523|ENSMUSG00000022994|ENSMUSG00000025509
Organismal Systems	Environmental adaptation	Circadian rhythm	mmu 04710	6	31	0.000935253	ENSMUSG00000055866|ENSMUSG00000020889|ENSMUSG00000068742|ENSMUSG00000030256|ENSMUSG00000055116|ENSMUSG00000020893
Human Diseases	Immune diseases	Graft-versus-host disease	mmu 05332	9	72	0.000962251	ENSMUSG00000037649|ENSMUSG00000037548|ENSMUSG00000036594|ENSMUSG00000060586|ENSMUSG00000055413|ENSMUSG00000033024|ENSMUSG00000079547|ENSMUSG00000076617|ENSMUSG00000073421
Organismal Systems	Endocrine system	GnRH signaling pathway	mmu 04912	10	88	0.001001215	ENSMUSG00000022994|ENSMUSG00000031790|ENSMUSG00000059456|ENSMUSG00000031659|ENSMUSG00000031740|ENSMUSG00000026407|ENSMUSG00000057897|ENSMUSG00000015968|ENSMUSG00000027523|ENSMUSG00000052889
Cellular Processes	Cell growth and death	Apoptosis	mmu 04210	13	139	0.001020881	ENSMUSG00000043091|ENSMUSG00000036390|ENSMUSG00000041417|ENSMUSG00000024590|ENSMUSG00000030560|ENSMUSG00000015312|ENSMUSG00000007659|ENSMUSG00000021025|ENSMUSG00000038642|ENSMUSG00000029580|ENSMUSG00000031628|ENSMUSG00000024910|ENSMUSG00000026029
Organismal Systems	Endocrine system	Ovarian steroidogenesis	mmu 04913	8	58	0.001028553	ENSMUSG00000026675|ENSMUSG00000083816|ENSMUSG00000024087|ENSMUSG00000031659|ENSMUSG00000027523|ENSMUSG00000021226|ENSMUSG00000032315|ENSMUSG00000022994
Organismal Systems	Immune system	T cell receptor signaling pathway	mmu 04660	11	105	0.001053924	ENSMUSG00000020395|ENSMUSG00000005763|ENSMUSG00000041417|ENSMUSG00000000409|ENSMUSG00000032475|ENSMUSG00000066877|ENSMUSG00000021025|ENSMUSG00000036526|ENSMUSG00000030742|ENSMUSG00000053977|ENSMUSG00000027347
Cellular Processes	Cellular community -	Tight junction	mmu 04530	13	141	0.001151162	ENSMUSG00000021638|ENSMUSG00000022831|ENSMUSG00000028906|ENSMUSG00000029120|ENSMUSG00000020900|ENSMUSG00000020782|ENSMUSG00000097652|ENSMUSG00000053093|ENSMUSG00000068036|ENSMUSG00000

	eukaryotes						029580|ENSMUSG00000052889|ENSMUSG00000022443|ENSMUSG00000001739
Human Diseases	Endocrine and metabolic diseases	Type I diabetes mellitus	mmu04940	9	77	0.001477046	ENSMUSG00000037649|ENSMUSG00000037548|ENSMUSG00000036594|ENSMUSG00000060586|ENSMUSG00000055413|ENSMUSG00000079547|ENSMUSG00000076617|ENSMUSG00000073421|ENSMUSG00000026204
Human Diseases	Infectious diseases: Viral	Hepatitis B	mmu05161	13	146	0.001538625	ENSMUSG00000041417|ENSMUSG00000059456|ENSMUSG00000027995|ENSMUSG00000017737|ENSMUSG00000023067|ENSMUSG00000056130|ENSMUSG00000026029|ENSMUSG00000040033|ENSMUSG00000026104|ENSMUSG00000021025|ENSMUSG00000031628|ENSMUSG00000052889|ENSMUSG00000026896
Metabolism	Carbohydrate metabolism	Fructose and mannose metabolism	mmu00051	6	35	0.001626927	ENSMUSG00000025648|ENSMUSG00000027227|ENSMUSG00000038028|ENSMUSG00000021456|ENSMUSG00000029162|ENSMUSG00000026773
Environmental Information Processing	Signal transduction	AMPK signaling pathway	mmu04152	12	129	0.001636744	ENSMUSG00000048756|ENSMUSG00000035064|ENSMUSG00000041417|ENSMUSG00000042010|ENSMUSG00000029120|ENSMUSG00000025648|ENSMUSG00000055980|ENSMUSG00000021456|ENSMUSG00000031490|ENSMUSG00000017950|ENSMUSG00000024900|ENSMUSG00000026773
Environmental Information Processing	Signal transduction	TNF signaling pathway	mmu04668	11	112	0.001697372	ENSMUSG00000083816|ENSMUSG00000021277|ENSMUSG00000041417|ENSMUSG00000043613|ENSMUSG00000017737|ENSMUSG00000026029|ENSMUSG00000031790|ENSMUSG00000021025|ENSMUSG00000031628|ENSMUSG00000091514|ENSMUSG00000028599
Metabolism	Amino acid metabolism	Arginine and proline metabolism	mmu00330	7	51	0.002150767	ENSMUSG00000026687|ENSMUSG00000069835|ENSMUSG00000025190|ENSMUSG00000019916|ENSMUSG00000011179|ENSMUSG00000001270|ENSMUSG00000020826
Environmental	Signal transducti	FoxO signaling	mmu04068	12	135	0.00232486	ENSMUSG00000048756|ENSMUSG00000019828|ENSMUSG00000036390|ENSMUSG00000041417|ENSMUSG00000027187|ENSMUSG00000019970|ENSMUSG0

Information Processing	on	pathway					0000078566|ENSMUSG00000055980|ENSMUSG00000023067|ENSMUSG00000025422|ENSMUSG00000097675|ENSMUSG00000015312
Cellular Processes	Cellular community - eukaryotes	Gap junction	mmu 04540	9	86	0.002944407	ENSMUSG00000043091|ENSMUSG00000019828|ENSMUSG00000027523|ENSMUSG00000052889|ENSMUSG00000031659|ENSMUSG00000058672|ENSMUSG00000038668|ENSMUSG00000062591|ENSMUSG00000022994
Metabolism	Carbohydrate metabolism	Butanoate metabolism	mmu 00650	5	27	0.002956437	ENSMUSG00000022186|ENSMUSG00000057880|ENSMUSG00000030972|ENSMUSG00000025745|ENSMUSG00000027875
Metabolism	Amino acid metabolism	Tyrosine metabolism	mmu 00350	6	40	0.00296975	ENSMUSG00000074207|ENSMUSG00000033307|ENSMUSG00000025190|ENSMUSG00000000326|ENSMUSG00000063558|ENSMUSG00000024903
Environmental Information Processing	Signal transduction	cAMP signaling pathway	mmu 04024	15	198	0.003047305	ENSMUSG00000086451|ENSMUSG00000041417|ENSMUSG00000022383|ENSMUSG00000033220|ENSMUSG00000026407|ENSMUSG00000015968|ENSMUSG00000031659|ENSMUSG00000020777|ENSMUSG00000057897|ENSMUSG00000068036|ENSMUSG00000021025|ENSMUSG00000027523|ENSMUSG00000033161|ENSMUSG00000020331|ENSMUSG00000022994
Human Diseases	Infectious diseases: Parasitic	Chagas disease (American trypanosomiasis)	mmu 05142	10	104	0.003116019	ENSMUSG00000005763|ENSMUSG00000041417|ENSMUSG00000029120|ENSMUSG00000027995|ENSMUSG00000020826|ENSMUSG00000022965|ENSMUSG00000021025|ENSMUSG00000027523|ENSMUSG00000037411|ENSMUSG00000026029
Environmental Information Processing	Signal transduction	ErbB signaling pathway	mmu 04012	9	87	0.00316079	ENSMUSG00000041417|ENSMUSG00000032475|ENSMUSG00000023067|ENSMUSG00000066877|ENSMUSG00000031490|ENSMUSG00000062991|ENSMUSG00000057897|ENSMUSG00000052889|ENSMUSG00000060275
Cellular	Transport	Lysosom	mmu	11	124	0.00352	ENSMUSG00000029426|ENSMUSG00000028164|ENSMUSG00000031903|ENSM

Processes	and catabolism	e	04142			192	USG00000026177|ENSMUSG00000028581|ENSMUSG00000024910|ENSMUSG00000038642|ENSMUSG00000020604|ENSMUSG00000025351|ENSMUSG00000030560|ENSMUSG00000033793
Human Diseases	Immune diseases	Allograft rejection	mmu 05330	8	72	0.003542564	ENSMUSG00000037649|ENSMUSG00000037548|ENSMUSG00000036594|ENSMUSG00000060586|ENSMUSG00000055413|ENSMUSG00000079547|ENSMUSG00000076617|ENSMUSG00000073421
Organismal Systems	Endocrine system	Renin secretion	mmu 04924	8	72	0.003542564	ENSMUSG00000056025|ENSMUSG00000026407|ENSMUSG00000086451|ENSMUSG00000015968|ENSMUSG00000027523|ENSMUSG00000031980|ENSMUSG00000022994|ENSMUSG00000049115
Organismal Systems	Digestive system	Gastric acid secretion	mmu 04971	8	73	0.003825338	ENSMUSG00000022994|ENSMUSG00000031659|ENSMUSG00000057897|ENSMUSG00000027523|ENSMUSG00000044951|ENSMUSG00000029580|ENSMUSG00000052889|ENSMUSG00000033161
Environmental Information Processing	Signal transduction	Phospholipase D signaling pathway	mmu 04072	12	146	0.004190783	ENSMUSG00000019828|ENSMUSG00000041417|ENSMUSG00000022994|ENSMUSG00000018008|ENSMUSG00000031659|ENSMUSG00000059456|ENSMUSG00000038668|ENSMUSG00000058715|ENSMUSG00000026180|ENSMUSG00000005672|ENSMUSG00000049115|ENSMUSG00000027523
Metabolism	Lipid metabolism	Glycerophospholipid metabolism	mmu 00564	9	94	0.005047723	ENSMUSG00000024843|ENSMUSG00000038732|ENSMUSG00000027999|ENSMUSG00000023019|ENSMUSG00000041193|ENSMUSG00000031903|ENSMUSG00000029330|ENSMUSG00000041202|ENSMUSG00000021608
Organismal Systems	Nervous system	Dopaminergic synapse	mmu 04728	11	135	0.006334589	ENSMUSG00000032766|ENSMUSG00000023439|ENSMUSG00000029120|ENSMUSG00000000326|ENSMUSG00000027669|ENSMUSG00000057897|ENSMUSG00000015968|ENSMUSG00000055116|ENSMUSG00000027523|ENSMUSG00000052889|ENSMUSG00000063594
Organismal Systems	Aging	Longevity regulating pathway -	mmu 04213	7	64	0.006713779	ENSMUSG00000048756|ENSMUSG00000041417|ENSMUSG00000027187|ENSMUSG00000055980|ENSMUSG00000031659|ENSMUSG00000022994|ENSMUSG00000022982

		multiple species					
Environmental Information Processing	Signal transduction	mTOR signaling pathway	mmu 04150	12	156	0.006785562	ENSMUSG00000030170|ENSMUSG00000041417|ENSMUSG00000020176|ENSMUSG00000010095|ENSMUSG00000019970|ENSMUSG00000055980|ENSMUSG00000031490|ENSMUSG00000015957|ENSMUSG00000092368|ENSMUSG00000020108|ENSMUSG00000052889|ENSMUSG00000033793
Organismal Systems	Endocrine system	Melanogenesis	mmu 04916	9	99	0.006859459	ENSMUSG00000030170|ENSMUSG00000022994|ENSMUSG00000005672|ENSMUSG00000027985|ENSMUSG00000031659|ENSMUSG00000015957|ENSMUSG00000057897|ENSMUSG00000027523|ENSMUSG00000052889
Human Diseases	Drug resistance: Antineoplastic	EGFR tyrosine kinase inhibitor resistance	mmu 01521	8	82	0.007222058	ENSMUSG00000048756|ENSMUSG00000041417|ENSMUSG00000031490|ENSMUSG00000062991|ENSMUSG00000007659|ENSMUSG00000092368|ENSMUSG00000052889|ENSMUSG00000060275
Environmental Information Processing	Signaling molecules and interaction	Neuroactive ligand-receptor interaction	mmu 04080	18	285	0.007480991	ENSMUSG00000048779|ENSMUSG00000019828|ENSMUSG00000023132|ENSMUSG00000031340|ENSMUSG00000031343|ENSMUSG00000032492|ENSMUSG00000022041|ENSMUSG00000034009|ENSMUSG00000050147|ENSMUSG00000044338|ENSMUSG00000032860|ENSMUSG00000043895|ENSMUSG00000034881|ENSMUSG00000086451|ENSMUSG00000038668|ENSMUSG00000056529|ENSMUSG00000001985|ENSMUSG00000049115
Organismal Systems	Immune system	Toll-like receptor signaling pathway	mmu 04620	9	101	0.007709376	ENSMUSG00000029771|ENSMUSG00000029417|ENSMUSG00000041417|ENSMUSG00000027995|ENSMUSG00000021277|ENSMUSG00000056130|ENSMUSG00000026104|ENSMUSG00000021025|ENSMUSG00000026029
Human Diseases	Infectious diseases: Viral	Measles	mmu 05162	11	139	0.007710292	ENSMUSG00000066861|ENSMUSG00000002325|ENSMUSG00000022965|ENSMUSG00000027995|ENSMUSG00000032690|ENSMUSG00000040033|ENSMUSG00000026104|ENSMUSG00000021025|ENSMUSG00000052776|ENSMUSG00000026896|ENSMUSG00000041417
Environmental	Signaling molecules	Cytokine-cytokine	mmu 04060	17	265	0.007984261	ENSMUSG00000029417|ENSMUSG00000028444|ENSMUSG00000031520|ENSMUSG00000030745|ENSMUSG00000037157|ENSMUSG00000010142|ENSMUSG0

Information Processing	and interaction	receptor interaction					0000024621|ENSMUSG00000052336|ENSMUSG00000032089|ENSMUSG00000022965|ENSMUSG00000026073|ENSMUSG00000045382|ENSMUSG00000026180|ENSMUSG00000044337|ENSMUSG00000005672|ENSMUSG00000020676|ENSMUSG00000028599
Organismal Systems	Excretory system	Proximal tubule bicarbonate reclamation	mmu 04964	4	22	0.008211243	ENSMUSG00000025792|ENSMUSG00000060961|ENSMUSG00000033161|ENSMUSG00000000805
Environmental Information Processing	Signal transduction	Jak-STAT signaling pathway	mmu 04630	12	161	0.008487866	ENSMUSG00000028444|ENSMUSG00000041417|ENSMUSG00000002325|ENSMUSG00000063558|ENSMUSG00000023067|ENSMUSG00000037157|ENSMUSG00000032089|ENSMUSG00000040033|ENSMUSG00000026104|ENSMUSG00000007659|ENSMUSG00000022965|ENSMUSG00000030745
Organismal Systems	Endocrine system	Renin-angiotensin system	mmu 04614	5	36	0.008735496	ENSMUSG00000061119|ENSMUSG00000028024|ENSMUSG00000053719|ENSMUSG00000031980|ENSMUSG00000049115
Human Diseases	Immune diseases	Autoimmune thyroid disease	mmu 05320	8	87	0.009893284	ENSMUSG00000037649|ENSMUSG00000037548|ENSMUSG00000036594|ENSMUSG00000060586|ENSMUSG00000055413|ENSMUSG00000079547|ENSMUSG00000076617|ENSMUSG00000073421
Organismal Systems	Immune system	Complement and coagulation cascades	mmu 04610	8	87	0.009893284	ENSMUSG00000044206|ENSMUSG00000030789|ENSMUSG00000037411|ENSMUSG00000050147|ENSMUSG00000001930|ENSMUSG00000079105|ENSMUSG00000026399|ENSMUSG00000030111
Organismal Systems	Excretory system	Endocrine and other factor-re	mmu 04961	6	54	0.010992145	ENSMUSG00000022994|ENSMUSG00000032492|ENSMUSG00000053719|ENSMUSG00000027523|ENSMUSG00000033161|ENSMUSG00000052889

		gulated calcium reabsorption					
Metabolism	Metabolism of cofactors and vitamins	Retinol metabolism	mmu 00830	8	89	0.011144954	ENSMUSG00000013584|ENSMUSG00000074207|ENSMUSG00000022210|ENSMUSG00000005547|ENSMUSG00000030483|ENSMUSG00000063558|ENSMUSG00000032315|ENSMUSG00000056666
Metabolism	Lipid metabolism	alpha-Linolenic acid metabolism	mmu 00592	4	25	0.012117184	ENSMUSG00000041193|ENSMUSG00000020777|ENSMUSG00000027999|ENSMUSG00000041202
Human Diseases	Immune diseases	Systemic lupus erythematosus	mmu 05322	11	149	0.012168512	ENSMUSG00000037649|ENSMUSG00000037548|ENSMUSG00000068854|ENSMUSG00000036594|ENSMUSG00000060586|ENSMUSG00000079105|ENSMUSG00000015947|ENSMUSG00000079547|ENSMUSG00000076617|ENSMUSG00000073421|ENSMUSG00000093577
Human Diseases	Neurodegenerative diseases	Parkinson's disease	mmu 05012	11	149	0.012168512	ENSMUSG00000032596|ENSMUSG00000029223|ENSMUSG00000101111|ENSMUSG00000101249|ENSMUSG00000051811|ENSMUSG00000044285|ENSMUSG00000064367|ENSMUSG00000102070|ENSMUSG00000023826|ENSMUSG00000031628|ENSMUSG00000025428
Organismal Systems	Endocrine system	Adipocytokine signaling pathway	mmu 04920	7	73	0.012619589	ENSMUSG00000022383|ENSMUSG00000042010|ENSMUSG00000055980|ENSMUSG00000021025|ENSMUSG00000028645|ENSMUSG00000024900|ENSMUSG00000028599
Organismal Systems	Excretory system	Aldosterone-regulated sodium reabsorp	mmu 04960	5	40	0.012861752	ENSMUSG00000019970|ENSMUSG00000033161|ENSMUSG00000052889|ENSMUSG00000041417|ENSMUSG00000055980

		tion					
Organismal Systems	Digestive system	Fat digestion and absorption	mmu04975	5	40	0.012861752	ENSMUSG00000041193|ENSMUSG00000052396|ENSMUSG00000028158|ENSMUSG00000041202|ENSMUSG00000027999
Cellular Processes	Cellular community - eukaryotes	Adherens junction	mmu04520	7	74	0.013451477	ENSMUSG00000033220|ENSMUSG00000027985|ENSMUSG00000032012|ENSMUSG00000006411|ENSMUSG00000025372|ENSMUSG00000068036|ENSMUSG00000029580
Environmental Information Processing	Signal transduction	Hippo signaling pathway -multiple species	mmu04392	4	26	0.01364248	ENSMUSG00000042129|ENSMUSG00000036862|ENSMUSG00000027339|ENSMUSG00000025239
Environmental Information Processing	Signal transduction	cGMP-PKG signaling pathway	mmu04022	12	173	0.013932619	ENSMUSG00000041417|ENSMUSG00000053093|ENSMUSG00000026407|ENSMUSG00000015968|ENSMUSG00000055980|ENSMUSG00000031659|ENSMUSG00000086451|ENSMUSG00000020788|ENSMUSG00000044951|ENSMUSG00000033161|ENSMUSG00000022994|ENSMUSG00000049115
Human Diseases	Drug resistance: Antineoplastic	Endocrine resistance	mmu01522	8	95	0.015600811	ENSMUSG00000041417|ENSMUSG00000017737|ENSMUSG00000023067|ENSMUSG00000031659|ENSMUSG00000031740|ENSMUSG00000027523|ENSMUSG00000014773|ENSMUSG00000022994
Organismal Systems	Aging	Longevity regulating pathway	mmu04211	8	96	0.016452845	ENSMUSG00000048756|ENSMUSG00000041417|ENSMUSG00000027187|ENSMUSG00000055980|ENSMUSG00000031659|ENSMUSG00000031490|ENSMUSG00000037204|ENSMUSG00000022994
Environmental	Signal transducti	Phosphatidylinosit	mmu04070	8	97	0.017337913	ENSMUSG00000041417|ENSMUSG00000026288|ENSMUSG00000034570|ENSMUSG00000020937|ENSMUSG00000024525|ENSMUSG00000025178|ENSMUSG0

Information Processing	on	ol signaling system					0000029330|ENSMUSG00000052889
Metabolism	Lipid metabolism	Ether lipid metabolism	mmu 00565	5	44	0.018136244	ENSMUSG00000041193|ENSMUSG00000022425|ENSMUSG00000041202|ENSMUSG00000027999|ENSMUSG00000021608
Organismal Systems	Endocrine system	Estrogen signaling pathway	mmu 04915	8	98	0.018256664	ENSMUSG00000019828|ENSMUSG00000041417|ENSMUSG00000017737|ENSMUSG00000031659|ENSMUSG00000031740|ENSMUSG00000024222|ENSMUSG00000027523|ENSMUSG00000022994
Metabolism	Global and overview maps	Carbon metabolism	mmu 01200	9	118	0.018516813	ENSMUSG00000030541|ENSMUSG00000027187|ENSMUSG00000025190|ENSMUSG00000021456|ENSMUSG00000053604|ENSMUSG00000024827|ENSMUSG00000024640|ENSMUSG00000020534|ENSMUSG00000025745
Organismal Systems	Digestive system	Carbohydrate digestion and absorption	mmu 04973	5	45	0.019647567	ENSMUSG00000074264|ENSMUSG00000033161|ENSMUSG00000052889|ENSMUSG00000041417|ENSMUSG00000015968
Metabolism	Carbohydrate metabolism	Glyoxylate and dicarboxylate metabolism	mmu 00630	4	30	0.020934627	ENSMUSG00000026473|ENSMUSG00000020534|ENSMUSG00000024827|ENSMUSG00000027187
Metabolism	Energy metabolism	Nitrogen metabolism	mmu 00910	3	17	0.023367473	ENSMUSG00000026473|ENSMUSG00000038526|ENSMUSG00000000805
Human Diseases	Cancers: Overview	Central carbon metaboli	mmu 05230	6	66	0.025113944	ENSMUSG00000041417|ENSMUSG00000038028|ENSMUSG00000030110|ENSMUSG00000006494|ENSMUSG00000005672|ENSMUSG00000028645

		sm in cancer					
Organismal Systems	Sensory system	Inflammatory mediator regulation of TRP channels	mmu 04750	9	126	0.026295449	ENSMUSG00000041417|ENSMUSG00000022994|ENSMUSG00000000320|ENSMUSG00000032860|ENSMUSG00000031659|ENSMUSG00000033007|ENSMUSG00000057897|ENSMUSG00000027523|ENSMUSG00000052889
Metabolism	Metabolism of other amino acids	beta-Alanine metabolism	mmu 00410	4	33	0.027713119	ENSMUSG00000057880|ENSMUSG00000026687|ENSMUSG00000025745|ENSMUSG00000033308
Human Diseases	Endocrine and metabolic diseases	Type II diabetes mellitus	mmu 04930	5	50	0.028433803	ENSMUSG00000015968|ENSMUSG00000020866|ENSMUSG00000041417|ENSMUSG00000096146|ENSMUSG00000055980
Organismal Systems	Immune system	RIG-I-like receptor signaling pathway	mmu 04622	6	69	0.029953187	ENSMUSG00000024349|ENSMUSG00000026029|ENSMUSG00000021277|ENSMUSG00000021025|ENSMUSG00000026896|ENSMUSG00000035692
Organismal Systems	Immune system	Fc epsilon RI signaling pathway	mmu 04664	6	69	0.029953187	ENSMUSG00000041417|ENSMUSG00000031264|ENSMUSG00000026288|ENSMUSG00000033220|ENSMUSG00000030742|ENSMUSG00000058715
Metabolism	Metabolism of cofactors and vitamins	One carbon pool by folate	mmu 00670	3	19	0.03014849	ENSMUSG00000085337|ENSMUSG00000020256|ENSMUSG00000020534
Metabolism	Carbohydr	Inositol	mmu	6	70	0.03169	ENSMUSG00000026288|ENSMUSG00000034570|ENSMUSG00000020937|ENSM

	ate metabolism	phosphate metabolism	00562			267	USG00000024525|ENSMUSG00000025178|ENSMUSG00000019139
Human Diseases	Neurodegenerative diseases	Amyotrophic lateral sclerosis (ALS)	mmu 05014	5	52	0.03254431	ENSMUSG00000007659|ENSMUSG00000031628|ENSMUSG00000028599|ENSMUSG00000022982|ENSMUSG00000027187
Metabolism	Metabolism of cofactors and vitamins	Nicotinate and nicotinamide metabolism	mmu 00760	4	35	0.032877928	ENSMUSG00000032271|ENSMUSG00000032456|ENSMUSG00000032420|ENSMUSG00000063558
Organismal Systems	Endocrine system	Thyroid hormone synthesis	mmu 04918	6	71	0.033496605	ENSMUSG00000022994|ENSMUSG00000031659|ENSMUSG00000027523|ENSMUSG00000025823|ENSMUSG00000052889|ENSMUSG00000033161
Metabolism	Amino acid metabolism	Arginine biosynthesis	mmu 00220	3	20	0.033885144	ENSMUSG00000026473|ENSMUSG00000025190|ENSMUSG00000020826
Metabolism	Amino acid metabolism	Lysine degradation	mmu 00310	5	53	0.034731167	ENSMUSG00000032649|ENSMUSG00000019055|ENSMUSG00000026687|ENSMUSG00000025745|ENSMUSG00000032374
Human Diseases	Neurodegenerative diseases	Alzheimer's disease	mmu 05010	11	177	0.0349667	ENSMUSG00000052593|ENSMUSG00000101111|ENSMUSG00000026407|ENSMUSG00000015968|ENSMUSG00000101249|ENSMUSG00000051811|ENSMUSG00000102070|ENSMUSG00000020788|ENSMUSG00000031628|ENSMUSG00000025428|ENSMUSG00000026029
Metabolism	Amino	Alanine,	mmu	4	38	0.04160	ENSMUSG00000026473|ENSMUSG00000025190|ENSMUSG00000057880|ENSM

	acid metabolism	aspartate and glutamate metabolism	00250			9355	USG00000024903
Metabolism	Metabolism of cofactors and vitamins	Vitamin B6 metabolism	mmu 00750	2	9	0.044549492	ENSMUSG00000024640|ENSMUSG00000063558
Metabolism	Amino acid metabolism	Phenylalanine, tyrosine and tryptophan biosynthesis	mmu 00400	2	9	0.044549492	ENSMUSG00000025190|ENSMUSG00000024903
Human Diseases	Infectious diseases: Bacterial	Legionellosis	mmu 05134	5	58	0.047007675	ENSMUSG00000021025|ENSMUSG00000027995|ENSMUSG00000078566|ENSMUSG00000031628|ENSMUSG00000026029
Metabolism	Global and overview maps	Biosynthesis of amino acids	mmu 01230	6	79	0.05031902	ENSMUSG00000030541|ENSMUSG00000026473|ENSMUSG00000020534|ENSMUSG00000024640|ENSMUSG00000025190|ENSMUSG00000053604
Metabolism	Amino acid metabolism	Phenylalanine metabolism	mmu 00360	3	24	0.051083659	ENSMUSG00000033307|ENSMUSG00000025190|ENSMUSG00000024903
Genetic	Folding,	Protein	mmu	10	167	0.05226	ENSMUSG00000029657|ENSMUSG00000053317|ENSMUSG00000032115|ENSM

Information Processing	sorting and degradation	processing in endoplasmic reticulum	04141			0332	USG00000051984|ENSMUSG00000031770|ENSMUSG00000041556|ENSMUSG00000023826|ENSMUSG00000025823|ENSMUSG00000028410|ENSMUSG00000030036
Environmental Information Processing	Signal transduction	Wnt signaling pathway	mmu 04310	9	146	0.055198957	ENSMUSG00000030170|ENSMUSG00000015957|ENSMUSG00000033220|ENSMUSG00000027996|ENSMUSG00000034574|ENSMUSG00000027985|ENSMUSG00000028988|ENSMUSG00000057897|ENSMUSG00000052889
Metabolism	Nucleotide metabolism	Pyrimidine metabolism	mmu 00240	7	104	0.059746631	ENSMUSG00000033308|ENSMUSG00000020407|ENSMUSG00000035824|ENSMUSG00000032420|ENSMUSG00000020638|ENSMUSG00000075704|ENSMUSG00000021236
Metabolism	Lipid metabolism	Synthesis and degradation of ketone bodies	mmu 00072	2	11	0.060642642	ENSMUSG00000022186|ENSMUSG00000027875
Cellular Processes	Transport and catabolism	Endocytosis	mmu 04144	15	294	0.063155641	ENSMUSG00000018008|ENSMUSG00000026979|ENSMUSG00000030110|ENSMUSG00000046993|ENSMUSG00000024621|ENSMUSG00000036995|ENSMUSG00000027293|ENSMUSG00000055413|ENSMUSG00000025422|ENSMUSG00000045382|ENSMUSG00000075284|ENSMUSG00000026180|ENSMUSG00000005672|ENSMUSG00000040797|ENSMUSG00000056515
Human Diseases	Cancers: Overview	Viral carcinogenesis	mmu 05203	13	245	0.063334912	ENSMUSG00000021277|ENSMUSG00000041417|ENSMUSG00000002325|ENSMUSG00000023067|ENSMUSG00000068854|ENSMUSG00000031161|ENSMUSG00000034245|ENSMUSG00000055413|ENSMUSG00000004698|ENSMUSG00000021025|ENSMUSG00000031628|ENSMUSG00000111815|ENSMUSG00000026029
Environmen	Membran	ABC	mmu	4	46	0.07065	ENSMUSG00000020620|ENSMUSG00000035722|ENSMUSG00000029802|ENSM

tal Information Processing	e transport	transporters	02010			0491	USG00000041797
Organismal Systems	Digestive system	Mineral absorption	mmu 04978	4	46	0.070650491	ENSMUSG00000026177|ENSMUSG00000033161|ENSMUSG00000031765|ENSMUSG00000005413
Metabolism	Lipid metabolism	Steroid hormone biosynthesis	mmu 00140	6	87	0.071521838	ENSMUSG00000061740|ENSMUSG00000026675|ENSMUSG00000024087|ENSMUSG00000000326|ENSMUSG00000030483|ENSMUSG00000032315
Metabolism	Glycan biosynthesis and metabolism	Mucin type O-Glycan biosynthesis	mmu 00512	3	28	0.071704429	ENSMUSG00000021903|ENSMUSG00000092329|ENSMUSG00000020520
Metabolism	Nucleotide metabolism	Purine metabolism	mmu 00230	10	179	0.074115922	ENSMUSG00000024066|ENSMUSG00000032565|ENSMUSG00000100075|ENSMUSG00000031659|ENSMUSG00000032420|ENSMUSG00000085337|ENSMUSG00000021236|ENSMUSG00000075270|ENSMUSG00000022994|ENSMUSG00000019990
Human Diseases	Endocrine and metabolic diseases	Non-alcoholic fatty liver disease (NAFLD)	mmu 04932	9	157	0.077542754	ENSMUSG00000101111|ENSMUSG00000022383|ENSMUSG00000055980|ENSMUSG00000051811|ENSMUSG00000101249|ENSMUSG00000026029|ENSMUSG00000102070|ENSMUSG00000031628|ENSMUSG00000041417
Metabolism	Amino acid metabolism	Cysteine and methionine metabolism	mmu 00270	4	49	0.083634635	ENSMUSG00000025190|ENSMUSG00000032350|ENSMUSG00000024903|ENSMUSG00000048087

Metabolism	Lipid metabolism	Linoleic acid metabolism	mmu 00591	4	50	0.088204734	ENSMUSG00000018924|ENSMUSG00000041193|ENSMUSG00000041202|ENSMUSG00000027999
Metabolism	Carbohydrate metabolism	Propanoate metabolism	mmu 00640	3	31	0.089227714	ENSMUSG00000057880|ENSMUSG00000025745|ENSMUSG00000042010
Metabolism	Energy metabolism	Oxidative phosphorylation	mmu 00190	8	139	0.091003169	ENSMUSG00000101111|ENSMUSG00000101249|ENSMUSG00000051811|ENSMUSG00000064367|ENSMUSG00000102070|ENSMUSG00000039347|ENSMUSG00000025428|ENSMUSG00000033793
Metabolism	Xenobiotics biodegradation and metabolism	Drug metabolism - other enzymes	mmu 00983	4	51	0.09289265	ENSMUSG00000024066|ENSMUSG00000020407|ENSMUSG00000035824|ENSMUSG00000033308
Cellular Processes	Cell growth and death	Apoptosis - multiple species	mmu 04215	3	32	0.095430324	ENSMUSG00000031628|ENSMUSG00000026029|ENSMUSG00000007659
Human Diseases	Cancers: Specific types	Chronic myeloid leukemia	mmu 05220	5	73	0.097376443	ENSMUSG00000021025|ENSMUSG00000023067|ENSMUSG00000041417|ENSMUSG00000022952|ENSMUSG00000007659
Human Diseases	Neurodegenerative diseases	Prion diseases	mmu 05020	3	34	0.108340868	ENSMUSG00000079105|ENSMUSG00000022982|ENSMUSG00000033863
Metabolism	Metabolism of other amino acids	Selenocompound metabolism	mmu 00450	2	17	0.117733694	ENSMUSG00000075704|ENSMUSG00000003477

Human Diseases	Infectious diseases: Bacterial	Bacterial invasion of epithelial cells	mmu 05100	5	78	0.118491681	ENSMUSG00000041112|ENSMUSG00000029580|ENSMUSG00000046993|ENSMUSG00000022831|ENSMUSG00000041417
Organismal Systems	Digestive system	Salivary secretion	mmu 04970	5	78	0.118491681	ENSMUSG00000052889|ENSMUSG00000033161|ENSMUSG00000022994|ENSMUSG00000031659|ENSMUSG00000027523
Human Diseases	Infectious diseases: Bacterial	Salmonella infection	mmu 05132	5	78	0.118491681	ENSMUSG00000020826|ENSMUSG00000029580|ENSMUSG00000052688|ENSMUSG00000022965|ENSMUSG00000046993
Human Diseases	Cancers: Overview	Choline metabolism in cancer	mmu 05231	6	101	0.119115289	ENSMUSG00000024843|ENSMUSG00000041417|ENSMUSG00000033220|ENSMUSG00000031490|ENSMUSG00000023829|ENSMUSG00000052889
Organismal Systems	Endocrine system	Glucagon signaling pathway	mmu 04922	6	102	0.123002553	ENSMUSG00000022383|ENSMUSG00000042010|ENSMUSG00000057897|ENSMUSG00000027523|ENSMUSG00000024900|ENSMUSG00000028645
Metabolism	Lipid metabolism	Glycerolipid metabolism	mmu 00561	4	59	0.134363749	ENSMUSG00000052396|ENSMUSG00000026687|ENSMUSG00000038732|ENSMUSG00000025509
Human Diseases	Substance dependence	Alcoholism	mmu 05034	10	204	0.135616793	ENSMUSG00000032766|ENSMUSG00000023439|ENSMUSG00000027669|ENSMUSG00000068854|ENSMUSG00000031161|ENSMUSG00000034245|ENSMUSG00000004698|ENSMUSG00000027523|ENSMUSG00000093577|ENSMUSG00000063594
Environmental Information Processing	Signal transduction	Hippo signaling pathway	mmu 04390	8	154	0.136509672	ENSMUSG00000030170|ENSMUSG00000025239|ENSMUSG00000029120|ENSMUSG00000037411|ENSMUSG00000027985|ENSMUSG00000015957|ENSMUSG00000029580|ENSMUSG00000020782
Metabolism	Lipid metabolis	Steroid biosynth	mmu 00100	2	19	0.138911117	ENSMUSG00000026675|ENSMUSG00000107653

	m	esis					
Environmental Information Processing	Signal transduction	VEGF signaling pathway	mmu 04370	4	60	0.140005701	ENSMUSG00000033220|ENSMUSG00000083816|ENSMUSG00000052889|ENSMUSG00000041417
Metabolism	Carbohydrate metabolism	Pyruvate metabolism	mmu 00620	3	39	0.143264409	ENSMUSG00000061046|ENSMUSG00000026687|ENSMUSG00000042010
Metabolism	Global and overview maps	2-Oxocarboxylic acid metabolism	mmu 01210	2	20	0.14978738	ENSMUSG00000030541|ENSMUSG00000025190
Metabolism	Glycan biosynthesis and metabolism	Glycosaminoglycan biosynthesis - chondroitin sulfate / dermatan sulfate	mmu 00532	2	20	0.14978738	ENSMUSG00000037347|ENSMUSG00000039497
Metabolism	Amino acid metabolism	Glycine, serine and threonine metabolism	mmu 00260	3	41	0.158154988	ENSMUSG00000020534|ENSMUSG00000024827|ENSMUSG00000024640

Human Diseases	Cancers: Specific types	Bladder cancer	mmu 05219	3	41	0.158154988	ENSMUSG00000031740|ENSMUSG00000017737|ENSMUSG00000023067
Human Diseases	Cancers: Specific types	Colorectal cancer	mmu 05210	4	64	0.163471362	ENSMUSG00000033220|ENSMUSG00000031628|ENSMUSG00000041417|ENSMUSG00000027985
Human Diseases	Cancers: Specific types	Glioma	mmu 05214	4	65	0.169547497	ENSMUSG00000023067|ENSMUSG00000052889|ENSMUSG00000041417|ENSMUSG00000057897
Organismal Systems	Excretory system	Vasopressin-regulated water reabsorption	mmu 04962	3	43	0.173493093	ENSMUSG00000009013|ENSMUSG00000022994|ENSMUSG00000027523
Human Diseases	Cancers: Specific types	Pancreatic cancer	mmu 05212	4	66	0.175701503	ENSMUSG00000033220|ENSMUSG00000026104|ENSMUSG00000041417|ENSMUSG00000007659
Organismal Systems	Endocrine system	Progesterone-mediated oocyte maturation	mmu 04914	5	90	0.17679362	ENSMUSG00000025586|ENSMUSG00000022994|ENSMUSG00000111815|ENSMUSG00000031659|ENSMUSG00000041417
Organismal Systems	Endocrine system	Insulin signaling pathway	mmu 04910	7	142	0.186542333	ENSMUSG00000041417|ENSMUSG00000042010|ENSMUSG00000055980|ENSMUSG00000021456|ENSMUSG00000031490|ENSMUSG00000092368|ENSMUSG00000046794
Human Diseases	Substance dependence	Amphetamine addiction	mmu 05031	4	68	0.188230908	ENSMUSG00000027523|ENSMUSG00000052889|ENSMUSG00000057897|ENSMUSG00000015968
Metabolism	Biosynthe	Caffeine	mmu	1	6	0.19938	ENSMUSG00000024066

	sis of other secondary metabolites	metabolism	00232			173	
Human Diseases	Neurodegenerative diseases	Huntington's disease	mmu 05016	9	199	0.20548027	ENSMUSG00000101111|ENSMUSG00000101249|ENSMUSG00000051811|ENSMUSG00000022982|ENSMUSG00000102070|ENSMUSG00000031628|ENSMUSG00000033826|ENSMUSG00000025428|ENSMUSG00000026029
Human Diseases	Cancers: Specific types	Melanoma	mmu 05218	4	72	0.214089016	ENSMUSG00000042826|ENSMUSG00000022523|ENSMUSG00000023067|ENSMUSG00000041417
Organismal Systems	Development	Dorso-ventral axis formation	mmu 04320	2	26	0.217760711	ENSMUSG00000025586|ENSMUSG00000032035
Environmental Information Processing	Signal transduction	Notch signaling pathway	mmu 04330	3	49	0.221679521	ENSMUSG00000049502|ENSMUSG00000052593|ENSMUSG00000014773
Metabolism	Carbohydrate metabolism	Amino sugar and nucleotide sugar metabolism	mmu 00520	3	49	0.221679521	ENSMUSG00000048065|ENSMUSG00000036820|ENSMUSG00000052102
Organismal Systems	Endocrine system	Prolactin signaling pathway	mmu 04917	4	74	0.227368285	ENSMUSG00000048756|ENSMUSG00000026104|ENSMUSG00000018899|ENSMUSG00000041417
Human Diseases	Infectious diseases:	Pertussis	mmu 05133	4	74	0.227368285	ENSMUSG00000020826|ENSMUSG00000031628|ENSMUSG00000056130|ENSMUSG00000018899

Environmental Information Processing	Bacterial Signal transduction	Sphingolipid signaling pathway	mmu 04071	6	125	0.227439811	ENSMUSG00000041417|ENSMUSG00000029120|ENSMUSG00000033220|ENSMUSG00000058715|ENSMUSG00000052889|ENSMUSG00000043895
Cellular Processes	Cell growth and death	Cell cycle	mmu 04110	6	125	0.227439811	ENSMUSG00000036390|ENSMUSG00000029521|ENSMUSG00000023067|ENSMUSG00000032411|ENSMUSG00000111815|ENSMUSG00000015312
Human Diseases	Infectious diseases: Viral	Epstein-Barr virus infection	mmu 05169	10	232	0.228038458	ENSMUSG00000041417|ENSMUSG00000002111|ENSMUSG00000021277|ENSMUSG00000023067|ENSMUSG00000051457|ENSMUSG00000032089|ENSMUSG00000005540|ENSMUSG00000002983|ENSMUSG00000021025|ENSMUSG00000055413
Human Diseases	Endocrine and metabolic diseases	Maturity onset diabetes of the young	mmu 04950	2	27	0.229373784	ENSMUSG00000026398|ENSMUSG00000017950
Human Diseases	Cancers: Specific types	Endometrial cancer	mmu 05213	3	52	0.246689826	ENSMUSG00000048756|ENSMUSG00000041417|ENSMUSG00000027985
Human Diseases	Cancers: Specific types	Thyroid cancer	mmu 05216	2	29	0.252703222	ENSMUSG00000027985|ENSMUSG00000030110
Human Diseases	Cancers: Specific types	Basal cell carcinoma	mmu 05217	3	55	0.272102809	ENSMUSG00000030170|ENSMUSG00000027985|ENSMUSG00000015957
Metabolism	Carbohydrate metabolism	Pentose phosphate pathway	mmu 00030	2	31	0.276082592	ENSMUSG00000053604|ENSMUSG00000021456
Human	Cancers:	Non-sma	mmu	3	56	0.28063	ENSMUSG00000048756|ENSMUSG00000052889|ENSMUSG00000041417

Diseases	Specific types	ll cell lung cancer	05223			8023	
Organismal Systems	Immune system	NOD-like receptor signaling pathway	mmu 04621	3	59	0.30636 5057	ENSMUSG00000021025|ENSMUSG00000021709|ENSMUSG00000026029
Metabolism	Metabolis m of cofactors and vitamins	Ubiquino ne and other terpenoi d-quinon e biosynth esis	mmu 00130	1	11	0.31698 0393	ENSMUSG00000003849
Metabolism	Metabolis m of other amino acids	Taurine and hypotaur ine metaboli sm	mmu 00430	1	11	0.31698 0393	ENSMUSG00000006344
Genetic Information Processing	Replicatio n and repair	Base excision repair	mmu 03410	2	35	0.32262 4687	ENSMUSG00000029591|ENSMUSG00000035121
Organismal Systems	Nervous system	Long-ter m depressi on	mmu 04730	3	61	0.32356 9684	ENSMUSG00000027523|ENSMUSG00000019828|ENSMUSG00000052889
Organismal Systems	Sensory system	Taste transduc tion	mmu 04742	4	89	0.33171 7525	ENSMUSG00000019828|ENSMUSG00000022994|ENSMUSG00000023439|ENSM USG00000031343

Human Diseases	Cancers: Specific types	Prostate cancer	mmu 05215	4	89	0.331717525	ENSMUSG00000021025|ENSMUSG00000023067|ENSMUSG00000041417|ENSMUSG00000027985
Organismal Systems	Nervous system	Synaptic vesicle cycle	mmu 04721	3	62	0.332174329	ENSMUSG00000039347|ENSMUSG00000031840|ENSMUSG00000033793
Metabolism	Carbohydrate metabolism	Pentose and glucuronate interconversions	mmu 00040	2	36	0.334157388	ENSMUSG00000039450|ENSMUSG00000027227
Organismal Systems	Immune system	Cytosolic DNA-sensing pathway	mmu 04623	3	64	0.349366907	ENSMUSG00000021025|ENSMUSG00000024349|ENSMUSG00000027514
Organismal Systems	Nervous system	Neurotrophin signaling pathway	mmu 04722	5	122	0.365319181	ENSMUSG00000021025|ENSMUSG00000048756|ENSMUSG00000041417|ENSMUSG00000057897|ENSMUSG00000055980
Organismal Systems	Nervous system	Long-term potentiation	mmu 04720	3	66	0.366510302	ENSMUSG00000019828|ENSMUSG00000052889|ENSMUSG00000057897
Metabolism	Carbohydrate metabolism	Glycolysis / Gluconeogenesis	mmu 00010	3	66	0.366510302	ENSMUSG00000026687|ENSMUSG00000074207|ENSMUSG00000021456
Metabolism	Lipid metabolism	Fatty acid biosynth	mmu 00061	1	14	0.379082988	ENSMUSG00000042010

		esis					
Human Diseases	Substance dependence	Nicotine addiction	mmu 05033	2	40	0.379630765	ENSMUSG00000031340|ENSMUSG00000031343
Human Diseases	Cancers: Specific types	Renal cell carcinoma	mmu 05211	3	68	0.38357414	ENSMUSG00000028645|ENSMUSG00000041417|ENSMUSG00000032035
Metabolism	Metabolism of cofactors and vitamins	Porphyrin and chlorophyll metabolism	mmu 00860	2	41	0.390800535	ENSMUSG00000005413|ENSMUSG00000003617
Metabolism	Glycan biosynthesis and metabolism	Glycosphingolipid biosynthesis - globo series	mmu 00603	1	15	0.398503665	ENSMUSG00000040364
Genetic Information Processing	Replication and repair	Nucleotide excision repair	mmu 03420	2	44	0.423740983	ENSMUSG00000028329|ENSMUSG00000003549
Human Diseases	Cancers: Overview	MicroRNAs in cancer	mmu 05206	10	281	0.425770494	ENSMUSG00000069662|ENSMUSG00000083816|ENSMUSG00000024087|ENSMUSG00000017737|ENSMUSG00000023067|ENSMUSG00000055980|ENSMUSG00000022510|ENSMUSG00000020108|ENSMUSG00000031628|ENSMUSG00000052889
Metabolism	Metabolism of cofactors	Pantothenate and CoA	mmu 00770	1	18	0.45319918	ENSMUSG00000033308

	and vitamins	biosynthesis					
Metabolism	Glycan biosynthesis and metabolism	Other glycan degradation	mmu 00511	1	18	0.45319918	ENSMUSG00000028164
Cellular Processes	Cellular community - eukaryotes	Signaling pathways regulating pluripotency of stem cells	mmu 04550	5	140	0.476022145	ENSMUSG00000030170|ENSMUSG00000027796|ENSMUSG00000015957|ENSMUSG00000041417|ENSMUSG00000021255
Human Diseases	Infectious diseases: Parasitic	Malaria	mmu 05144	2	50	0.486672521	ENSMUSG00000023885|ENSMUSG00000027995
Genetic Information Processing	Replication and repair	Fanconi anemia pathway	mmu 03460	2	51	0.496739592	ENSMUSG00000026429|ENSMUSG00000003549
Cellular Processes	Cell growth and death	Oocyte meiosis	mmu 04114	4	116	0.519317783	ENSMUSG00000025586|ENSMUSG00000022994|ENSMUSG00000057897|ENSMUSG00000031659
Metabolism	Metabolism of terpenoids and polyketides	Terpenoid backbone biosynthesis	mmu 00900	1	24	0.548131561	ENSMUSG00000027875

Metabolism	Amino acid metabolism	Histidine metabolism	mmu 00340	1	26	0.575963267	ENSMUSG00000026687
Metabolism	Glycan biosynthesis and metabolism	Glycosphingolipid biosynthesis - lacto and neolacto series	mmu 00601	1	26	0.575963267	ENSMUSG00000040364
Organismal Systems	Excretory system	Collecting duct acid secretion	mmu 04966	1	27	0.589230028	ENSMUSG00000039347
Metabolism	Carbohydrate metabolism	Ascorbate and aldarate metabolism	mmu 00053	1	27	0.589230028	ENSMUSG00000026687
Genetic Information Processing	Transcription	Spliceosome	mmu 03040	4	135	0.635252462	ENSMUSG00000021134|ENSMUSG00000022774|ENSMUSG00000070729|ENSMUSG00000031134
Metabolism	Glycan biosynthesis and metabolism	Other types of O-glycan biosynthesis	mmu 00514	1	31	0.6382771	ENSMUSG00000032649
Genetic Information Processing	Folding, sorting and	Protein export	mmu 03060	1	31	0.6382771	ENSMUSG00000053317

	degradation						
Metabolism	Carbohydrate metabolism	Citrate cycle (TCA cycle)	mmu00020	1	32	0.649595678	ENSMUSG00000030541
Genetic Information Processing	Folding, sorting and degradation	SNARE interactions in vesicular transport	mmu04130	1	33	0.660560365	ENSMUSG00000039232
Genetic Information Processing	Folding, sorting and degradation	Ubiquitin mediated proteolysis	mmu04120	4	145	0.6884502	ENSMUSG00000095134|ENSMUSG00000023826|ENSMUSG00000032596|ENSMUSG00000041556
Human Diseases	Infectious diseases: Parasitic	African trypanosomiasis	mmu05143	1	37	0.701096207	ENSMUSG00000052889
Cellular Processes	Transport and catabolism	Regulation of autophagy	mmu04140	1	40	0.728290772	ENSMUSG00000097675
Genetic Information Processing	Folding, sorting and degradation	RNA degradation	mmu03018	2	83	0.748755276	ENSMUSG00000032565|ENSMUSG00000022863
Genetic Information Processing	Folding, sorting and	Proteasome	mmu03050	1	45	0.768232633	ENSMUSG00000024338

	degradation						
Metabolism	Glycan biosynthesis and metabolism	N-Glycan biosynthesis	mmu 00510	1	49	0.795918203	ENSMUSG00000030036
Human Diseases	Substance dependence	Cocaine addiction	mmu 05030	1	49	0.795918203	ENSMUSG00000027523
Genetic Information Processing	Translation	RNA transport	mmu 03013	4	170	0.796547945	ENSMUSG00000062309|ENSMUSG00000022774|ENSMUSG00000031490|ENSMUSG00000027509
Genetic Information Processing	Translation	mRNA surveillance pathway	mmu 03015	2	96	0.815236554	ENSMUSG00000029120|ENSMUSG00000022774
Metabolism	Carbohydrate metabolism	Starch and sucrose metabolism	mmu 00500	1	54	0.82592503	ENSMUSG00000074264
Genetic Information Processing	Translation	Ribosome	mmu 03010	3	148	0.854572768	ENSMUSG00000092368|ENSMUSG00000047676|ENSMUSG00000109695
Genetic Information Processing	Translation	Ribosome biogenesis in eukaryotes	mmu 03008	1	83	0.930821906	ENSMUSG00000062309

Environmental Information Processing	Signal transduction	TGF-beta signaling pathway	mmu 04350	1	85	0.935088968	ENSMUSG00000027796
Organismal Systems	Sensory system	Olfactory transduction	mmu 04740	2	1102	1	ENSMUSG00000045392|ENSMUSG00000057897

Table SV. KEGG analysis of the DEGs in SPM compared to DbCM.

class_A	class_B	Term	ID	Input number	Background number	P-Value	Input
Metabolism	Global and overview maps	Metabolic pathways	mmu01100	122	1298	5.72202E-21	ENSMUSG00000029759|ENSMUSG00000023262|ENSMUSG00000046598|ENSMUSG00000061518|ENSMUSG00000058624|ENSMUSG00000074064|ENSMUSG00000029455|ENSMUSG00000024978|ENSMUSG00000064345|ENSMUSG00000064356|ENSMUSG00000025991|ENSMUSG00000022474|ENSMUSG00000023070|ENSMUSG00000078592|ENSMUSG00000056973|ENSMUSG00000016252|ENSMUSG00000026005|ENSMUSG00000039450|ENSMUSG00000055978|ENSMUSG00000011752|ENSMUSG00000025509|ENSMUSG00000031278|ENSMUSG00000020182|ENSMUSG00000031173|ENSMUSG00000019987|ENSMUSG00000064341|ENSMUSG00000025495|ENSMUSG00000001670|ENSMUSG00000010651|ENSMUSG00000025059|ENSMUSG00000028838|ENSMUSG00000071072|ENSMUSG00000052160|ENSMUSG00000028413|ENSMUSG00000044986|ENSMUSG00000037798|ENSMUSG00000029201|ENSMUSG00000035834|ENSMUSG00000025204|ENSMUSG00000022853|ENSMUSG00000072949|ENSMUSG00000003500|ENSMUSG00000032315|ENSMUSG00000102070|ENSMUSG00000029545|ENSMUSG00000028541|ENSMUSG00000028011|ENSMUSG00000030660|ENSMUSG00000021235|ENSMUSG00000028393|ENSMUSG00000022546|ENSMUSG00000083816|ENSMUSG00000003053|ENSMUSG00000028124|ENSMUSG00000032478|ENSMUSG00000057880|ENSMUSG00000042642|ENSMUSG00000020649|ENSMUSG00000021033|ENSMUSG00000001891|ENSMUSG00000035273|ENSMUSG00000027427|ENSMUSG00000080845|ENSMUSG00000019139|ENSMUSG00000025651|ENSMUSG00000029780|ENSMUS

							G00000067736|ENSMUSG00000064368|ENSMUSG00000018924|ENSMUSG00000027332|ENSMUSG00000032263|ENSMUSG00000024747|ENSMUSG00000071711|ENSMUSG00000022292|ENSMUSG00000027296|ENSMUSG00000006442|ENSMUSG00000044894|ENSMUSG00000029376|ENSMUSG00000079941|ENSMUSG00000030630|ENSMUSG00000084093|ENSMUSG00000042462|ENSMUSG00000042460|ENSMUSG00000060376|ENSMUSG00000021913|ENSMUSG00000020333|ENSMUSG00000026675|ENSMUSG00000029575|ENSMUSG00000020471|ENSMUSG00000050737|ENSMUSG00000032123|ENSMUSG00000032997|ENSMUSG00000027822|ENSMUSG00000027195|ENSMUSG00000041193|ENSMUSG00000024885|ENSMUSG00000010607|ENSMUSG00000078636|ENSMUSG00000030826|ENSMUSG00000038843|ENSMUSG00000032527|ENSMUSG00000022821|ENSMUSG00000020163|ENSMUSG00000003809|ENSMUSG00000100131|ENSMUSG00000051811|ENSMUSG00000021273|ENSMUSG00000064367|ENSMUSG00000064360|ENSMUSG00000064363|ENSMUSG00000031749|ENSMUSG00000030747|ENSMUSG00000047379|ENSMUSG00000027999|ENSMUSG00000057342|ENSMUSG00000017713|ENSMUSG00000025178|ENSMUSG00000024892|ENSMUSG00000040774|ENSMUSG00000041202|ENSMUSG00000025579|ENSMUSG00000022947
Organismal Systems	Immune system	Complement and coagulation cascades	mmu04610	24	87	3.49218E-13	ENSMUSG00000026715|ENSMUSG00000039109|ENSMUSG00000028001|ENSMUSG00000021999|ENSMUSG00000036887|ENSMUSG00000031444|ENSMUSG00000021492|ENSMUSG00000026579|ENSMUSG00000071178|ENSMUSG00000026399|ENSMUSG00000033831|ENSMUSG00000040552|ENSMUSG00000059481|ENSMUSG00000033860|ENSMUSG00000024164|ENSMUSG00000026874|ENSMUSG00000036896|ENSMUSG00000022181|ENSMUSG00000000290|ENSMUSG00000037411|ENSMUSG00000066366|ENSMUSG00000049130|ENSMUSG00000027249|ENSMUSG00000038224

Human Diseases	Infectious diseases: Bacterial	Staphylococcus aureus infection	mmu05150	14	53	4.52642E-08	ENSMUSG00000059498|ENSMUSG00000030830|ENSMUSG00000059481|ENSMUSG00000033860|ENSMUSG00000000290|ENSMUSG00000059089|ENSMUSG00000024164|ENSMUSG00000049130|ENSMUSG00000026874|ENSMUSG00000094694|ENSMUSG00000026656|ENSMUSG00000036887|ENSMUSG00000040552|ENSMUSG00000036896
Metabolism	Energy metabolism	Oxidative phosphorylation	mmu00190	20	139	5.03457E-07	ENSMUSG00000079941|ENSMUSG00000025651|ENSMUSG00000020163|ENSMUSG00000102070|ENSMUSG00000084093|ENSMUSG00000064360|ENSMUSG00000100131|ENSMUSG00000025204|ENSMUSG00000051811|ENSMUSG00000067736|ENSMUSG00000064367|ENSMUSG00000074218|ENSMUSG00000064341|ENSMUSG00000064363|ENSMUSG00000064345|ENSMUSG00000016252|ENSMUSG00000061518|ENSMUSG00000064368|ENSMUSG00000064356|ENSMUSG00000044894
Human Diseases	Infectious diseases: Parasitic	Leishmaniasis	mmu05140	14	67	5.42103E-07	ENSMUSG00000083816|ENSMUSG00000059498|ENSMUSG00000094694|ENSMUSG00000024164|ENSMUSG00000004266|ENSMUSG00000059089|ENSMUSG00000027995|ENSMUSG00000000290|ENSMUSG00000027164|ENSMUSG00000027009|ENSMUSG00000030595|ENSMUSG00000026104|ENSMUSG00000052889|ENSMUSG00000039005
Organismal Systems	Development	Osteoclast differentiation	mmu04380	19	133	1.06081E-06	ENSMUSG00000058818|ENSMUSG00000054594|ENSMUSG00000059498|ENSMUSG00000081665|ENSMUSG00000002111|ENSMUSG00000002325|ENSMUSG00000059089|ENSMUSG00000026104|ENSMUSG00000027164|ENSMUSG00000015340|ENSMUSG00000007613|ENSMUSG00000037902|ENSMUSG00000030579|ENSMUSG00000026656|ENSMUSG00000021936|ENSMUSG00000015837|ENSMUSG00000081723|ENSMUSG00000026072|ENSMUSG00000070873
Human Diseases	Neurodegenerative diseases	Parkinson's disease	mmu05012	20	149	1.33683E-06	ENSMUSG00000079941|ENSMUSG00000025651|ENSMUSG00000020163|ENSMUSG00000102070|ENSMUSG00000084093|ENSMUSG00000064360|ENSMUSG00000100131|ENSMUSG00000025204|ENSMUSG00000051811|ENSMUSG00000067736|ENSMUSG00000064367|E

								NSMUSG00000074218|ENSMUSG00000064341|ENSMUSG00000064363|ENSMUSG00000064345|ENSMUSG00000016252|ENSMUSG00000061518|ENSMUSG00000064368|ENSMUSG00000064356|ENSMUSG00000044894
Organismal Systems	Endocrine system	PPAR signaling pathway	mmu03320	15	85	1.41679E-06		ENSMUSG00000031808|ENSMUSG00000010651|ENSMUSG00000031278|ENSMUSG00000005681|ENSMUSG00000025059|ENSMUSG00000019505|ENSMUSG00000011305|ENSMUSG00000002289|ENSMUSG00000002831|ENSMUSG00000050195|ENSMUSG00000022853|ENSMUSG00000028494|ENSMUSG00000032083|ENSMUSG00000020333|ENSMUSG00000027359
Metabolism	Global and overview maps	Biosynthesis of amino acids	mmu01230	14	79	3.02194E-06		ENSMUSG00000025991|ENSMUSG00000022546|ENSMUSG00000001670|ENSMUSG00000078592|ENSMUSG00000037798|ENSMUSG00000017713|ENSMUSG00000030826|ENSMUSG00000023262|ENSMUSG00000078636|ENSMUSG00000031173|ENSMUSG00000026005|ENSMUSG00000024892|ENSMUSG00000019987|ENSMUSG00000011752
Organismal Systems	Immune system	Chemokine signaling pathway	mmu04062	22	198	6.74872E-06		ENSMUSG00000068798|ENSMUSG00000019122|ENSMUSG00000034116|ENSMUSG00000023078|ENSMUSG00000027646|ENSMUSG00000030595|ENSMUSG00000026104|ENSMUSG00000060509|ENSMUSG00000035373|ENSMUSG00000029684|ENSMUSG00000049103|ENSMUSG00000035352|ENSMUSG00000003283|ENSMUSG00000043004|ENSMUSG00000023439|ENSMUSG00000033220|ENSMUSG00000028874|ENSMUSG00000052336|ENSMUSG00000021508|ENSMUSG00000063594|ENSMUSG00000052889|ENSMUSG00000020143
Cellular Processes	Cell motility	Regulation of actin cytoskeleton	mmu04810	23	218	9.13466E-06		ENSMUSG00000103735|ENSMUSG00000022523|ENSMUSG00000006457|ENSMUSG00000029684|ENSMUSG00000027009|ENSMUSG00000045613|ENSMUSG00000027646|ENSMUSG00000014956|ENSMUSG00000020135|ENSMUSG00000057967|ENSMUSG00000044951|ENSMUSG00000005947|ENSMUSG00000034116|ENSMUSG00000021676|ENSMUSG00000062929|ENSMUSG00000022488|ENSMUSG00000030830|ENSMUSG00000038387|ENSMUSG00000030739|ENSMUS

							G00000000290|ENSMUSG00000033220|ENSMUSG00000027249|ENSMUSG00000019907
Human Diseases	Infectious diseases: Viral	Influenza A	mmu05164	20	172	9.50885E-06	ENSMUSG00000032661|ENSMUSG00000025888|ENSMUSG00000044583|ENSMUSG00000059481|ENSMUSG00000002325|ENSMUSG00000026104|ENSMUSG00000024079|ENSMUSG00000042349|ENSMUSG00000059970|ENSMUSG00000032109|ENSMUSG00000037523|ENSMUSG00000039304|ENSMUSG00000030595|ENSMUSG00000018932|ENSMUSG00000035352|ENSMUSG00000021936|ENSMUSG00000024810|ENSMUSG00000052889|ENSMUSG00000039005|ENSMUSG00000026896
Metabolism	Amino acid metabolism	Valine, leucine and isoleucine degradation	mmu00280	11	56	1.47065E-05	ENSMUSG00000029482|ENSMUSG00000010651|ENSMUSG00000029545|ENSMUSG00000022853|ENSMUSG00000030826|ENSMUSG00000027332|ENSMUSG00000057880|ENSMUSG00000032263|ENSMUSG00000029455|ENSMUSG00000032527|ENSMUSG00000060376
Organismal Systems	Circulatory system	Cardiac muscle contraction	mmu04260	13	80	1.54671E-05	ENSMUSG00000079941|ENSMUSG00000025651|ENSMUSG00000020163|ENSMUSG00000084093|ENSMUSG00000053093|ENSMUSG00000061518|ENSMUSG00000051811|ENSMUSG00000074218|ENSMUSG00000044894|ENSMUSG00000102070|ENSMUSG00000015968|ENSMUSG00000054640|ENSMUSG00000020882
Human Diseases	Infectious diseases: Viral	Measles	mmu05162	17	139	2.41985E-05	ENSMUSG00000032661|ENSMUSG00000094694|ENSMUSG00000039304|ENSMUSG00000002325|ENSMUSG00000026656|ENSMUSG00000027995|ENSMUSG00000026104|ENSMUSG00000024079|ENSMUSG00000042349|ENSMUSG00000059970|ENSMUSG00000037523|ENSMUSG00000027164|ENSMUSG00000030595|ENSMUSG00000004043|ENSMUSG00000044583|ENSMUSG00000039005|ENSMUSG00000026896
Organismal Systems	Immune system	B cell receptor signaling pathway	mmu04662	12	75	3.73224E-05	ENSMUSG00000058818|ENSMUSG00000025491|ENSMUSG00000094694|ENSMUSG00000004266|ENSMUSG00000026656|ENSMUSG00000028459|ENSMUSG00000028159|ENSMUSG00000033220|ENSMUSG00000025017|ENSMUSG00000030595|ENSMUSG00000052889|ENSMUSG00000034116

Cellular Processes	Transport and catabolism	Phagosome	mmu04145	20	192	4.02838E-05	ENSMUSG00000059498|ENSMUSG00000067235|ENSMUSG00000026712|ENSMUSG00000036499|ENSMUSG00000024164|ENSMUSG00000059089|ENSMUSG00000027995|ENSMUSG00000000290|ENSMUSG00000015340|ENSMUSG00000058672|ENSMUSG00000025044|ENSMUSG00000055413|ENSMUSG00000022797|ENSMUSG00000094694|ENSMUSG00000026656|ENSMUSG00000103735|ENSMUSG00000038642|ENSMUSG00000026202|ENSMUSG00000026390|ENSMUSG00000039005
Human Diseases	Infectious diseases: Bacterial	Tuberculosis	mmu05152	19	179	4.93092E-05	ENSMUSG00000026104|ENSMUSG00000059498|ENSMUSG00000026712|ENSMUSG00000036499|ENSMUSG00000057342|ENSMUSG00000059089|ENSMUSG00000027995|ENSMUSG00000044827|ENSMUSG00000000290|ENSMUSG00000027646|ENSMUSG00000027164|ENSMUSG00000026656|ENSMUSG00000056501|ENSMUSG00000094694|ENSMUSG00000021936|ENSMUSG00000038642|ENSMUSG00000024164|ENSMUSG00000055994|ENSMUSG00000039005
Environmental Information Processing	Signaling molecules and interaction	Cytokine-cytokine receptor interaction	mmu04060	24	265	5.78552E-05	ENSMUSG00000040329|ENSMUSG00000067336|ENSMUSG00000019122|ENSMUSG00000024793|ENSMUSG00000057722|ENSMUSG00000007613|ENSMUSG00000023078|ENSMUSG00000021796|ENSMUSG00000019966|ENSMUSG00000049103|ENSMUSG00000060509|ENSMUSG00000071713|ENSMUSG00000071714|ENSMUSG00000035373|ENSMUSG00000006235|ENSMUSG00000035352|ENSMUSG00000037035|ENSMUSG00000060548|ENSMUSG00000017057|ENSMUSG00000052336|ENSMUSG00000021508|ENSMUSG00000039304|ENSMUSG00000028859|ENSMUSG00000026072
Metabolism	Amino acid metabolism	Tryptophan metabolism	mmu00380	9	47	0.000103933	ENSMUSG00000024087|ENSMUSG00000028011|ENSMUSG00000003809|ENSMUSG00000020182|ENSMUSG00000003477|ENSMUSG00000022853|ENSMUSG00000029455|ENSMUSG00000021913|ENSMUSG00000032315
Human Diseases	Infectious diseases: Viral	Herpes simplex infection	mmu05168	21	224	0.000105012	ENSMUSG00000079339|ENSMUSG00000032661|ENSMUSG00000067235|ENSMUSG00000024164|ENSMUSG00000002325|ENSMUSG00000027995|ENSMUSG00000024079|ENSMUSG00000042349|ENSMU

							SG00000014956|ENSMUSG00000062300|ENSMUSG00000037523|ENSMUSG00000026874|ENSMUSG00000055413|ENSMUSG00000027164|ENSMUSG00000030595|ENSMUSG00000026104|ENSMUSG00000035352|ENSMUSG00000021936|ENSMUSG00000055116|ENSMUSG00000046245|ENSMUSG00000026896
Organismal Systems	Nervous system	Serotonergic synapse	mmu04726	15	133	0.000162192	ENSMUSG00000026824|ENSMUSG00000094806|ENSMUSG00000083816|ENSMUSG00000043004|ENSMUSG00000023439|ENSMUSG00000068086|ENSMUSG00000020182|ENSMUSG00000063594|ENSMUSG00000018924|ENSMUSG00000003053|ENSMUSG00000015968|ENSMUSG00000027523|ENSMUSG00000004113|ENSMUSG00000052889|ENSMUSG00000032839
Human Diseases	Cancers: Overview	Pathways in cancer	mmu05200	30	398	0.000167946	ENSMUSG00000002111|ENSMUSG00000022523|ENSMUSG00000046532|ENSMUSG00000015957|ENSMUSG00000020135|ENSMUSG00000057967|ENSMUSG00000020048|ENSMUSG00000019966|ENSMUSG00000026104|ENSMUSG00000038668|ENSMUSG00000036904|ENSMUSG00000079465|ENSMUSG00000063594|ENSMUSG00000017386|ENSMUSG00000025860|ENSMUSG00000040760|ENSMUSG00000021270|ENSMUSG00000001552|ENSMUSG00000027523|ENSMUSG00000083816|ENSMUSG00000006586|ENSMUSG00000023439|ENSMUSG00000033220|ENSMUSG00000004043|ENSMUSG00000027164|ENSMUSG00000007613|ENSMUSG00000021936|ENSMUSG00000028859|ENSMUSG00000043004|ENSMUSG00000052889
Environmental Information Processing	Signal transduction	MAPK signaling pathway	mmu04010	22	254	0.000207359	ENSMUSG00000059970|ENSMUSG00000031309|ENSMUSG00000022523|ENSMUSG00000068798|ENSMUSG00000023050|ENSMUSG00000057967|ENSMUSG00000024235|ENSMUSG00000023034|ENSMUSG00000015968|ENSMUSG00000004113|ENSMUSG00000015312|ENSMUSG00000024383|ENSMUSG00000021936|ENSMUSG00000038387|ENSMUSG00000033220|ENSMUSG00000027164|ENSMUSG00000007613|ENSMUSG00000022329|ENSMUSG00000018932|ENSMUSG00000020882|ENSMUSG00000052889|ENSMUSG00000026072
Environmental	Signal	PI3K-Akt	mmu0	26	350	0.00054	ENSMUSG00000040329|ENSMUSG00000103735|ENSMUSG0000002

Information Processing	transduction	signaling pathway	4151			1232	2523|ENSMUSG00000025017|ENSMUSG00000028518|ENSMUSG00000023034|ENSMUSG00000022309|ENSMUSG00000027009|ENSMUSG00000045613|ENSMUSG00000057967|ENSMUSG00000020048|ENSMUSG00000019966|ENSMUSG00000094694|ENSMUSG00000038668|ENSMUSG00000025915|ENSMUSG00000079465|ENSMUSG00000063594|ENSMUSG00000003865|ENSMUSG00000039005|ENSMUSG00000006235|ENSMUSG00000021270|ENSMUSG00000023439|ENSMUSG00000053007|ENSMUSG00000027995|ENSMUSG00000028859|ENSMUSG00000043004
Human Diseases	Infectious diseases: Bacterial	Pertussis	mmu05133	10	74	0.000550563	ENSMUSG00000025888|ENSMUSG00000000290|ENSMUSG00000024164|ENSMUSG00000027164|ENSMUSG00000062929|ENSMUSG00000026874|ENSMUSG00000036887|ENSMUSG00000021936|ENSMUSG00000036896|ENSMUSG00000039005
Organismal Systems	Immune system	Fc gamma R-mediated phagocytosis	mmu04666	11	90	0.000638949	ENSMUSG00000029684|ENSMUSG00000057342|ENSMUSG00000033220|ENSMUSG00000026656|ENSMUSG00000034116|ENSMUSG00000003283|ENSMUSG00000094694|ENSMUSG00000062929|ENSMUSG00000052889|ENSMUSG00000020143|ENSMUSG00000026395
Organismal Systems	Immune system	Natural killer cell mediated cytotoxicity	mmu04650	13	121	0.000658047	ENSMUSG00000004709|ENSMUSG00000030830|ENSMUSG00000034116|ENSMUSG00000000290|ENSMUSG00000004266|ENSMUSG00000059089|ENSMUSG00000015355|ENSMUSG00000015437|ENSMUSG00000033220|ENSMUSG00000030579|ENSMUSG00000039304|ENSMUSG00000094694|ENSMUSG00000052889
Environmental Information Processing	Signal transduction	Hippo signaling pathway	mmu04390	15	154	0.000675907	ENSMUSG00000020782|ENSMUSG00000007613|ENSMUSG00000067336|ENSMUSG00000037411|ENSMUSG00000000290|ENSMUSG00000014956|ENSMUSG00000015957|ENSMUSG00000021796|ENSMUSG00000020644|ENSMUSG00000020135|ENSMUSG00000036904|ENSMUSG00000022568|ENSMUSG00000006262|ENSMUSG00000022329|ENSMUSG00000021112
Human Diseases	Cancers: Overview	Proteoglycans in cancer	mmu05205	18	208	0.00075847	ENSMUSG00000036446|ENSMUSG00000038387|ENSMUSG00000035273|ENSMUSG00000103735|ENSMUSG00000004266|ENSMUSG00000020592|ENSMUSG00000027995|ENSMUSG00000027646|ENSMU

							SG00000062209|ENSMUSG00000014956|ENSMUSG00000015957|ENSMUSG00000022516|ENSMUSG00000024975|ENSMUSG00000022831|ENSMUSG00000019907|ENSMUSG00000036904|ENSMUSG00000052889|ENSMUSG00000039005
Organismal Systems	Immune system	Cytosolic DNA-sensing pathway	mmu04623	9	64	0.000796977	ENSMUSG00000025888|ENSMUSG00000027514|ENSMUSG00000027427|ENSMUSG00000035834|ENSMUSG00000042349|ENSMUSG00000037523|ENSMUSG00000030595|ENSMUSG00000037860|ENSMUSG00000024810
Human Diseases	Endocrine and metabolic diseases	Non-alcoholic fatty liver disease (NAFLD)	mmu04932	15	157	0.000810669	ENSMUSG00000079941|ENSMUSG00000025651|ENSMUSG00000020163|ENSMUSG00000057722|ENSMUSG00000084093|ENSMUSG00000006542|ENSMUSG00000025204|ENSMUSG00000051811|ENSMUSG00000061518|ENSMUSG00000005373|ENSMUSG00000074218|ENSMUSG00000044894|ENSMUSG00000102070|ENSMUSG00000021936|ENSMUSG00000028518
Metabolism	Global and overview maps	Fatty acid metabolism	mmu01212	8	52	0.000914845	ENSMUSG00000027195|ENSMUSG00000010651|ENSMUSG00000031278|ENSMUSG00000029545|ENSMUSG00000021364|ENSMUSG00000022853|ENSMUSG00000050195|ENSMUSG00000020333
Human Diseases	Neurodegenerative diseases	Alzheimer's disease	mmu05010	16	177	0.000953045	ENSMUSG00000079941|ENSMUSG00000025651|ENSMUSG00000020163|ENSMUSG00000078592|ENSMUSG00000084093|ENSMUSG00000015968|ENSMUSG00000078636|ENSMUSG00000025204|ENSMUSG00000051811|ENSMUSG00000061518|ENSMUSG00000074218|ENSMUSG00000016252|ENSMUSG00000102070|ENSMUSG00000031878|ENSMUSG00000064356|ENSMUSG00000044894
Human Diseases	Endocrine and metabolic diseases	Insulin resistance	mmu04931	12	111	0.000995055	ENSMUSG00000003865|ENSMUSG00000005373|ENSMUSG00000031808|ENSMUSG00000027359|ENSMUSG00000006542|ENSMUSG00000014956|ENSMUSG00000031309|ENSMUSG00000028518|ENSMUSG00000053007|ENSMUSG00000021936|ENSMUSG00000029167|ENSMUSG00000052889
Environmental Information Processing	Signal transduction	Jak-STAT signaling pathway	mmu04630	15	161	0.001025216	ENSMUSG00000020027|ENSMUSG00000032578|ENSMUSG00000057722|ENSMUSG00000006235|ENSMUSG00000004266|ENSMUSG00000004043|ENSMUSG00000024539|ENSMUSG00000017057|ENSMU

							SG00000002325|ENSMUSG00000040329|ENSMUSG00000050222|ENSMUSG00000026104|ENSMUSG00000071713|ENSMUSG00000028859|ENSMUSG00000071714
Organismal Systems	Circulatory system	Vascular smooth muscle contraction	mmu04270	13	128	0.001059447	ENSMUSG00000059588|ENSMUSG00000027999|ENSMUSG00000041193|ENSMUSG00000015968|ENSMUSG00000019907|ENSMUSG00000014956|ENSMUSG00000044951|ENSMUSG00000052920|ENSMUSG00000041202|ENSMUSG00000027335|ENSMUSG00000027523|ENSMUSG00000037166|ENSMUSG00000052889
Environmental Information Processing	Signal transduction	Calcium signaling pathway	mmu04020	16	182	0.001247758	ENSMUSG00000062209|ENSMUSG00000028036|ENSMUSG00000054640|ENSMUSG00000004347|ENSMUSG00000057342|ENSMUSG00000053004|ENSMUSG00000045613|ENSMUSG00000094694|ENSMUSG00000027296|ENSMUSG00000015968|ENSMUSG00000027335|ENSMUSG00000044951|ENSMUSG00000027523|ENSMUSG00000004113|ENSMUSG00000052889|ENSMUSG00000107747
Cellular Processes	Transport and catabolism	Endocytosis	mmu04144	22	294	0.001275698	ENSMUSG00000067235|ENSMUSG00000031488|ENSMUSG00000059970|ENSMUSG00000027423|ENSMUSG00000040990|ENSMUSG00000112639|ENSMUSG00000030465|ENSMUSG00000020817|ENSMUSG00000007613|ENSMUSG00000027646|ENSMUSG00000062209|ENSMUSG00000029684|ENSMUSG00000055413|ENSMUSG00000022797|ENSMUSG00000036499|ENSMUSG00000005656|ENSMUSG00000023353|ENSMUSG00000027164|ENSMUSG00000020078|ENSMUSG00000049076|ENSMUSG00000010080|ENSMUSG00000015733
Organismal Systems	Immune system	RIG-I-like receptor signaling pathway	mmu04622	9	69	0.001288109	ENSMUSG00000032109|ENSMUSG00000042349|ENSMUSG00000064289|ENSMUSG00000037523|ENSMUSG00000027164|ENSMUSG00000030595|ENSMUSG00000021936|ENSMUSG00000026896|ENSMUSG00000039285
Metabolism	Energy metabolism	Sulfur metabolism	mmu00920	4	11	0.001330624	ENSMUSG00000064254|ENSMUSG00000049858|ENSMUSG00000071711|ENSMUSG00000044986
Human Diseases	Cardiovascular diseases	Hypertrophic cardiomyop	mmu05410	10	84	0.001330869	ENSMUSG00000053093|ENSMUSG00000103735|ENSMUSG00000027009|ENSMUSG00000006542|ENSMUSG00000020354|ENSMUSG00000001508|ENSMUSG00000028518|ENSMUSG00000015968|ENSMU

		athy (HCM)					SG00000054640|ENSMUSG00000020882
Metabolism	Amino acid metabolism	Arginine biosynthesis	mmu00220	5	20	0.001341055	ENSMUSG00000025991|ENSMUSG00000022546|ENSMUSG00000019987|ENSMUSG00000023262|ENSMUSG00000031173
Human Diseases	Immune diseases	Systemic lupus erythematosus	mmu05322	14	149	0.001379975	ENSMUSG00000060981|ENSMUSG00000096010|ENSMUSG00000022181|ENSMUSG00000024164|ENSMUSG00000018199|ENSMUSG00000059089|ENSMUSG00000006457|ENSMUSG00000016559|ENSMUSG00000026874|ENSMUSG00000061482|ENSMUSG00000094694|ENSMUSG00000036887|ENSMUSG00000049932|ENSMUSG00000036896
Metabolism	Carbohydrate metabolism	Propanoate metabolism	mmu00640	6	31	0.001418154	ENSMUSG00000074064|ENSMUSG00000022853|ENSMUSG00000057880|ENSMUSG00000032263|ENSMUSG00000032527|ENSMUSG00000060376
Metabolism	Global and overview maps	Carbon metabolism	mmu01200	12	118	0.001608018	ENSMUSG00000025991|ENSMUSG00000022546|ENSMUSG00000023070|ENSMUSG00000078592|ENSMUSG00000029545|ENSMUSG00000078636|ENSMUSG00000026005|ENSMUSG00000024892|ENSMUSG00000022853|ENSMUSG00000021913|ENSMUSG00000032527|ENSMUSG00000011752
Organismal Systems	Nervous system	Dopaminergic synapse	mmu04728	13	135	0.001647553	ENSMUSG00000026824|ENSMUSG00000043004|ENSMUSG00000023439|ENSMUSG00000053007|ENSMUSG00000020182|ENSMUSG00000014956|ENSMUSG00000015968|ENSMUSG00000027523|ENSMUSG00000055116|ENSMUSG00000021936|ENSMUSG00000004113|ENSMUSG00000052889|ENSMUSG00000063594
Organismal Systems	Immune system	Hematopoietic cell lineage	mmu04640	10	87	0.001690487	ENSMUSG00000040329|ENSMUSG00000006235|ENSMUSG00000027009|ENSMUSG00000004609|ENSMUSG00000026399|ENSMUSG00000022797|ENSMUSG00000094694|ENSMUSG00000028859|ENSMUSG00000026072|ENSMUSG00000019966
Human Diseases	Infectious diseases: Parasitic	Chagas disease (American trypanosomiasis)	mmu05142	11	104	0.001865127	ENSMUSG00000036887|ENSMUSG00000024164|ENSMUSG00000027995|ENSMUSG00000027164|ENSMUSG00000007613|ENSMUSG00000027523|ENSMUSG00000035352|ENSMUSG00000021936|ENSMUSG00000037411|ENSMUSG00000036896|ENSMUSG00000039005

<table>
<tr><td>Metabolism</td><td>Metabolism of other amino acids</td><td>beta-Alanine metabolism</td><td>mmu00410</td><td>6</td><td>33</td><td>0.001879043</td><td>ENSMUSG00000074064|ENSMUSG00000024885|ENSMUSG00000022853|ENSMUSG00000057880|ENSMUSG00000029455|ENSMUSG00000006442</td></tr>
<tr><td>Organismal Systems</td><td>Immune system</td><td>NOD-like receptor signaling pathway</td><td>mmu04621</td><td>8</td><td>59</td><td>0.001894237</td><td>ENSMUSG00000025888|ENSMUSG00000055994|ENSMUSG00000021270|ENSMUSG00000027164|ENSMUSG00000020048|ENSMUSG00000030595|ENSMUSG00000035352|ENSMUSG00000021936</td></tr>
<tr><td>Human Diseases</td><td>Cardiovascular diseases</td><td>Arrhythmogenic right ventricular cardiomyopathy (ARVC)</td><td>mmu05412</td><td>9</td><td>74</td><td>0.001998248</td><td>ENSMUSG00000103735|ENSMUSG00000027009|ENSMUSG00000020354|ENSMUSG00000001552|ENSMUSG00000001508|ENSMUSG00000015968|ENSMUSG00000006457|ENSMUSG00000054640|ENSMUSG00000020882</td></tr>
<tr><td>Cellular Processes</td><td>Cell growth and death</td><td>Apoptosis</td><td>mmu04210</td><td>13</td><td>139</td><td>0.002090405</td><td>ENSMUSG00000071713|ENSMUSG00000025860|ENSMUSG00000015437|ENSMUSG00000020486|ENSMUSG00000015312|ENSMUSG00000071714|ENSMUSG00000038642|ENSMUSG00000021936|ENSMUSG00000026202|ENSMUSG00000083282|ENSMUSG00000015337|ENSMUSG00000030560|ENSMUSG00000039304</td></tr>
<tr><td>Metabolism</td><td>Lipid metabolism</td><td>Arachidonic acid metabolism</td><td>mmu00590</td><td>10</td><td>90</td><td>0.002124956</td><td>ENSMUSG00000071072|ENSMUSG00000083816|ENSMUSG00000003053|ENSMUSG00000027999|ENSMUSG00000041193|ENSMUSG00000018924|ENSMUSG00000028597|ENSMUSG00000041202|ENSMUSG00000050737|ENSMUSG00000022947</td></tr>
<tr><td>Human Diseases</td><td>Cardiovascular diseases</td><td>Dilated cardiomyopathy</td><td>mmu05414</td><td>10</td><td>90</td><td>0.002124956</td><td>ENSMUSG00000053093|ENSMUSG00000103735|ENSMUSG00000027009|ENSMUSG00000015968|ENSMUSG00000020354|ENSMUSG00000001508|ENSMUSG00000094694|ENSMUSG00000054640|ENSMUSG00000027523|ENSMUSG00000020882</td></tr>
<tr><td>Organismal Systems</td><td>Endocrine system</td><td>Oxytocin signaling pathway</td><td>mmu04921</td><td>14</td><td>158</td><td>0.002285495</td><td>ENSMUSG00000026824|ENSMUSG00000083816|ENSMUSG00000039145|ENSMUSG00000006542|ENSMUSG00000027646|ENSMUSG00000009292|ENSMUSG00000014956|ENSMUSG00000019907|ENSMUSG00000028518|ENSMUSG00000044951|ENSMUSG00000027523|ENSMUSG00000020882|ENSMUSG00000015968|ENSMUSG00000052889</td></tr>
</table>

Environmental Information Processing	Signal transduction	Rap1 signaling pathway	mmu04015	17	215	0.002551704	ENSMUSG00000022309|ENSMUSG00000038387|ENSMUSG00000000290|ENSMUSG00000000600|ENSMUSG00000030830|ENSMUSG00000057967|ENSMUSG00000026786|ENSMUSG00000038668|ENSMUSG00000027646|ENSMUSG00000033220|ENSMUSG00000022523|ENSMUSG00000037533|ENSMUSG00000068798|ENSMUSG00000019966|ENSMUSG00000027523|ENSMUSG00000018932|ENSMUSG00000052889
Metabolism	Amino acid metabolism	Cysteine and methionine metabolism	mmu00270	7	49	0.002759149	ENSMUSG00000001670|ENSMUSG00000028124|ENSMUSG00000037798|ENSMUSG00000030826|ENSMUSG00000006442|ENSMUSG00000071711|ENSMUSG00000044986
Metabolism	Lipid metabolism	Fatty acid degradation	mmu00071	7	49	0.002759149	ENSMUSG00000010651|ENSMUSG00000031278|ENSMUSG00000003809|ENSMUSG00000029545|ENSMUSG00000022853|ENSMUSG00000029455|ENSMUSG00000020333
Environmental Information Processing	Signal transduction	HIF-1 signaling pathway	mmu04066	11	110	0.002788061	ENSMUSG00000022309|ENSMUSG00000078592|ENSMUSG00000037411|ENSMUSG00000078636|ENSMUSG00000015340|ENSMUSG00000021367|ENSMUSG00000033688|ENSMUSG00000022797|ENSMUSG00000039005|ENSMUSG00000052889|ENSMUSG00000026773
Human Diseases	Cancers: Overview	Transcriptional misregulation in cancer	mmu05202	15	180	0.002809436	ENSMUSG00000094694|ENSMUSG00000002111|ENSMUSG00000055435|ENSMUSG00000035356|ENSMUSG00000028926|ENSMUSG00000015437|ENSMUSG00000001552|ENSMUSG00000016559|ENSMUSG00000037369|ENSMUSG00000056501|ENSMUSG00000020644|ENSMUSG00000031613|ENSMUSG00000006586|ENSMUSG00000025142|ENSMUSG00000037876
Human Diseases	Neurodegenerative diseases	Huntington's disease	mmu05016	16	199	0.002884318	ENSMUSG00000079941|ENSMUSG00000025651|ENSMUSG00000020163|ENSMUSG00000084093|ENSMUSG00000053007|ENSMUSG00000034706|ENSMUSG00000025204|ENSMUSG00000016252|ENSMUSG00000061518|ENSMUSG00000074218|ENSMUSG00000051811|ENSMUSG00000102070|ENSMUSG00000030922|ENSMUSG00000029167|ENSMUSG00000064356|ENSMUSG00000044894
Human	Infectious	Malaria	mmu0	7	50	0.00305	ENSMUSG00000030830|ENSMUSG00000069917|ENSMUSG0000000

Diseases	diseases: Parasitic		5144			4983	0290|ENSMUSG00000027995|ENSMUSG00000035352|ENSMUSG00000020592|ENSMUSG00000039005
Environmental Information Processing	Signal transduction	Wnt signaling pathway	mmu04310	13	146	0.003099006	ENSMUSG00000042988|ENSMUSG00000040260|ENSMUSG00000015957|ENSMUSG00000033220|ENSMUSG00000027996|ENSMUSG00000031548|ENSMUSG00000081219|ENSMUSG00000020218|ENSMUSG00000020135|ENSMUSG00000014226|ENSMUSG00000021936|ENSMUSG00000036904|ENSMUSG00000052889
Human Diseases	Cardiovascular diseases	Viral myocarditis	mmu05416	10	96	0.00326231	ENSMUSG00000067235|ENSMUSG00000030830|ENSMUSG00000053093|ENSMUSG00000000290|ENSMUSG00000033220|ENSMUSG00000020354|ENSMUSG00000026399|ENSMUSG00000001508|ENSMUSG00000094694|ENSMUSG00000055413
Metabolism	Glycan biosynthesis and metabolism	Glycosaminoglycan biosynthesis - keratan sulfate	mmu00533	4	15	0.003391464	ENSMUSG00000028541|ENSMUSG00000031749|ENSMUSG00000028413|ENSMUSG00000047379
Metabolism	Carbohydrate metabolism	Butanoate metabolism	mmu00650	5	27	0.004178751	ENSMUSG00000046598|ENSMUSG00000057880|ENSMUSG00000029545|ENSMUSG00000022853|ENSMUSG00000029482
Metabolism	Amino acid metabolism	Tyrosine metabolism	mmu00350	6	40	0.004414285	ENSMUSG00000022821|ENSMUSG00000001670|ENSMUSG00000030630|ENSMUSG00000024885|ENSMUSG00000020182|ENSMUSG00000021033
Organismal Systems	Digestive system	Fat digestion and absorption	mmu04975	6	40	0.004414285	ENSMUSG00000027999|ENSMUSG00000041193|ENSMUSG00000041202|ENSMUSG00000020609|ENSMUSG00000032083|ENSMUSG00000030747
Genetic Information Processing	Transcription	Spliceosome	mmu03040	12	135	0.004444676	ENSMUSG00000074088|ENSMUSG00000010608|ENSMUSG00000031134|ENSMUSG00000059970|ENSMUSG00000039630|ENSMUSG00000061136|ENSMUSG00000008333|ENSMUSG00000032407|ENSMUSG00000037475|ENSMUSG00000025024|ENSMUSG00000031157|ENSMUSG00000001158
Organismal	Immune	Toll-like	mmu0	10	101	0.00454	ENSMUSG00000026104|ENSMUSG00000027995|ENSMUSG0000004

Systems	system	receptor signaling pathway	4620			0471	4827|ENSMUSG00000027164|ENSMUSG00000024235|ENSMUSG00000044583|ENSMUSG00000042349|ENSMUSG00000018932|ENSMUSG00000021936|ENSMUSG00000039005
Metabolism	Lipid metabolism	Biosynthesis of unsaturated fatty acids	mmu01040	5	28	0.004789571	ENSMUSG00000050195|ENSMUSG00000021364|ENSMUSG00000072949|ENSMUSG00000027195|ENSMUSG00000010651
Human Diseases	Infectious diseases: Viral	Hepatitis C	mmu05160	12	137	0.00494911	ENSMUSG00000079339|ENSMUSG00000032661|ENSMUSG00000041378|ENSMUSG00000002325|ENSMUSG00000024079|ENSMUSG00000042349|ENSMUSG00000037523|ENSMUSG00000027164|ENSMUSG00000022336|ENSMUSG00000026104|ENSMUSG00000021936|ENSMUSG00000001739
Organismal Systems	Digestive system	Pancreatic secretion	mmu04972	10	103	0.005150266	ENSMUSG00000027999|ENSMUSG00000041193|ENSMUSG00000001865|ENSMUSG00000032839|ENSMUSG00000019066|ENSMUSG00000021999|ENSMUSG00000041202|ENSMUSG00000027523|ENSMUSG00000068798|ENSMUSG00000052889
Human Diseases	Endocrine and metabolic diseases	AGE-RAGE signaling pathway in diabetic complications	mmu04933	10	103	0.005150266	ENSMUSG00000007613|ENSMUSG00000004043|ENSMUSG00000037411|ENSMUSG00000015340|ENSMUSG00000021367|ENSMUSG00000026104|ENSMUSG00000035352|ENSMUSG00000021936|ENSMUSG00000052889|ENSMUSG00000079465
Organismal Systems	Immune system	Leukocyte transendothelial migration	mmu04670	11	121	0.005412665	ENSMUSG00000041378|ENSMUSG00000030830|ENSMUSG00000034116|ENSMUSG00000000290|ENSMUSG00000027009|ENSMUSG00000015340|ENSMUSG00000033220|ENSMUSG00000068798|ENSMUSG00000006457|ENSMUSG00000052889|ENSMUSG00000001739
Metabolism	Metabolism of other amino acids	Glutathione metabolism	mmu00480	7	57	0.005855194	ENSMUSG00000022292|ENSMUSG00000028124|ENSMUSG00000028597|ENSMUSG00000080845|ENSMUSG00000006442|ENSMUSG00000020649|ENSMUSG00000022562
Organismal Systems	Immune system	Platelet activation	mmu04611	11	123	0.006050675	ENSMUSG00000019907|ENSMUSG00000033860|ENSMUSG00000026786|ENSMUSG00000027646|ENSMUSG00000014956|ENSMUSG00

<table>
<tr><td></td><td></td><td></td><td></td><td></td><td></td><td></td><td>000028001|ENSMUSG00000052920|ENSMUSG00000068798|ENSMUSG00000044951|ENSMUSG00000033831|ENSMUSG00000027523</td></tr>
<tr><td>Cellular Processes</td><td>Cellular community - eukaryotes</td><td>Tight junction</td><td>mmu04530</td><td>12</td><td>141</td><td>0.006094513</td><td>ENSMUSG00000041378|ENSMUSG00000038387|ENSMUSG00000030739|ENSMUSG00000027646|ENSMUSG00000053093|ENSMUSG00000074652|ENSMUSG00000006457|ENSMUSG00000022831|ENSMUSG00000052889|ENSMUSG00000020782|ENSMUSG00000001739|ENSMUSG00000021112</td></tr>
<tr><td>Organismal Systems</td><td>Nervous system</td><td>GABAergic synapse</td><td>mmu04727</td><td>9</td><td>89</td><td>0.006112111</td><td>ENSMUSG00000043004|ENSMUSG00000023439|ENSMUSG00000023169|ENSMUSG00000027646|ENSMUSG00000057880|ENSMUSG00000015968|ENSMUSG00000004113|ENSMUSG00000052889|ENSMUSG00000063594</td></tr>
<tr><td>Organismal Systems</td><td>Endocrine system</td><td>Adipocytokine signaling pathway</td><td>mmu04920</td><td>8</td><td>73</td><td>0.006176842</td><td>ENSMUSG00000057722|ENSMUSG00000028518|ENSMUSG00000031278|ENSMUSG00000006542|ENSMUSG00000030595|ENSMUSG00000020333|ENSMUSG00000021936|ENSMUSG00000029167</td></tr>
<tr><td>Human Diseases</td><td>Infectious diseases: Bacterial</td><td>Legionellosis</td><td>mmu05134</td><td>7</td><td>58</td><td>0.006374124</td><td>ENSMUSG00000025888|ENSMUSG00000000290|ENSMUSG00000059970|ENSMUSG00000027995|ENSMUSG00000024164|ENSMUSG00000078566|ENSMUSG00000039005</td></tr>
<tr><td>Metabolism</td><td>Lipid metabolism</td><td>Ether lipid metabolism</td><td>mmu00565</td><td>6</td><td>44</td><td>0.006681141</td><td>ENSMUSG00000027999|ENSMUSG00000052160|ENSMUSG00000041193|ENSMUSG00000040774|ENSMUSG00000041202|ENSMUSG00000038173</td></tr>
<tr><td>Human Diseases</td><td>Immune diseases</td><td>Inflammatory bowel disease (IBD)</td><td>mmu05321</td><td>7</td><td>59</td><td>0.006926512</td><td>ENSMUSG00000055994|ENSMUSG00000055435|ENSMUSG00000027995|ENSMUSG00000032238|ENSMUSG00000026104|ENSMUSG00000001444|ENSMUSG00000039005</td></tr>
<tr><td>Organismal Systems</td><td>Environmental adaptation</td><td>Circadian rhythm</td><td>mmu04710</td><td>5</td><td>31</td><td>0.006998729</td><td>ENSMUSG00000055116|ENSMUSG00000006542|ENSMUSG00000032238|ENSMUSG00000028518|ENSMUSG00000030103</td></tr>
<tr><td>Organismal Systems</td><td>Sensory system</td><td>Inflammatory mediator regulation of TRP channels</td><td>mmu04750</td><td>11</td><td>126</td><td>0.007116783</td><td>ENSMUSG00000003053|ENSMUSG00000027523|ENSMUSG00000053004|ENSMUSG00000032860|ENSMUSG00000027646|ENSMUSG00000033007|ENSMUSG00000021936|ENSMUSG00000018932|ENSMUSG00000014956|ENSMUSG00000052889|ENSMUSG00000026072</td></tr>
</table>

<table>
<tr><td>Genetic Information Processing</td><td>Folding, sorting and degradation</td><td>Ubiquitin mediated proteolysis</td><td>mmu04120</td><td>12</td><td>145</td><td>0.007440157</td><td>ENSMUSG00000020802|ENSMUSG00000025860|ENSMUSG00000095134|ENSMUSG00000025939|ENSMUSG00000030061|ENSMUSG00000020687|ENSMUSG00000024807|ENSMUSG00000112639|ENSMUSG00000027164|ENSMUSG00000021728|ENSMUSG00000025326|ENSMUSG00000070923</td></tr>
<tr><td>Human Diseases</td><td>Infectious diseases: Viral</td><td>Hepatitis B</td><td>mmu05161</td><td>12</td><td>146</td><td>0.007810485</td><td>ENSMUSG00000004043|ENSMUSG00000027995|ENSMUSG00000053007|ENSMUSG00000027646|ENSMUSG00000042349|ENSMUSG00000007613|ENSMUSG00000037523|ENSMUSG00000026104|ENSMUSG00000021936|ENSMUSG00000039005|ENSMUSG00000052889|ENSMUSG00000026896</td></tr>
<tr><td>Human Diseases</td><td>Substance dependence</td><td>Morphine addiction</td><td>mmu05032</td><td>9</td><td>93</td><td>0.007898386</td><td>ENSMUSG00000026824|ENSMUSG00000043004|ENSMUSG00000023439|ENSMUSG00000004347|ENSMUSG00000069094|ENSMUSG00000027523|ENSMUSG00000004113|ENSMUSG00000052889|ENSMUSG00000063594</td></tr>
<tr><td>Organismal Systems</td><td>Digestive system</td><td>Mineral absorption</td><td>mmu04978</td><td>6</td><td>46</td><td>0.00808598</td><td>ENSMUSG00000025993|ENSMUSG00000033688|ENSMUSG00000020829|ENSMUSG00000054640|ENSMUSG00000027365|ENSMUSG00000031765</td></tr>
<tr><td>Environmental Information Processing</td><td>Membrane transport</td><td>ABC transporters</td><td>mmu02010</td><td>6</td><td>46</td><td>0.00808598</td><td>ENSMUSG00000020620|ENSMUSG00000050296|ENSMUSG00000029408|ENSMUSG00000026944|ENSMUSG00000029802|ENSMUSG00000031333</td></tr>
<tr><td>Human Diseases</td><td>Substance dependence</td><td>Alcoholism</td><td>mmu05034</td><td>15</td><td>204</td><td>0.008111404</td><td>ENSMUSG00000049932|ENSMUSG00000060981|ENSMUSG00000023439|ENSMUSG00000096010|ENSMUSG00000027499|ENSMUSG00000053007|ENSMUSG00000020182|ENSMUSG00000034245|ENSMUSG00000014956|ENSMUSG00000016559|ENSMUSG00000061482|ENSMUSG00000004698|ENSMUSG00000027523|ENSMUSG00000043004|ENSMUSG00000063594</td></tr>
<tr><td>Human Diseases</td><td>Infectious diseases: Parasitic</td><td>Amoebiasis</td><td>mmu05146</td><td>10</td><td>111</td><td>0.008254539</td><td>ENSMUSG00000000290|ENSMUSG00000027995|ENSMUSG00000026072|ENSMUSG00000094694|ENSMUSG00000006457|ENSMUSG00000027523|ENSMUSG00000019987|ENSMUSG00000052889|ENSMUSG00000079465|ENSMUSG00000039005</td></tr>
<tr><td>Environmental</td><td>Signal</td><td>AMPK</td><td>mmu0</td><td>11</td><td>129</td><td>0.00832</td><td>ENSMUSG00000003865|ENSMUSG00000074064|ENSMUSG0000005</td></tr>
</table>

Information Processing	transduction	signaling pathway	4152			4082	7722|ENSMUSG00000053007|ENSMUSG00000006542|ENSMUSG00000017950|ENSMUSG00000028518|ENSMUSG00000101904|ENSMUSG00000050195|ENSMUSG00000029167|ENSMUSG00000026773
Metabolism	Lipid metabolism	Glycerophospholipid metabolism	mmu00564	9	94	0.008401365	ENSMUSG00000025495|ENSMUSG00000027999|ENSMUSG00000041193|ENSMUSG00000004270|ENSMUSG00000052160|ENSMUSG00000040774|ENSMUSG00000041202|ENSMUSG00000024978|ENSMUSG00000036833
Human Diseases	Cancers: Overview	Viral carcinogenesis	mmu05203	17	245	0.008493489	ENSMUSG00000067235|ENSMUSG00000060981|ENSMUSG00000096010|ENSMUSG00000024164|ENSMUSG00000002325|ENSMUSG00000004043|ENSMUSG00000053007|ENSMUSG00000024079|ENSMUSG00000027646|ENSMUSG00000034245|ENSMUSG00000006457|ENSMUSG00000055413|ENSMUSG00000022568|ENSMUSG00000004698|ENSMUSG00000025326|ENSMUSG00000001524|ENSMUSG00000061482
Environmental Information Processing	Signal transduction	TNF signaling pathway	mmu04668	10	112	0.008726142	ENSMUSG00000083816|ENSMUSG00000053007|ENSMUSG00000055994|ENSMUSG00000021367|ENSMUSG00000024235|ENSMUSG00000056501|ENSMUSG00000021936|ENSMUSG00000035352|ENSMUSG00000018932|ENSMUSG00000022789
Organismal Systems	Nervous system	Cholinergic synapse	mmu04725	10	113	0.009218118	ENSMUSG00000026824|ENSMUSG00000043004|ENSMUSG00000023439|ENSMUSG00000053007|ENSMUSG00000045613|ENSMUSG00000028631|ENSMUSG00000015968|ENSMUSG00000004113|ENSMUSG00000052889|ENSMUSG00000063594
Human Diseases	Infectious diseases: Parasitic	Toxoplasmosis	mmu05145	10	113	0.009218118	ENSMUSG00000025860|ENSMUSG00000059970|ENSMUSG00000027995|ENSMUSG00000026104|ENSMUSG00000046879|ENSMUSG00000027164|ENSMUSG00000030595|ENSMUSG00000018932|ENSMUSG00000021936|ENSMUSG00000039005
Organismal Systems	Nervous system	Glutamatergic synapse	mmu04724	10	115	0.010265383	ENSMUSG00000026824|ENSMUSG00000023439|ENSMUSG00000023169|ENSMUSG00000032839|ENSMUSG00000007617|ENSMUSG00000015968|ENSMUSG00000027523|ENSMUSG00000043004|ENSMUSG00000052889|ENSMUSG00000063594
Organismal	Environmental	Circadian	mmu0	9	98	0.01065	ENSMUSG00000026824|ENSMUSG00000023439|ENSMUSG0000004

Systems	adaptation	entrainment	4713			9632	9892|ENSMUSG00000052920|ENSMUSG00000015968|ENSMUSG00000027523|ENSMUSG00000043004|ENSMUSG00000052889|ENSMUSG00000063594
Human Diseases	Infectious diseases: Viral	Epstein-Barr virus infection	mmu05169	16	232	0.010998186	ENSMUSG00000067235|ENSMUSG00000030830|ENSMUSG00000055413|ENSMUSG00000002111|ENSMUSG00000027427|ENSMUSG00000035834|ENSMUSG00000024079|ENSMUSG00000028874|ENSMUSG00000027164|ENSMUSG00000051457|ENSMUSG00000059970|ENSMUSG00000112639|ENSMUSG00000094694|ENSMUSG00000030595|ENSMUSG00000018932|ENSMUSG00000021936
Metabolism	Carbohydrate metabolism	Pentose and glucuronate interconversions	mmu00040	5	36	0.012112459	ENSMUSG00000039450|ENSMUSG00000001891|ENSMUSG00000029201|ENSMUSG00000029455|ENSMUSG00000026005
Organismal Systems	Development	Axon guidance	mmu04360	13	176	0.012699041	ENSMUSG00000022231|ENSMUSG00000038387|ENSMUSG00000026121|ENSMUSG00000021451|ENSMUSG00000032839|ENSMUSG00000067336|ENSMUSG00000027646|ENSMUSG00000033220|ENSMUSG00000022883|ENSMUSG00000028780|ENSMUSG00000062929|ENSMUSG00000038777|ENSMUSG00000030084
Environmental Information Processing	Signaling molecules and interaction	Cell adhesion molecules (CAMs)	mmu04514	13	176	0.012699041	ENSMUSG00000067235|ENSMUSG00000030830|ENSMUSG00000000290|ENSMUSG00000027009|ENSMUSG00000027322|ENSMUSG00000051457|ENSMUSG00000062300|ENSMUSG00000041378|ENSMUSG00000055413|ENSMUSG00000000305|ENSMUSG00000020592|ENSMUSG00000001739|ENSMUSG00000026395
Human Diseases	Infectious diseases: Parasitic	African trypanosomiasis	mmu05143	5	37	0.013374683	ENSMUSG00000032083|ENSMUSG00000052889|ENSMUSG00000004929|ENSMUSG00000069917|ENSMUSG00000094694
Organismal Systems	Nervous system	Retrograde endocannabinoid signaling	mmu04723	9	103	0.014088682	ENSMUSG00000026824|ENSMUSG00000083816|ENSMUSG00000043004|ENSMUSG00000023439|ENSMUSG00000015968|ENSMUSG00000021936|ENSMUSG00000004113|ENSMUSG00000052889|ENSMUSG00000063594
Cellular	Cellular	Gap	mmu0	8	86	0.01460	ENSMUSG00000024383|ENSMUSG00000027646|ENSMUSG0000005

Processes	community - eukaryotes	junction	4540			1367	2920|ENSMUSG00000038668|ENSMUSG00000058672|ENSMUSG00000027523|ENSMUSG00000026202|ENSMUSG00000052889
Metabolism	Amino acid metabolism	Lysine degradation	mmu00310	6	53	0.014673966	ENSMUSG00000003809|ENSMUSG00000032374|ENSMUSG00000022853|ENSMUSG00000029455|ENSMUSG00000021913|ENSMUSG00000034807
Metabolism	Lipid metabolism	Steroid hormone biosynthesis	mmu00140	8	87	0.01548515	ENSMUSG00000026675|ENSMUSG00000094806|ENSMUSG00000027195|ENSMUSG00000029233|ENSMUSG00000003053|ENSMUSG00000068086|ENSMUSG00000024087|ENSMUSG00000032315
Metabolism	Lipid metabolism	alpha-Linolenic acid metabolism	mmu00592	4	25	0.015901727	ENSMUSG00000041193|ENSMUSG00000010651|ENSMUSG00000041202|ENSMUSG00000027999
Cellular Processes	Cellular community - eukaryotes	Focal adhesion	mmu04510	14	203	0.01663498	ENSMUSG00000025860|ENSMUSG00000103735|ENSMUSG00000027009|ENSMUSG00000027646|ENSMUSG00000014956|ENSMUSG00000033220|ENSMUSG00000019907|ENSMUSG00000068798|ENSMUSG00000044951|ENSMUSG00000006457|ENSMUSG00000021936|ENSMUSG00000052889|ENSMUSG00000079465|ENSMUSG00000034116
Metabolism	Glycan biosynthesis and metabolism	Glycosphingolipid biosynthesis - lacto and neolacto series	mmu00601	4	26	0.01786708	ENSMUSG00000028541|ENSMUSG00000047379|ENSMUSG00000028413|ENSMUSG00000055978
Metabolism	Amino acid metabolism	Histidine metabolism	mmu00340	4	26	0.01786708	ENSMUSG00000059422|ENSMUSG00000024885|ENSMUSG00000020182|ENSMUSG00000029455
Metabolism	Metabolism of cofactors and vitamins	Porphyrin and chlorophyll metabolism	mmu00860	5	41	0.019302921	ENSMUSG00000003617|ENSMUSG00000029575|ENSMUSG00000108628|ENSMUSG00000040466|ENSMUSG00000028393
Cellular Processes	Cellular community -	Adherens junction	mmu04520	7	74	0.020062656	ENSMUSG00000033220|ENSMUSG00000007613|ENSMUSG00000004266|ENSMUSG00000027646|ENSMUSG00000062300|ENSMUSG00

	eukaryotes						000006457|ENSMUSG00000029684
Environmental Information Processing	Signal transduction	Ras signaling pathway	mmu04014	15	231	0.02137392	ENSMUSG00000022309|ENSMUSG00000038387|ENSMUSG00000027999|ENSMUSG00000023439|ENSMUSG00000041193|ENSMUSG00000063594|ENSMUSG00000057967|ENSMUSG00000033220|ENSMUSG00000022523|ENSMUSG00000068798|ENSMUSG00000019966|ENSMUSG00000021936|ENSMUSG00000043004|ENSMUSG00000052889|ENSMUSG00000041202
Human Diseases	Infectious diseases: Viral	HTLV-I infection	mmu05166	18	295	0.021421118	ENSMUSG00000028211|ENSMUSG00000007613|ENSMUSG00000067235|ENSMUSG00000030830|ENSMUSG00000038387|ENSMUSG00000015957|ENSMUSG00000004043|ENSMUSG00000002111|ENSMUSG00000020471|ENSMUSG00000020687|ENSMUSG00000020135|ENSMUSG00000055413|ENSMUSG00000000290|ENSMUSG00000063889|ENSMUSG00000021936|ENSMUSG00000036904|ENSMUSG00000026072|ENSMUSG00000025860
Organismal Systems	Circulatory system	Adrenergic signaling in cardiomyocytes	mmu04261	11	152	0.023551232	ENSMUSG00000039639|ENSMUSG00000053093|ENSMUSG00000053007|ENSMUSG00000015968|ENSMUSG00000014956|ENSMUSG00000027335|ENSMUSG00000054640|ENSMUSG00000063889|ENSMUSG00000027523|ENSMUSG00000020882|ENSMUSG00000034810
Environmental Information Processing	Signal transduction	FoxO signaling pathway	mmu04068	10	135	0.026299306	ENSMUSG00000006542|ENSMUSG00000078566|ENSMUSG00000007613|ENSMUSG00000007617|ENSMUSG00000028518|ENSMUSG00000055148|ENSMUSG00000021936|ENSMUSG00000025915|ENSMUSG00000015312|ENSMUSG00000039304
Environmental Information Processing	Signal transduction	cAMP signaling pathway	mmu04024	13	198	0.02850703	ENSMUSG00000038387|ENSMUSG00000033220|ENSMUSG00000053007|ENSMUSG00000015968|ENSMUSG00000045613|ENSMUSG00000014956|ENSMUSG00000051314|ENSMUSG00000019907|ENSMUSG00000068798|ENSMUSG00000027523|ENSMUSG00000021936|ENSMUSG00000020331|ENSMUSG00000034116
Metabolism	Nucleotide metabolism	Purine metabolism	mmu00230	12	179	0.030256949	ENSMUSG00000022292|ENSMUSG00000004347|ENSMUSG00000029780|ENSMUSG00000032478|ENSMUSG00000035834|ENSMUSG00000058624|ENSMUSG00000069094|ENSMUSG00000027427|ENSMUSG00000020471|ENSMUSG00000015085|ENSMUSG00000003500|E

							NSMUSG00000020649
Metabolism	Carbohydrate metabolism	Galactose metabolism	mmu00052	4	32	0.032770349	ENSMUSG00000028541|ENSMUSG00000028413|ENSMUSG00000025579|ENSMUSG00000001891
Cellular Processes	Transport and catabolism	Peroxisome	mmu04146	7	83	0.033324465	ENSMUSG00000010651|ENSMUSG00000031278|ENSMUSG00000027222|ENSMUSG00000022853|ENSMUSG00000074064|ENSMUSG00000020333|ENSMUSG00000027359
Organismal Systems	Endocrine system	Glucagon signaling pathway	mmu04922	8	102	0.033754869	ENSMUSG00000003865|ENSMUSG00000053007|ENSMUSG00000006542|ENSMUSG00000020463|ENSMUSG00000028518|ENSMUSG00000027523|ENSMUSG00000011752|ENSMUSG00000029167
Human Diseases	Immune diseases	Rheumatoid arthritis	mmu05323	7	85	0.036910873	ENSMUSG00000030830|ENSMUSG00000000290|ENSMUSG00000027995|ENSMUSG00000094694|ENSMUSG00000035352|ENSMUSG00000022309|ENSMUSG00000039005
Environmental Information Processing	Signal transduction	NF-kappa B signaling pathway	mmu04064	8	104	0.0369748	ENSMUSG00000083816|ENSMUSG00000025860|ENSMUSG00000027164|ENSMUSG00000094694|ENSMUSG00000052889|ENSMUSG00000026072|ENSMUSG00000015312|ENSMUSG00000039005
Metabolism	Nucleotide metabolism	Pyrimidine metabolism	mmu00240	8	104	0.0369748	ENSMUSG00000022292|ENSMUSG00000029780|ENSMUSG00000032478|ENSMUSG00000035834|ENSMUSG00000042462|ENSMUSG00000027427|ENSMUSG00000020471|ENSMUSG00000020649
Cellular Processes	Transport and catabolism	Lysosome	mmu04142	9	124	0.037642429	ENSMUSG00000024480|ENSMUSG00000029408|ENSMUSG00000026944|ENSMUSG00000028581|ENSMUSG00000038642|ENSMUSG00000025579|ENSMUSG00000083282|ENSMUSG00000004567|ENSMUSG00000030560
Metabolism	Lipid metabolism	Linoleic acid metabolism	mmu00591	5	50	0.038296454	ENSMUSG00000018924|ENSMUSG00000041193|ENSMUSG00000003053|ENSMUSG00000041202|ENSMUSG00000027999
Human Diseases	Neurodegenerative diseases	Prion diseases	mmu05020	4	34	0.038961806	ENSMUSG00000036887|ENSMUSG00000026874|ENSMUSG00000036896|ENSMUSG00000022181
Human Diseases	Substance dependence	Amphetamine addiction	mmu05031	6	68	0.039587292	ENSMUSG00000053007|ENSMUSG00000020182|ENSMUSG00000014956|ENSMUSG00000015968|ENSMUSG00000027523|ENSMUSG00000052889
Metabolism	Global and overview maps	2-Oxocarboxylic acid	mmu01210	3	20	0.041554138	ENSMUSG00000022546|ENSMUSG00000023262|ENSMUSG00000030826

		metabolism					
Organismal Systems	Endocrine system	GnRH signaling pathway	mmu04912	7	88	0.042751432	ENSMUSG00000027523|ENSMUSG00000015968|ENSMUSG00000024383|ENSMUSG00000027646|ENSMUSG00000021936|ENSMUSG00000018932|ENSMUSG00000052889
Genetic Information Processing	Translation	RNA transport	mmu03013	11	170	0.045157208	ENSMUSG00000052825|ENSMUSG00000000838|ENSMUSG00000022336|ENSMUSG00000037475|ENSMUSG00000027236|ENSMUSG00000013736|ENSMUSG00000027680|ENSMUSG00000053453|ENSMUSG00000057561|ENSMUSG00000021282|ENSMUSG00000029145
Organismal Systems	Endocrine system	Renin-angiotensin system	mmu04614	4	36	0.04577466	ENSMUSG00000023845|ENSMUSG00000001865|ENSMUSG00000004929|ENSMUSG00000068037
Organismal Systems	Endocrine system	Prolactin signaling pathway	mmu04917	6	74	0.054343287	ENSMUSG00000020027|ENSMUSG00000032578|ENSMUSG00000004043|ENSMUSG00000027646|ENSMUSG00000026104|ENSMUSG00000021936
Metabolism	Carbohydrate metabolism	Pyruvate metabolism	mmu00620	4	39	0.057154571	ENSMUSG00000031958|ENSMUSG00000024892|ENSMUSG00000024158|ENSMUSG00000029455
Human Diseases	Cancers: Specific types	Acute myeloid leukemia	mmu05221	5	57	0.058923413	ENSMUSG00000004043|ENSMUSG00000031155|ENSMUSG00000006586|ENSMUSG00000001552|ENSMUSG00000002111
Genetic Information Processing	Folding, sorting and degradation	Sulfur relay system	mmu04122	2	10	0.060519635	ENSMUSG00000071711|ENSMUSG00000044986
Metabolism	Amino acid metabolism	Phenylalanine metabolism	mmu00360	3	24	0.062178788	ENSMUSG00000024885|ENSMUSG00000001670|ENSMUSG00000020182
Organismal Systems	Digestive system	Vitamin digestion and absorption	mmu04977	3	24	0.062178788	ENSMUSG00000032083|ENSMUSG00000020609|ENSMUSG00000020829
Organismal Systems	Endocrine system	Ovarian steroidogen	mmu04913	5	58	0.062299091	ENSMUSG00000026675|ENSMUSG00000027523|ENSMUSG00000032315|ENSMUSG00000024087|ENSMUSG00000083816

		esis					
Metabolism	Lipid metabolism	Glycerolipid metabolism	mmu00561	5	59	0.065781681	ENSMUSG00000025059|ENSMUSG00000025509|ENSMUSG00000024978|ENSMUSG00000029455|ENSMUSG00000030747
Organismal Systems	Endocrine system	Estrogen signaling pathway	mmu04915	7	98	0.066367242	ENSMUSG00000026824|ENSMUSG00000059970|ENSMUSG00000053007|ENSMUSG00000021270|ENSMUSG00000027646|ENSMUSG00000020048|ENSMUSG00000027523
Environmental Information Processing	Signal transduction	VEGF signaling pathway	mmu04370	5	60	0.069370761	ENSMUSG00000033220|ENSMUSG00000083816|ENSMUSG00000052889|ENSMUSG00000027646|ENSMUSG00000057342
Metabolism	Metabolism of cofactors and vitamins	Ubiquinone and other terpenoid-quinone biosynthesis	mmu00130	2	11	0.069956132	ENSMUSG00000001670|ENSMUSG00000021235
Genetic Information Processing	Replication and repair	Nucleotide excision repair	mmu03420	4	44	0.079146468	ENSMUSG00000069089|ENSMUSG00000020471|ENSMUSG00000031347|ENSMUSG00000001524
Metabolism	Lipid metabolism	Fatty acid elongation	mmu00062	3	27	0.080188719	ENSMUSG00000021364|ENSMUSG00000072949|ENSMUSG00000027195
Metabolism	Carbohydrate metabolism	Ascorbate and aldarate metabolism	mmu00053	3	27	0.080188719	ENSMUSG00000029201|ENSMUSG00000023070|ENSMUSG00000029455
Genetic Information Processing	Folding, sorting and degradation	RNA degradation	mmu03018	6	83	0.081960628	ENSMUSG00000034724|ENSMUSG00000027714|ENSMUSG00000027770|ENSMUSG00000000581|ENSMUSG00000033991|ENSMUSG00000036779
Human Diseases	Cancers: Specific types	Colorectal cancer	mmu05210	5	64	0.084779561	ENSMUSG00000033220|ENSMUSG00000021936|ENSMUSG00000020135|ENSMUSG00000040760|ENSMUSG00000007613
Metabolism	Glycan biosynthesis and metabolism	Mucin type O-Glycan biosynthesis	mmu00512	3	28	0.086639982	ENSMUSG00000031749|ENSMUSG00000042460|ENSMUSG00000038843

Metabolism	Xenobiotics biodegradation and metabolism	Metabolism of xenobiotics by cytochrome P450	mmu00980	5	65	0.088890804	ENSMUSG00000028743|ENSMUSG00000024885|ENSMUSG00000032315|ENSMUSG00000024087|ENSMUSG00000022947
Environmental Information Processing	Signal transduction	TGF-beta signaling pathway	mmu04350	6	85	0.088988566	ENSMUSG00000021796|ENSMUSG00000067336|ENSMUSG00000081219|ENSMUSG00000007613|ENSMUSG00000020644|ENSMUSG00000037035
Genetic Information Processing	Replication and repair	Non-homologous end-joining	mmu03450	2	13	0.090109019	ENSMUSG00000026648|ENSMUSG00000021615
Organismal Systems	Endocrine system	Insulin secretion	mmu04911	6	86	0.092621894	ENSMUSG00000053007|ENSMUSG00000002908|ENSMUSG00000031840|ENSMUSG00000015968|ENSMUSG00000027523|ENSMUSG00000052889
Organismal Systems	Endocrine system	Aldosterone synthesis and secretion	mmu04925	6	86	0.092621894	ENSMUSG00000053007|ENSMUSG00000039145|ENSMUSG00000023034|ENSMUSG00000015968|ENSMUSG00000027523|ENSMUSG00000052889
Metabolism	Carbohydrate metabolism	Glycolysis / Gluconeogenesis	mmu00010	5	66	0.093103666	ENSMUSG00000024885|ENSMUSG00000011752|ENSMUSG00000078636|ENSMUSG00000029455|ENSMUSG00000078592
Metabolism	Lipid metabolism	Fatty acid biosynthesis	mmu00061	2	14	0.100746367	ENSMUSG00000020333|ENSMUSG00000031278
Metabolism	Carbohydrate metabolism	Amino sugar and nucleotide sugar metabolism	mmu00520	4	49	0.104715764	ENSMUSG00000001891|ENSMUSG00000042684|ENSMUSG00000022474|ENSMUSG00000029201
Organismal Systems	Immune system	Fc epsilon RI signaling	mmu04664	5	69	0.106339757	ENSMUSG00000033220|ENSMUSG00000021936|ENSMUSG00000034116|ENSMUSG00000094694|ENSMUSG00000018932

		pathway					
Metabolism	Glycan biosynthesis and metabolism	Other types of O-glycan biosynthesis	mmu00514	3	31	0.107230761	ENSMUSG00000028541|ENSMUSG00000028413|ENSMUSG00000034807
Genetic Information Processing	Folding, sorting and degradation	Protein export	mmu03060	3	31	0.107230761	ENSMUSG00000054408|ENSMUSG00000019802|ENSMUSG00000079108
Human Diseases	Endocrine and metabolic diseases	Type II diabetes mellitus	mmu04930	4	50	0.110230929	ENSMUSG00000021936|ENSMUSG00000004113|ENSMUSG00000015968|ENSMUSG00000020027
Metabolism	Glycan biosynthesis and metabolism	Glycosphingolipid biosynthesis - ganglio series	mmu00604	2	15	0.111708594	ENSMUSG00000031749|ENSMUSG00000027822
Metabolism	Glycan biosynthesis and metabolism	Glycosphingolipid biosynthesis - globo series	mmu00603	2	15	0.111708594	ENSMUSG00000031749|ENSMUSG00000055978
Cellular Processes	Cell growth and death	Apoptosis - multiple species	mmu04215	3	32	0.114479879	ENSMUSG00000021936|ENSMUSG00000020486|ENSMUSG00000025860
Organismal Systems	Endocrine system	Thyroid hormone synthesis	mmu04918	5	71	0.115647956	ENSMUSG00000053007|ENSMUSG00000052889|ENSMUSG00000028597|ENSMUSG00000020048|ENSMUSG00000027523
Metabolism	Amino acid metabolism	Arginine and proline metabolism	mmu00330	4	51	0.115872849	ENSMUSG00000019987|ENSMUSG00000080845|ENSMUSG00000006442|ENSMUSG00000029455
Cellular	Cell growth	Oocyte	mmu0	7	116	0.12523	ENSMUSG00000031309|ENSMUSG00000020300|ENSMUSG0000001

Processes	and death	meiosis	4114			7593	4956|ENSMUSG00000024974|ENSMUSG00000020687|ENSMUSG00000025060|ENSMUSG00000046532
Cellular Processes	Cellular community - eukaryotes	Signaling pathways regulating pluripotency of stem cells	mmu04550	8	140	0.131221091	ENSMUSG00000020135|ENSMUSG00000067336|ENSMUSG00000036202|ENSMUSG00000015957|ENSMUSG00000021796|ENSMUSG00000020644|ENSMUSG00000037035|ENSMUSG00000036904
Metabolism	Carbohydrate metabolism	Starch and sucrose metabolism	mmu00500	4	54	0.133526025	ENSMUSG00000003865|ENSMUSG00000025579|ENSMUSG00000001891|ENSMUSG00000029201
Genetic Information Processing	Replication and repair	DNA replication	mmu03030	3	35	0.1372678	ENSMUSG00000024925|ENSMUSG00000020471|ENSMUSG00000020630
Metabolism	Carbohydrate metabolism	Fructose and mannose metabolism	mmu00051	3	35	0.1372678	ENSMUSG00000022474|ENSMUSG00000026773|ENSMUSG00000101904
Organismal Systems	Immune system	Antigen processing and presentation	mmu04612	6	98	0.142148434	ENSMUSG00000067235|ENSMUSG00000059970|ENSMUSG00000021270|ENSMUSG00000055413|ENSMUSG00000027248|ENSMUSG00000038642
Genetic Information Processing	Folding, sorting and degradation	Protein processing in endoplasmic reticulum	mmu04141	9	167	0.145309469	ENSMUSG00000059970|ENSMUSG00000024079|ENSMUSG00000027828|ENSMUSG00000021270|ENSMUSG00000019802|ENSMUSG00000024807|ENSMUSG00000020048|ENSMUSG00000027248|ENSMUSG00000021936
Organismal Systems	Endocrine system	Melanogenesis	mmu04916	6	99	0.146741233	ENSMUSG00000015957|ENSMUSG00000021367|ENSMUSG00000019966|ENSMUSG00000027523|ENSMUSG00000036904|ENSMUSG00000052889
Organismal	Endocrine	Regulation	mmu0	4	57	0.15219	ENSMUSG00000027523|ENSMUSG00000052920|ENSMUSG0000008

Systems	system	of lipolysis in adipocytes	4923			6792	3816|ENSMUSG00000025509
Human Diseases	Immune diseases	Primary immunodeficiency	mmu05340	3	37	0.153242672	ENSMUSG00000026648|ENSMUSG00000094694|ENSMUSG00000026395
Metabolism	Lipid metabolism	Steroid biosynthesis	mmu00100	2	19	0.158150581	ENSMUSG00000026675|ENSMUSG00000021273
Metabolism	Amino acid metabolism	Alanine, aspartate and glutamate metabolism	mmu00250	3	38	0.161439687	ENSMUSG00000025991|ENSMUSG00000022546|ENSMUSG00000057880
Cellular Processes	Cell growth and death	Cell cycle	mmu04110	7	125	0.162095399	ENSMUSG00000002297|ENSMUSG00000025862|ENSMUSG00000037286|ENSMUSG00000024974|ENSMUSG00000020687|ENSMUSG00000069089|ENSMUSG00000015312
Environmental Information Processing	Signal transduction	cGMP-PKG signaling pathway	mmu04022	9	173	0.166925503	ENSMUSG00000053093|ENSMUSG00000053007|ENSMUSG00000015968|ENSMUSG00000019907|ENSMUSG00000014956|ENSMUSG00000052920|ENSMUSG00000027335|ENSMUSG00000054640|ENSMUSG00000044951
Environmental Information Processing	Signaling molecules and interaction	ECM-receptor interaction	mmu04512	5	83	0.178812591	ENSMUSG00000027009|ENSMUSG00000038486|ENSMUSG00000020592|ENSMUSG00000079465|ENSMUSG00000103735
Human Diseases	Cancers: Specific types	Small cell lung cancer	mmu05222	5	85	0.190409527	ENSMUSG00000083816|ENSMUSG00000017386|ENSMUSG00000079465|ENSMUSG00000027164|ENSMUSG00000025860
Organismal Systems	Excretory system	Proximal tubule bicarbonate reclamation	mmu04964	2	22	0.194900244	ENSMUSG00000025792|ENSMUSG00000004655
Environmental Information	Signal transduction	ErbB signaling	mmu04012	5	87	0.202264956	ENSMUSG00000021936|ENSMUSG00000062209|ENSMUSG00000052889|ENSMUSG00000027646|ENSMUSG00000004043

Processing		pathway					
Organismal Systems	Excretory system	Vasopressin-regulated water reabsorption	mmu04962	3	43	0.204188176	ENSMUSG00000009013|ENSMUSG00000027523|ENSMUSG00000053007
Organismal Systems	Nervous system	Long-term potentiation	mmu04720	4	66	0.213299446	ENSMUSG00000031309|ENSMUSG00000068798|ENSMUSG00000052889|ENSMUSG00000014956
Human Diseases	Cancers: Specific types	Pancreatic cancer	mmu05212	4	66	0.213299446	ENSMUSG00000033220|ENSMUSG00000021936|ENSMUSG00000026104|ENSMUSG00000007613
Environmental Information Processing	Signaling molecules and interaction	Neuroactive ligand-receptor interaction	mmu04080	13	285	0.214439586	ENSMUSG00000028036|ENSMUSG00000027335|ENSMUSG00000059588|ENSMUSG00000059481|ENSMUSG00000045613|ENSMUSG00000032860|ENSMUSG00000049130|ENSMUSG00000027249|ENSMUSG00000068037|ENSMUSG00000038668|ENSMUSG00000040552|ENSMUSG00000053004|ENSMUSG00000057722
Metabolism	Glycan biosynthesis and metabolism	Glycosaminoglycan biosynthesis - heparan sulfate / heparin	mmu00534	2	24	0.219943084	ENSMUSG00000044499|ENSMUSG00000028838
Organismal Systems	Endocrine system	Progesterone-mediated oocyte maturation	mmu04914	5	90	0.220491676	ENSMUSG00000031309|ENSMUSG00000020687|ENSMUSG00000020300|ENSMUSG00000021270|ENSMUSG00000021936
Organismal Systems	Digestive system	Protein digestion and absorption	mmu04974	5	90	0.220491676	ENSMUSG00000054640|ENSMUSG00000001865|ENSMUSG00000021999|ENSMUSG00000079465|ENSMUSG00000035000
Cellular Processes	Cell growth and death	p53 signaling	mmu04115	4	68	0.22771404	ENSMUSG00000022292|ENSMUSG00000037411|ENSMUSG00000020649|ENSMUSG00000015312

		pathway					
Human Diseases	Cancers: Overview	Chemical carcinogenesis	mmu05204	5	93	0.239193397	ENSMUSG00000024885|ENSMUSG00000032315|ENSMUSG00000024087|ENSMUSG00000083816|ENSMUSG00000003053
Organismal Systems	Endocrine system	Thyroid hormone signaling pathway	mmu04919	6	117	0.239419571	ENSMUSG00000013833|ENSMUSG00000101904|ENSMUSG00000027646|ENSMUSG00000026104|ENSMUSG00000052889|ENSMUSG00000015468
Metabolism	Carbohydrate metabolism	Inositol phosphate metabolism	mmu00562	4	70	0.242364379	ENSMUSG00000019139|ENSMUSG00000027296|ENSMUSG00000025178|ENSMUSG00000030660
Organismal Systems	Endocrine system	Insulin signaling pathway	mmu04910	7	142	0.242684596	ENSMUSG00000003865|ENSMUSG00000020027|ENSMUSG00000006542|ENSMUSG00000014956|ENSMUSG00000028518|ENSMUSG00000021936|ENSMUSG00000029167
Environmental Information Processing	Signal transduction	Hippo signaling pathway -multiple species	mmu04392	2	26	0.245226792	ENSMUSG00000006262|ENSMUSG00000022329
Organismal Systems	Development	Dorso-ventral axis formation	mmu04320	2	26	0.245226792	ENSMUSG00000020300|ENSMUSG00000015468
Organismal Systems	Digestive system	Bile secretion	mmu04976	4	71	0.249768207	ENSMUSG00000027523|ENSMUSG00000021135|ENSMUSG00000029802|ENSMUSG00000004655
Human Diseases	Drug resistance: Antineoplastic	Endocrine resistance	mmu01522	5	95	0.251892993	ENSMUSG00000021936|ENSMUSG00000015468|ENSMUSG00000027314|ENSMUSG00000027646|ENSMUSG00000027523
Human Diseases	Immune diseases	Allograft rejection	mmu05330	4	72	0.257219332	ENSMUSG00000067235|ENSMUSG00000055413|ENSMUSG00000094694|ENSMUSG00000015437
Organismal Systems	Endocrine system	Renin secretion	mmu04924	4	72	0.257219332	ENSMUSG00000027523|ENSMUSG00000004655|ENSMUSG00000004347|ENSMUSG00000015968

Organismal Systems	Aging	Longevity regulating pathway	mmu04211	5	96	0.258305088	ENSMUSG00000053007|ENSMUSG00000029167|ENSMUSG00000006542|ENSMUSG00000028518|ENSMUSG00000040760
Metabolism	Glycan biosynthesis and metabolism	N-Glycan biosynthesis	mmu00510	3	49	0.258315296	ENSMUSG00000028541|ENSMUSG00000028413|ENSMUSG00000032123
Human Diseases	Substance dependence	Cocaine addiction	mmu05030	3	49	0.258315296	ENSMUSG00000027523|ENSMUSG00000020182|ENSMUSG00000053007
Human Diseases	Cancers: Overview	Choline metabolism in cancer	mmu05231	5	101	0.290892547	ENSMUSG00000033220|ENSMUSG00000021936|ENSMUSG00000052889|ENSMUSG00000029684|ENSMUSG00000057193
Metabolism	Amino acid metabolism	Phenylalanine, tyrosine and tryptophan biosynthesis	mmu00400	1	9	0.291750046	ENSMUSG00000001670
Genetic Information Processing	Transcription	RNA polymerase	mmu03020	2	30	0.29596175	ENSMUSG00000027427|ENSMUSG00000035834
Human Diseases	Infectious diseases: Bacterial	Bacterial invasion of epithelial cells	mmu05100	4	78	0.302708732	ENSMUSG00000022831|ENSMUSG00000027646|ENSMUSG00000061665|ENSMUSG00000029684
Organismal Systems	Digestive system	Salivary secretion	mmu04970	4	78	0.302708732	ENSMUSG00000027523|ENSMUSG00000052920|ENSMUSG00000052889|ENSMUSG00000027335
Human Diseases	Infectious diseases: Bacterial	Salmonella infection	mmu05132	4	78	0.302708732	ENSMUSG00000021936|ENSMUSG00000025888|ENSMUSG00000029684|ENSMUSG00000039005
Organismal Systems	Excretory system	Endocrine and other	mmu04961	3	54	0.304699958	ENSMUSG00000027523|ENSMUSG00000052889|ENSMUSG00000054640

		factor-regulated calcium reabsorption					
Metabolism	Carbohydrate metabolism	Pentose phosphate pathway	mmu00030	2	31	0.308593542	ENSMUSG00000023070|ENSMUSG00000026005
Human Diseases	Cancers: Specific types	Basal cell carcinoma	mmu05217	3	55	0.314036718	ENSMUSG00000015957|ENSMUSG00000036904|ENSMUSG00000020135
Environmental Information Processing	Signal transduction	mTOR signaling pathway	mmu04150	7	156	0.316623941	ENSMUSG00000050310|ENSMUSG00000031309|ENSMUSG00000015957|ENSMUSG00000035992|ENSMUSG00000028518|ENSMUSG00000036904|ENSMUSG00000052889
Organismal Systems	Immune system	T cell receptor signaling pathway	mmu04660	5	105	0.31745305	ENSMUSG00000004266|ENSMUSG00000024235|ENSMUSG00000034116|ENSMUSG00000030595|ENSMUSG00000026395
Metabolism	Carbohydrate metabolism	Citrate cycle (TCA cycle)	mmu00020	2	32	0.321178716	ENSMUSG00000024892|ENSMUSG00000021913
Genetic Information Processing	Folding, sorting and degradation	SNARE interactions in vesicular transport	mmu04130	2	33	0.333705918	ENSMUSG00000051412|ENSMUSG00000039232
Metabolism	Lipid metabolism	Synthesis and degradation of ketone bodies	mmu00072	1	11	0.338972853	ENSMUSG00000046598
Genetic Information Processing	Translation	Ribosome biogenesis in eukaryotes	mmu03008	4	83	0.341245266	ENSMUSG00000041747|ENSMUSG00000030521|ENSMUSG00000042354|ENSMUSG00000021428

Genetic Information Processing	Replication and repair	Base excision repair	mmu03410	2	35	0.358545065	ENSMUSG00000066551|ENSMUSG00000020471
Organismal Systems	Nervous system	Long-term depression	mmu04730	3	61	0.369988687	ENSMUSG00000027523|ENSMUSG00000052920|ENSMUSG00000052889
Human Diseases	Immune diseases	Autoimmune thyroid disease	mmu05320	4	87	0.372182811	ENSMUSG00000067235|ENSMUSG00000055413|ENSMUSG00000094694|ENSMUSG00000015437
Organismal Systems	Nervous system	Synaptic vesicle cycle	mmu04721	3	62	0.379254207	ENSMUSG00000034799|ENSMUSG00000004113|ENSMUSG00000031840
Human Diseases	Cancers: Specific types	Prostate cancer	mmu05215	4	89	0.387619725	ENSMUSG00000021270|ENSMUSG00000020048|ENSMUSG00000046532|ENSMUSG00000053007
Organismal Systems	Aging	Longevity regulating pathway - multiple species	mmu04213	3	64	0.397691584	ENSMUSG00000059970|ENSMUSG00000006542|ENSMUSG00000028518
Metabolism	Metabolism of cofactors and vitamins	Folate biosynthesis	mmu00790	1	14	0.40397353	ENSMUSG00000073987
Environmental Information Processing	Signal transduction	Phospholipase D signaling pathway	mmu04072	6	146	0.411422149	ENSMUSG00000038387|ENSMUSG00000028036|ENSMUSG00000019966|ENSMUSG00000094694|ENSMUSG00000038668|ENSMUSG00000027523
Cellular Processes	Transport and catabolism	Regulation of autophagy	mmu04140	2	40	0.418981814	ENSMUSG00000079418|ENSMUSG00000028518
Genetic Information Processing	Translation	Ribosome	mmu03010	6	148	0.423507834	ENSMUSG00000023939|ENSMUSG00000029486|ENSMUSG00000024181|ENSMUSG00000031781|ENSMUSG00000015672|ENSMUSG00000003299
Metabolism	Amino acid	Glycine,	mmu0	2	41	0.43072	ENSMUSG00000017713|ENSMUSG00000011752

	metabolism	serine and threonine metabolism	0260			673	
Organismal Systems	Nervous system	Neurotrophin signaling pathway	mmu04722	5	122	0.431774049	ENSMUSG00000031309|ENSMUSG00000021936|ENSMUSG00000068798|ENSMUSG00000027164|ENSMUSG00000030595
Environmental Information Processing	Signal transduction	Phosphatidylinositol signaling system	mmu04070	4	97	0.448642392	ENSMUSG00000030660|ENSMUSG00000027296|ENSMUSG00000052889|ENSMUSG00000025178
Metabolism	Energy metabolism	Nitrogen metabolism	mmu00910	1	17	0.462586749	ENSMUSG00000025991
Metabolism	Metabolism of other amino acids	Selenocompound metabolism	mmu00450	1	17	0.462586749	ENSMUSG00000003477
Genetic Information Processing	Transcription	Basal transcription factors	mmu03022	2	44	0.465175375	ENSMUSG00000069089|ENSMUSG00000001524
Organismal Systems	Immune system	Intestinal immune network for IgA production	mmu04672	2	44	0.465175375	ENSMUSG00000027009|ENSMUSG00000094694
Human Diseases	Immune diseases	Graft-versus-host disease	mmu05332	3	72	0.469614918	ENSMUSG00000067235|ENSMUSG00000055413|ENSMUSG00000015437
Organismal Systems	Digestive system	Carbohydrate digestion and absorption	mmu04973	2	45	0.476381669	ENSMUSG00000052889|ENSMUSG00000015968
Organismal	Digestive	Gastric acid	mmu0	3	73	0.47834	ENSMUSG00000027523|ENSMUSG00000052889|ENSMUSG0000004

Systems	system	secretion	4971			3503	4951
Metabolism	Glycan biosynthesis and metabolism	Other glycan degradation	mmu0 0511	1	18	0.48081 5304	ENSMUSG00000019810
Metabolism	Metabolism of cofactors and vitamins	Pantothenat e and CoA biosynthesis	mmu0 0770	1	18	0.48081 5304	ENSMUSG00000030826
Metabolism	Metabolism of cofactors and vitamins	One carbon pool by folate	mmu0 0670	1	19	0.49842 6003	ENSMUSG00000029376
Metabolism	Lipid metabolism	Sphingolipid metabolism	mmu0 0600	2	48	0.50912 178	ENSMUSG00000030760|ENSMUSG00000057342
Human Diseases	Endocrine and metabolic diseases	Type I diabetes mellitus	mmu0 4940	3	77	0.51255 436	ENSMUSG00000067235|ENSMUSG00000055413|ENSMUSG0000001 5437
Metabolism	Glycan biosynthesis and metabolism	Glycosamin oglycan biosynthesis - chondroitin sulfate / dermatan sulfate	mmu0 0532	1	20	0.51543 9774	ENSMUSG00000032997
Environmental Information Processing	Signal transduction	Notch signaling pathway	mmu0 4330	2	49	0.51973 3155	ENSMUSG00000027314|ENSMUSG00000015468
Metabolism	Glycan biosynthesis and metabolism	Glycosamin oglycan degradation	mmu0 0531	1	21	0.53187 6836	ENSMUSG00000035273

Genetic Information Processing	Replication and repair	Fanconi anemia pathway	mmu03460	2	51	0.540490207	ENSMUSG00000045102|ENSMUSG00000028560
Metabolism	Xenobiotics biodegradation and metabolism	Drug metabolism - other enzymes	mmu00983	2	51	0.540490207	ENSMUSG00000056973|ENSMUSG00000003500
Genetic Information Processing	Replication and repair	Mismatch repair	mmu03430	1	22	0.547756723	ENSMUSG00000020471
Human Diseases	Neurodegenerative diseases	Amyotrophic lateral sclerosis (ALS)	mmu05014	2	52	0.550632744	ENSMUSG00000018932|ENSMUSG00000025888
Metabolism	Glycan biosynthesis and metabolism	Glycosylphosphatidylinositol(GPI)-anchor biosynthesis	mmu00563	1	25	0.592238899	ENSMUSG00000010607
Human Diseases	Immune diseases	Asthma	mmu05310	1	26	0.60607254	ENSMUSG00000094694
Metabolism	Metabolism of cofactors and vitamins	Retinol metabolism	mmu00830	3	89	0.607393944	ENSMUSG00000024747|ENSMUSG00000032315|ENSMUSG00000003053
Organismal Systems	Sensory system	Taste transduction	mmu04742	3	89	0.607393944	ENSMUSG00000015085|ENSMUSG00000004347|ENSMUSG00000023439
Human Diseases	Endocrine and metabolic diseases	Maturity onset diabetes of the young	mmu04950	1	27	0.619437197	ENSMUSG00000017950
Genetic	Replication and	Homologous	mmu0	1	28	0.63234	ENSMUSG00000020471

Information Processing	repair	recombination	3440			8759	
Environmental Information Processing	Signal transduction	Sphingolipid signaling pathway	mmu04071	4	125	0.640484109	ENSMUSG00000033220|ENSMUSG00000021936|ENSMUSG00000052889|ENSMUSG00000057342
Genetic Information Processing	Translation	mRNA surveillance pathway	mmu03015	3	96	0.656710125	ENSMUSG00000028274|ENSMUSG00000014956|ENSMUSG00000027176
Metabolism	Carbohydrate metabolism	Glyoxylate and dicarboxylate metabolism	mmu00630	1	30	0.656873475	ENSMUSG00000032527
Human Diseases	Cancers: Overview	Central carbon metabolism in cancer	mmu05230	2	66	0.675823547	ENSMUSG00000011752|ENSMUSG00000034748
Metabolism	Metabolism of cofactors and vitamins	Nicotinate and nicotinamide metabolism	mmu00760	1	35	0.711269106	ENSMUSG00000029780
Human Diseases	Cancers: Specific types	Melanoma	mmu05218	2	72	0.720100634	ENSMUSG00000022523|ENSMUSG00000057967
Human Diseases	Cancers: Specific types	Chronic myeloid leukemia	mmu05220	2	73	0.726963256	ENSMUSG00000007613|ENSMUSG00000004043
Human Diseases	Substance dependence	Nicotine addiction	mmu05033	1	40	0.757046767	ENSMUSG00000004113
Organismal Systems	Excretory system	Aldosterone-regulated	mmu04960	1	40	0.757046767	ENSMUSG00000052889

		sodium reabsorption					
Human Diseases	Drug resistance: Antineoplastic	Platinum drug resistance	mmu01524	2	78	0.759165651	ENSMUSG00000025860|ENSMUSG00000017485
Human Diseases	Cancers: Specific types	Bladder cancer	mmu05219	1	41	0.765292253	ENSMUSG00000027646
Human Diseases	Drug resistance: Antineoplastic	EGFR tyrosine kinase inhibitor resistance	mmu01521	2	82	0.782509194	ENSMUSG00000052889|ENSMUSG00000027646
Environmental Information Processing	Signal transduction	Hedgehog signaling pathway	mmu04340	1	44	0.788388456	ENSMUSG00000029122
Genetic Information Processing	Folding, sorting and degradation	Proteasome	mmu03050	1	45	0.795570968	ENSMUSG00000036743
Human Diseases	Cancers: Specific types	Endometrial cancer	mmu05213	1	52	0.839470673	ENSMUSG00000020135
Human Diseases	Cancers: Overview	MicroRNAs in cancer	mmu05206	7	281	0.859674851	ENSMUSG00000083816|ENSMUSG00000024087|ENSMUSG00000020135|ENSMUSG00000067336|ENSMUSG00000024975|ENSMUSG00000052889|ENSMUSG00000015468
Human Diseases	Cancers: Specific types	Non-small cell lung cancer	mmu05223	1	56	0.860185332	ENSMUSG00000052889
Human Diseases	Cancers: Specific types	Glioma	mmu05214	1	65	0.897546528	ENSMUSG00000052889
Genetic Information	Translation	Aminoacyl-tRNA	mmu00970	1	66	0.901025828	ENSMUSG00000038838

Processing		biosynthesis					
Metabolism	Xenobiotics biodegradation and metabolism	Drug metabolism - cytochrome P450	mmu00982	1	67	0.904387056	ENSMUSG00000024885
Human Diseases	Cancers: Specific types	Renal cell carcinoma	mmu05211	1	68	0.907634216	ENSMUSG00000068798
Organismal Systems	Sensory system	Olfactory transduction	mmu04740	3	1102	1	ENSMUSG00000054640|ENSMUSG00000052920|ENSMUSG00000004347

Table SVI. The DEGs in the metabolic related pathways of DbCM / CK.

DbCM_vs_CK.DEG.kegg.Amino_acid_metabolism

Gene_ID	CK-1	CK-2	CK-3	DbCM-1	DbCM-2	DbCM-3	KEGG_ID	symbol	ncbi_descritption
ENSMUSG00000000326	3.21245641	1.799713905	2.383210404	3.920393156	3.772336607	4.310600146	K00545	Comt	catechol-O-methyltransferase
ENSMUSG00000001270	31.85306475	24.94336758	28.42768105	19.27965865	19.83888939	16.7197106	K00933	Ckb	creatine kinase, brain
ENSMUSG00000003477	25.02817991	26.46990988	30.98021296	83.92356779	82.47000452	74.70460531	K00562	Inmt	indolethylamine N-methyltransferase
ENSMUSG00000011179	46.83282395	48.12747328	47.15080372	63.71805663	58.85761995	54.6063596	K01581	Odc1	ornithine decarboxylase, structural 1
ENSMUSG00000019055	42.39805277	39.20767303	34.95852168	24.55025531	27.35876508	23.9888009	K00473	Plod1	procollagen-lysine, 2-oxoglutarate 5-dioxygenase 1
ENSMUSG00000019916	9.835309773	10.76966918	11.56867269	5.760274564	6.564762083	7.777530333	K00472	P4ha1	procollagen-proline, 2-oxoglutarate 4-dioxygenase (proline 4-hydroxylase), alpha 1 polypeptide
ENSMUSG00000020534	5.394468117	5.83667113	6.410440815	7.117940109	7.557594555	7.147986931	K00600	Shmt1	serine hydroxymethyltransferase 1 (soluble)
ENSMUSG00000020826	8.360840499	7.025175733	7.464902429	4.942921967	5.113542907	4.962047295	K13241	Nos2	nitric oxide synthase 2, inducible
ENSMUSG00000022186	262.4739732	263.2462315	289.4328376	153.3752575	177.4125371	173.2131516	K01027	Oxct1	3-oxoacid CoA transferase 1
ENSMUSG00000024087	3.280423378	2.885787564	2.523088647	6.34827456	5.341697863	5.780421416	K07410	Cyp1b1	cytochrome P450, family 1, subfamily b, polypeptide 1
ENSMUSG00000024640	2.849778267	2.012304495	1.826845735	3.761648644	3.651581576	4.143651764	K00831	Psat1	phosphoserine aminotransferase 1
ENSMUSG00000024827	0.178174544	0.219436138	0.124681876	0.516418331	0.383583826	0.503034104	K00281	Gldc	glycine decarboxylase

ENSMUSG00000024903	0	0.012524528	0	0.235800574	0.445829539	0.121804896	K03334	Lao1	L-amino acid oxidase 1
ENSMUSG00000025190	383.8186428	364.0203343	401.4343311	507.3705724	527.5387285	494.383701	K14454	Got1	glutamic-oxaloacetic transaminase 1, soluble
ENSMUSG00000025745	507.0203499	498.376921	532.2159723	630.924722	650.9487928	648.9368613	K07515	Hadha	hydroxyacyl-Coenzyme A dehydrogenase/3-ketoacyl-Coenzyme A thiolase/enoyl-Coenzyme A hydratase (trifunctional protein), alpha subunit
ENSMUSG00000026473	241.0150024	212.4001753	232.5191055	272.3307751	337.8396187	283.7626977	K01915	Glul	glutamate-ammonia ligase (glutamine synthetase)
ENSMUSG00000026687	18.29652714	19.96624489	17.04057891	23.65845339	22.5334987	24.20158882	K00149	Aldh9a1	aldehyde dehydrogenase 9, subfamily A1
ENSMUSG00000027187	132.0968003	130.4258057	133.5510921	159.4836847	164.7355343	168.1569173	K03781	Cat	catalase
ENSMUSG00000027875	28.89953743	34.81231421	32.25377747	93.69084259	97.12560815	90.61036974	K01641	Hmgcs2	3-hydroxy-3-methylglutaryl-Coenzyme A synthase 2
ENSMUSG00000032315	0.768812919	1.668994376	1.301601119	8.523049765	10.83608469	9.293320655	K07408	Cyp1a1	cytochrome P450, family 1, subfamily a, polypeptide 1
ENSMUSG00000032350	4.777623142	5.440529881	4.824914376	6.156356375	6.157165774	6.168750784	K11204	Gclc	glutamate-cysteine ligase, catalytic subunit
ENSMUSG00000032374	5.432377938	6.626152378	6.690161732	4.543282417	3.761378653	4.561668178	K13645	Plod2	procollagen lysine, 2-oxoglutarate 5-dioxygenase 2
ENSMUSG00000032649	4.461293831	4.708081198	4.435347441	3.174045515	3.390205236	3.082794726	K11703	Colgalt2	collagen beta(1-O)galactosyltransferase 2
ENSMUSG00000033307	172.1218364	148.8657898	143.927147	208.927722	222.3777539	169.4305496	K07253	Mif	macrophage migration inhibitory factor (glycosylation-inhibiting factor)
ENSMUSG00000036880	795.3339203	766.3794719	808.361652	1063.602428	1116.746467	1042.098943	K07508	Acaa2	acetyl-Coenzyme A acyltransferase 2 (mitochondrial 3-oxoacyl-Coenzyme A thiolase)
ENSMUSG00000039783	0.73772122	0.753129345	0.518969391	0.32225553	0.089124931	0.257133718	K00486	Kmo	kynurenine 3-monooxygenase (kynurenine 3-hydroxylase)
ENSMUSG00000048087	3.4194	3.76712	3.9294	4.330504	5.42577	4.319819	K01251	Gm4737	predicted gene 4737

	35961	4337	83229	214	9306	661			
ENSMUSG00000057880	5.50415988	5.406803129	6.245166559	3.024941367	3.346083641	3.042644793	K13524	Abat	4-aminobutyrate aminotransferase
ENSMUSG00000059447	330.7066819	336.4473266	355.9561331	407.2224733	414.2221469	427.3146874	K07509	Hadhb	hydroxyacyl-Coenzyme A dehydrogenase/3-ketoacyl-Coenzyme A thiolase/enoyl-Coenzyme A hydratase (trifunctional protein), beta subunit
ENSMUSG00000063558	7.126676922	8.731308507	8.871665566	9.528046683	10.14022955	11.04602734	K00157	Aox1	aldehyde oxidase 1
ENSMUSG00000069835	3.785419911	3.127335225	3.35641787	5.303044342	4.992750555	4.167885408	K00657	Sat2	spermidine/spermine N1-acetyl transferase 2
ENSMUSG00000074207	6.321064398	5.264860001	5.400298678	9.837957108	8.664233558	7.920069471	K13951	Adh1	alcohol dehydrogenase 1 (class I)

DbCM_vs_CK.DEG.kegg.Lipid_metabolism

Gene_ID	CK-1	CK-2	CK-3	DbCM-1	DbCM-2	DbCM-3	KEGG_ID	symbol	ncbi_descritption
ENSMUSG00000000320	4.301612039	3.80373383	3.486967527	6.543857002	5.539870276	5.682350609	K00458	Alox12	arachidonate 12-lipoxygenase
ENSMUSG00000000326	3.21245641	1.799713905	2.383210404	3.920393156	3.772336607	4.310600146	K00545	Comt	catechol-O-methyltransferase
ENSMUSG00000006344	3.97376909	3.295586753	3.434228384	5.320868379	6.011021866	5.813225671	K18592	Ggt5	gamma-glutamyltransferase 5
ENSMUSG00000015090	201.31543	158.0970996	240.2256269	321.9894956	503.8477484	299.1643445	K01830	Ptgds	prostaglandin D2 synthase (brain)
ENSMUSG00000018574	307.3765826	308.0427333	314.8489228	407.6293108	398.8035131	403.4974049	K09479	Acadvl	acyl-Coenzyme A dehydrogenase, very long chain
ENSMUSG00000018924	0.138826741	0.476290804	0.069390939	0.020902508	0.01358517	0.02375423	K00460	Alox15	arachidonate 15-lipoxygenase
ENSMUSG00000020777	119.991	115.297	120.934	146.945	158.962	156.027	K00232	Acox1	acyl-Coenzyme A oxidase 1, palmitoyl

	7967	0457	111	1785	8243	4527			
ENSMUSG00000021226	71.45548929	69.03545566	71.59986506	99.60764439	104.8266982	99.13865231	K01068	Acot2	acyl-CoA thioesterase 2
ENSMUSG00000021608	6.661371695	6.604515962	5.740392345	5.172432137	4.007347911	4.180851141	K13510	Lpcat1	lysophosphatidylcholine acyltransferase 1
ENSMUSG00000022040	182.0239201	190.0091361	186.5322965	259.9114956	275.7564076	265.4797147	K08726	Ephx2	epoxide hydrolase 2, cytoplasmic
ENSMUSG00000022186	262.4739732	263.2462315	289.4328376	153.3752575	177.4125371	173.2131516	K01027	Oxct1	3-oxoacid CoA transferase 1
ENSMUSG00000022425	7.099285567	8.745236586	7.410252784	10.07896289	9.812715357	9.189110914	K01122	Enpp2	ectonucleotide pyrophosphatase/phosphodiesterase 2
ENSMUSG00000023019	1.472863898	2.252011718	1.939812668	2.684028819	3.088501273	2.970204516	K00006	Gpd1	glycerol-3-phosphate dehydrogenase 1 (soluble)
ENSMUSG00000024087	3.280423378	2.885787564	2.523088647	6.34827456	5.341697863	5.780421416	K07410	Cyp1b1	cytochrome P450, family 1, subfamily b, polypeptide 1
ENSMUSG00000024843	1.689421769	1.675909477	1.717622639	2.532860902	2.145665467	1.937396943	K14156	Chka	choline kinase alpha
ENSMUSG00000024900	32.89115765	32.69053882	35.63288305	46.92888808	47.46128903	47.22305855	K08765	Cpt1a	carnitine palmitoyltransferase 1a, liver
ENSMUSG00000025509	322.3175677	289.5387776	285.927942	463.8335701	474.1939309	426.5091009	K16816	Pnpla2	patatin-like phospholipase domain containing 2
ENSMUSG00000025745	507.0203499	498.376921	532.2159723	630.924722	650.9487928	648.9368613	K07515	Hadha	hydroxyacyl-Coenzyme A dehydrogenase/3-ketoacyl-Coenzyme A thiolase/enoyl-Coenzyme A hydratase (trifunctional protein), alpha subunit
ENSMUSG00000026003	531.2188303	544.5024808	557.5524076	714.9709796	757.3936353	773.8671787	K00255	Acadl	acyl-Coenzyme A dehydrogenase, long-chain
ENSMUSG00000026675	2.820786604	3.297961895	2.986602871	3.613881301	4.257589931	3.666451502	K13373	Hsd17b7	hydroxysteroid (17-beta) dehydrogenase 7
ENSMUSG00000026687	18.29652714	19.96624489	17.04057891	23.65845339	22.5334987	24.20158882	K00149	Aldh9a1	aldehyde dehydrogenase 9, subfamily A1

ENSMUSG00000027875	28.89953743	34.81231421	32.25377747	93.69084259	97.12560815	90.61036974	K01641	Hmgcs2	3-hydroxy-3-methylglutaryl-Coenzyme A synthase 2
ENSMUSG00000027999	17.45940222	17.55177408	16.29166591	21.2305309	22.49958879	20.46444385	K01047	Pla2g12a	phospholipase A2, group XIIA
ENSMUSG00000028497	2.79409285	3.363197085	3.629003776	2.539225897	1.944020414	2.262057465	K10703	Hacd4	3-hydroxyacyl-CoA dehydratase 4
ENSMUSG00000028937	67.06449601	61.77247065	63.15531718	79.02756164	81.1317096	74.54321246	K17360	Acot7	acyl-CoA thioesterase 7
ENSMUSG00000029330	1.585235813	1.222089449	1.593243549	2.41889794	2.70220249	2.151001829	K00981	Cds1	CDP-diacylglycerol synthase 1
ENSMUSG00000029925	1.422185933	1.599339962	1.187214779	0.96580169	0.774765642	0.890596176	K01832	Tbxas1	thromboxane A synthase 1, platelet
ENSMUSG00000030483	0.104728691	0.19961457	0.418779388	0.578179068	1.434781141	0.895990713	K07412	Cyp2b10	cytochrome P450, family 2, subfamily b, polypeptide 10
ENSMUSG00000031903	9.76358314	7.991051615	8.104861689	6.698998759	5.067434094	6.872794	K06129	Pla2g15	phospholipase A2, group XV
ENSMUSG00000032315	0.768812919	1.668994376	1.301601119	8.523049765	10.83608469	9.293320655	K07408	Cyp1a1	cytochrome P450, family 1, subfamily a, polypeptide 1
ENSMUSG00000036880	795.3339203	766.3794719	808.361652	1063.602428	1116.746467	1042.098943	K07508	Acaa2	acetyl-Coenzyme A acyltransferase 2 (mitochondrial 3-oxoacyl-Coenzyme A thiolase)
ENSMUSG00000038732	2.567252917	2.379940582	2.232673173	1.222019593	1.104032396	1.38873934	K13517	Mboat1	membrane bound O-acyltransferase domain containing 1
ENSMUSG00000041193	13.99682865	15.15348074	13.72940201	10.32820841	9.636425811	9.012333089	K01047	Pla2g5	phospholipase A2, group V
ENSMUSG00000041202	3.904554954	2.973237437	2.885042771	2.14940379	1.48002497	1.425977282	K01047	Pla2g2d	phospholipase A2, group IID
ENSMUSG00000042010	79.09119166	75.43173262	74.37107502	90.01346983	100.1053414	91.27635204	K01946	Acacb	acetyl-Coenzyme A carboxylase beta
ENSMUSG00000047250	7.675525704	7.152361786	7.486466679	10.63461185	10.09081979	9.051641744	K00509	Ptgs1	prostaglandin-endoperoxide synthase 1
ENSMUSG00000050737	0.87115	0.87929	0.86171	1.19430	1.12169	1.27094	K15729	Ptges	prostaglandin E synthase

	969	6053	1028	5777	8471	5752			
ENSMUSG00000052396	1.86005 2156	2.49575 1461	2.32900 9412	1.06083 1615	1.37893 2919	1.36630 1943	K14457	Mogat2	monoacylglycerol O-acyltransferase 2
ENSMUSG00000059447	330.706 6819	336.447 3266	355.956 1331	407.222 4733	414.222 1469	427.314 6874	K07509	Hadhb	hydroxyacyl-Coenzyme A dehydrogenase/3-ketoacyl-Coenzyme A thiolase/enoyl-Coenzyme A hydratase (trifunctional protein), beta subunit
ENSMUSG00000061740	16.8594 7001	14.7793 2283	14.6009 1549	25.0362 9213	23.2977 71	23.3577 4133	K07414	Cyp2d22	cytochrome P450, family 2, subfamily d, polypeptide 22
ENSMUSG00000063275	20.4653 4092	20.9673 2188	20.2448 7307	13.5950 8116	13.1185 8172	13.0248 5206	K10703	Hacd1	3-hydroxyacyl-CoA dehydratase 1
ENSMUSG00000072949	32.8546 1548	35.0159 0604	39.6635 74	75.2903 2821	80.1070 841	69.3892 2867	K01068	Acot1	acyl-CoA thioesterase 1
ENSMUSG00000074207	6.32106 4398	5.26486 0001	5.40029 8678	9.83795 7108	8.66423 3558	7.92006 9471	K13951	Adh1	alcohol dehydrogenase 1 (class I)
ENSMUSG00000083816	0.44295 8437	0.36183 6817	0.47444 4955	0.11909 6832	0.12384 7397	0.05413 807		Gm13033	prostaglandin-endoperoxide synthase 2 pseudogene
ENSMUSG00000107653	2.01889 0619	1.87254 0227	1.62336 5506	2.84150 6626	2.96343 9039	2.68471 8979		Gm31520	sterol-C5-desaturase pseudogene

Table SVII. The DEGs in the metabolic related pathways of SPM / DbCM.

SPM_vs_DbCM.DEG.kegg.Amino_acid_metabolism

Gene_ID	DbCM-1	DbCM-2	DbCM-3	SPM-1	SPM-2	SPM-3	KEGG_ID	symbol	ncbi_descritption
ENSMUSG00000001670	0.212017434	0.468508184	0.337320069	2.49275388	1.679611934	1.284326567	K00815	Tat	tyrosine aminotransferase
ENSMUSG00000003477	83.92356779	82.47000452	74.70460531	59.41223561	56.89975689	52.3661326	K00562	Inmt	indolethylamine N-methyltransferase
ENSMUSG00000003809	30.03356106	30.00826139	29.29855175	19.9244392	22.27838993	23.69464968	K00252	Gcdh	glutaryl-Coenzyme A dehydrogenase
ENSMUSG00000006442	11.29016607	10.00389381	10.11469496	7.396899276	6.361829449	7.07665458	K00797	Srm	spermidine synthase
ENSMUSG00000010651	0.268316913	0.096881808	0.152461652	1.239213726	0.762470511	0.395054936	K07513	Acaa1b	acetyl-Coenzyme A acyltransferase 1B
ENSMUSG00000011752	27.99591218	28.11526988	27.69001148	18.07744718	21.03153498	23.21857789	K01834	Pgam1	phosphoglycerate mutase 1
ENSMUSG00000017713	3.468174246	3.777092006	3.430027689	2.192997025	2.268634678	2.472339598	K01620	Tha1	threonine aldolase 1
ENSMUSG00000019987	0.068184348	0.132945191	0.077486717	3.036606997	1.36103196	0.107561625	K01476	Arg1	arginase, liver
ENSMUSG00000020182	1.28505364	1.216303566	1.715583936	1.096448865	0.638162881	0.51958981	K01593	Ddc	dopa decarboxylase
ENSMUSG00000021033	15.54590768	14.56795301	14.18814333	11.81240455	10.82534719	10.70988445	K01800	Gstz1	glutathione transferase zeta 1 (maleylacetoacetate isomerase)
ENSMUSG00000021913	75.04430554	90.47962978	78.41969994	54.42798721	61.25089362	64.76783255	K00164	Ogdhl	oxoglutarate dehydrogenase-like
ENSMUSG00000022546	14.84226008	15.86146604	13.61211416	8.488146822	9.185539896	10.98028505	K00814	Gpt	glutamic pyruvic transaminase, soluble
ENSMUSG00000022821	0	0	0	0.38649	0.258367	0.018668	K00451	Hgd	homogentisate 1, 2-dioxygenase

				2392	168	451			
ENSMUSG00000022853	15.63326306	14.95261013	12.88066551	8.10451834	9.124829551	10.4029303	K07514	Ehhadh	enoyl-Coenzyme A, hydratase/3-hydroxyacyl Coenzyme A dehydrogenase
ENSMUSG00000023262	8.000565056	8.437752709	7.892957268	4.980765764	6.015402502	6.405309053	K14677	Acy1	aminoacylase 1
ENSMUSG00000024087	6.34827456	5.341697863	5.780421416	9.078590363	9.016305909	8.724137169	K07410	Cyp1b1	cytochrome P450, family 1, subfamily b, polypeptide 1
ENSMUSG00000024885	3.276376086	2.618133968	3.128242064	3.811311572	4.255016899	4.202601932	K00129	Aldh3b1	aldehyde dehydrogenase 3 family, member B1
ENSMUSG00000025991	0.07704954	0.023565562	0.046356027	2.325292057	1.113912321	0.011439677	K01948	Cps1	carbamoyl-phosphate synthetase 1
ENSMUSG00000027332	126.4005714	133.9516632	118.3356733	71.53266195	85.71991189	95.49450229	K00253	Ivd	isovaleryl coenzyme A dehydrogenase
ENSMUSG00000028011	0.007338114	0	0.008339251	0.984771679	0.622525347	0.018521541	K00453	Tdo2	tryptophan 2,3-dioxygenase
ENSMUSG00000028124	2.121739153	2.769675628	2.667174167	1.166272965	1.11837593	1.20522907	K11205	Gclm	glutamate-cysteine ligase, modifier subunit
ENSMUSG00000029455	135.7947132	143.49974	127.7586987	101.0445231	102.287809	103.611691	K00128	Aldh2	aldehyde dehydrogenase 2, mitochondrial
ENSMUSG00000029482	1.834451559	2.243075567	2.349732648	1.504715799	1.570988158	1.500888154	K01907	Aacs	acetoacetyl-CoA synthetase
ENSMUSG00000029545	172.6801043	185.213889	160.1167145	112.9063718	127.9071286	136.738442	K00248	Acads	acyl-Coenzyme A dehydrogenase, short chain
ENSMUSG00000030630	15.72574109	18.31454913	14.4233212	8.734329049	8.897867191	9.040971961	K01555	Fah	fumarylacetoacetate hydrolase
ENSMUSG00000030826	58.66584049	64.82429251	57.40267908	38.44960243	42.57943072	45.6069458	K00826	Bcat2	branched chain aminotransferase 2, mitochondrial
ENSMUSG00000031173	0	0.010919811	0.00954687	0.32923426	0.272493062	0.021203673	K00611	Otc	ornithine transcarbamylase
ENSMUSG00000032263	28.73970	30.333661	29.9486	20.8513	23.07374	22.52340	K00167	Bckdhb	branched chain ketoacid dehydrogenase E1, beta polypeptide

	25	16	1596	6804	494	434			
ENSMUSG00000032315	8.523049765	10.83608469	9.293320655	35.56568992	22.02314951	11.06247986	K07408	Cyp1a1	cytochrome P450, family 1, subfamily a, polypeptide 1
ENSMUSG00000032374	4.543282417	3.761378653	4.561668178	5.803774188	6.422642426	6.566392034	K13645	Plod2	procollagen lysine, 2-oxoglutarate 5-dioxygenase 2
ENSMUSG00000032527	78.97135381	83.22731903	80.17518	55.03230644	60.36960471	64.94435558	K01966	Pccb	propionyl Coenzyme A carboxylase, beta polypeptide
ENSMUSG00000034807	27.23394356	27.81818365	28.89746528	20.00822591	21.00860334	22.1930456	K11703	Colgalt1	collagen beta(1-O)galactosyltransferase 1
ENSMUSG00000037798	0.036299121	0.009436757	0.033001119	2.638275608	1.123081934	0.018323935	K00789	Mat1a	methionine adenosyltransferase I, alpha
ENSMUSG00000044986	7.955686487	7.047483669	6.769942032	5.26920319	5.642598205	5.116616235	K01011	Tst	thiosulfate sulfurtransferase, mitochondrial
ENSMUSG00000057880	3.024941367	3.346083641	3.042644793	1.780096226	2.127428953	2.193180612	K13524	Abat	4-aminobutyrate aminotransferase
ENSMUSG00000059422	3.458964701	5.368155618	4.312045452	3.809133976	9.38902047	8.307196581		Gm8116	predicted gene 8116
ENSMUSG00000060376	74.62966241	78.46474432	64.54643491	46.02371538	52.59821428	55.0811132	K00166	Bckdha	branched chain ketoacid dehydrogenase E1, alpha polypeptide
ENSMUSG00000071711	38.73505915	31.53186161	29.10162161	23.84531588	23.40076771	22.49353498	K01011	Mpst	mercaptopyruvate sulfurtransferase
ENSMUSG00000080845	0.522965853	0.218501537	0.254705965	0.088725391	0.023301236	0		Gm9115	ornithine decarboxylase, structural 1 pseudogene

SPM_vs_DbCM.DEG.kegg.Lipid_metabolism

Gene_ID	DbCM-1	DbCM-2	DbCM-3	SPM-1	SPM-2	SPM-3	KEGG_ID	symbol	ncbi_descritption
ENSMUSG00000003053	0	0	0	1.504558781	0.868173325	0.011259286	K07413	Cyp2c29	cytochrome P450, family 2, subfamily c, polypeptide 29
ENSMUSG00000003809	30.03356	30.0082	29.29855	19.9244	22.2783	23.6946	K00252	Gcdh	glutaryl-Coenzyme A dehydrogenase

	106	6139	175	392	8993	4968			
ENSMUSG00000004270	35.32004515	34.5195392	33.92923888	25.54076111	25.83752559	26.69901644	K13515	Lpcat3	lysophosphatidylcholine acyltransferase 3
ENSMUSG00000010651	0.268316913	0.096881808	0.152461652	1.239213726	0.762470511	0.395054936	K07513	Acaa1b	acetyl-Coenzyme A acyltransferase 1B
ENSMUSG00000018924	0.020902508	0.01358517	0.02375423	0.384771342	0.169502223	0.145085407	K00460	Alox15	arachidonate 15-lipoxygenase
ENSMUSG00000020333	1.586844798	2.068312309	1.749138864	0.962869888	1.184571102	1.614128244	K01897	Acsl6	acyl-CoA synthetase long-chain family member 6
ENSMUSG00000021273	32.38024514	31.39919997	28.24058704	23.33496987	22.9332551	23.18157212	K00801	Fdft1	farnesyl diphosphate farnesyl transferase 1
ENSMUSG00000021364	0.039339172	0	0	0.311463093	0.163594092	0.008274402	K10205	Elovl2	elongation of very long chain fatty acids (FEN1/Elo2, SUR4/Elo3, yeast)-like 2
ENSMUSG00000022853	15.63326306	14.95261013	12.88066551	8.10451834	9.124829551	10.4029303	K07514	Ehhadh	enoyl-Coenzyme A, hydratase/3-hydroxyacyl Coenzyme A dehydrogenase
ENSMUSG00000022947	5.960725199	4.727186331	5.086572462	3.782280017	3.597392139	3.476097545	K00084	Cbr3	carbonyl reductase 3
ENSMUSG00000024087	6.34827456	5.341697863	5.780421416	9.078590363	9.016305909	8.724137169	K07410	Cyp1b1	cytochrome P450, family 1, subfamily b, polypeptide 1
ENSMUSG00000024978	67.58124882	69.40753771	66.51984384	39.67740692	51.893822	61.3086556	K00629	Gpam	glycerol-3-phosphate acyltransferase, mitochondrial
ENSMUSG00000025059	3.60832667	2.812684246	3.724830209	5.366925327	4.943104809	4.729449968	K00864	Gk	glycerol kinase
ENSMUSG00000025495	10.60160302	10.89986099	9.529428231	6.790307954	6.864451443	6.680894741	K08730	Ptdss2	phosphatidylserine synthase 2
ENSMUSG00000025509	463.8335701	474.1939309	426.5091009	315.9424659	324.0920493	335.4092728	K16816	Pnpla2	patatin-like phospholipase domain containing 2
ENSMUSG00000026675	3.613881301	4.257589931	3.666451502	5.543124598	5.13512349	4.774322844	K13373	Hsd17b7	hydroxysteroid (17-beta) dehydrogenase 7
ENSMUSG00000027195	10.49706455	8.259097692	8.935599494	14.10131958	13.88846753	13.11375275	K10251	Hsd17b12	hydroxysteroid (17-beta) dehydrogenase 12

ENSMUSG00000027999	21.2305309	22.49958879	20.46444385	15.12011741	14.44527085	15.35587438	K01047	Pla2g12a	phospholipase A2, group XIIA
ENSMUSG00000028597	10.60601179	7.845261794	8.323885793	6.680629624	6.433093362	6.828018651	K00432	Gpx7	glutathione peroxidase 7
ENSMUSG00000029233	4.672501342	4.039238217	4.691332034	5.171970922	6.182972391	6.097116196	K12345	Srd5a3	steroid 5 alpha-reductase 3
ENSMUSG00000029455	135.7947132	143.49974	127.7586987	101.0445231	102.287809	103.611691	K00128	Aldh2	aldehyde dehydrogenase 2, mitochondrial
ENSMUSG00000029545	172.6801043	185.213889	160.1167145	112.9063718	127.9071286	136.738442	K00248	Acads	acyl-Coenzyme A dehydrogenase, short chain
ENSMUSG00000030747	196.1518614	216.8901764	193.6430814	155.424214	153.5788358	155.0056697	K11160	Dgat2	diacylglycerol O-acyltransferase 2
ENSMUSG00000030760	0.588353055	0.432892521	0.51471269	0.872755841	0.700771307	0.633232551	K04711	Acer3	alkaline ceramidase 3
ENSMUSG00000031278	1.869206737	1.853271914	2.026681215	2.446401019	2.522323457	2.587631207	K01897	Acsl4	acyl-CoA synthetase long-chain family member 4
ENSMUSG00000032315	8.523049765	10.83608469	9.293320655	35.56568992	22.02314951	11.06247986	K07408	Cyp1a1	cytochrome P450, family 1, subfamily a, polypeptide 1
ENSMUSG00000036833	16.0192928	17.35591409	16.79775541	10.14387251	10.49950853	11.59161996	K14676	Pnpla7	patatin-like phospholipase domain containing 7
ENSMUSG00000038173	2.188429454	1.172102229	1.13411632	1.907131758	2.483733565	2.86410854	K08743	Enpp6	ectonucleotide pyrophosphatase/phosphodiesterase 6
ENSMUSG00000040774	4.026183956	4.233301192	5.673588953	6.842890632	6.920483557	6.571533688	K13644	Cept1	choline/ethanolaminephosphotransferase 1
ENSMUSG00000041193	10.32820841	9.636425811	9.012333089	5.029128835	6.833644699	7.551286606	K01047	Pla2g5	phospholipase A2, group V
ENSMUSG00000041202	2.14940379	1.48002497	1.425977282	0.469134995	0.768220342	0.835764252	K01047	Pla2g2d	phospholipase A2, group IID
ENSMUSG00000046598	7.229848155	8.559443357	4.479103478	1.002355607	2.324464065	3.979249472	K00019	Bdh1	3-hydroxybutyrate dehydrogenase, type 1
ENSMUSG00000050195	20.69911	25.8764	20.63246	33.0659	30.4979	28.5828	K00507	Scd4	stearoyl-coenzyme A desaturase 4

	59	4507	725	6201	7667	7842			
ENSMUSG00000050737	1.194305777	1.121698471	1.270945752	0.803467172	0.800969594	0.801530238	K15729	Ptges	prostaglandin E synthase
ENSMUSG00000052160	0.150000877	0.119987808	0.104901815	0.63034877	0.359877013	0.247549482	K16860	Pld4	phospholipase D family, member 4
ENSMUSG00000057342	8.408914416	9.675953117	8.241666697	5.789631873	6.258995616	6.671761435	K04718	Sphk2	sphingosine kinase 2
ENSMUSG00000068086	0	0	0.050137798	2.602317802	1.174208063	0.018559407	K07414	Cyp2d9	cytochrome P450, family 2, subfamily d, polypeptide 9
ENSMUSG00000071072	15.20695048	14.48155351	16.59494266	20.21270886	20.99762892	19.6838097	K15730	Ptges3	prostaglandin E synthase 3
ENSMUSG00000072949	75.29032821	80.1070841	69.38922867	57.06604613	54.29746144	50.25668547	K01068	Acot1	acyl-CoA thioesterase 1
ENSMUSG00000083816	0.119096832	0.123847397	0.05413807	0.396032542	0.56460856	0.571145107		Gm13033	prostaglandin-endoperoxide synthase 2 pseudogene
ENSMUSG00000094806	0.015277349	0.019858419	0.017361632	1.270043679	0.609904188	0.01928016		Cyp2d10	cytochrome P450, family 2, subfamily d, polypeptide 10

Table SVIII. KEGG analysis of the DEPs in DbCM compared to CK.

class_A	class_B	Term	ID	Input number	Background number	P-Value	Input
Human Diseases	Immune diseases	Systemic lupus erythematosus	mmu05322	10	149	3.39E-10	ENSMUSG00000100210|ENSMUSG00000074403|ENSMUSG00000099583|ENSMUSG00000081058|ENSMUSG00000069267|ENSMUSG00000093769|ENSMUSG00000069273|ENSMUSG00000035031|ENSMUSG00000073421|ENSMUSG00000069310
Human Diseases	Substance dependence	Alcoholism	mmu05034	10	204	6.04E-09	ENSMUSG00000100210|ENSMUSG00000099583|ENSMUSG00000040147|ENSMUSG00000081058|ENSMUSG00000069267|ENSMUSG00000093769|ENSMUSG00000069273|ENSMUSG00000074403|ENSMUSG00000004936|ENSMUSG00000069310
Human Diseases	Cancers: Overview	Transcriptional misregulation in cancer	mmu05202	9	180	3.03E-08	ENSMUSG00000100210|ENSMUSG00000048416|ENSMUSG00000099583|ENSMUSG00000081058|ENSMUSG00000069267|ENSMUSG00000093769|ENSMUSG00000069273|ENSMUSG00000074403|ENSMUSG00000069310
Cellular Processes	Transport and catabolism	Peroxisome	mmu04146	7	83	3.79E-08	ENSMUSG00000028603|ENSMUSG00000027187|ENSMUSG00000022040|ENSMUSG00000003623|ENSMUSG00000020777|ENSMUSG00000031767|ENSMUSG00000053898
Metabolism	Global and overview maps	Metabolic pathways	mmu01100	20	1298	5.52E-08	ENSMUSG00000059447|ENSMUSG00000028603|ENSMUSG00000021456|ENSMUSG00000040147|ENSMUSG00000022040|ENSMUSG00000019326|ENSMUSG00000000326|ENSMUSG00000024525|ENSMUSG00000027875|ENSMUSG00000061838|ENSMUSG00000071708|ENSMUSG00000021048|ENSMUSG00000020777|ENSMUSG00000039682|ENSMUSG00000072949|ENSMUSG00000021226|ENSMUSG00000032315|ENSMUSG00000017715|ENSMUSG00000003526|ENSMUSG00000030682
Organismal Systems	Endocrine system	PPAR signaling pathway	mmu03320	6	85	9.83E-07	ENSMUSG00000028603|ENSMUSG00000027875|ENSMUSG00000011305|ENSMUSG00000020777|ENSMUSG00000024900|EN

							SMUSG00000062515
Metabolism	Amino acid metabolism	Tryptophan metabolism	mmu00380	4	47	3.46E-05	ENSMUSG00000032315|ENSMUSG00000040147|ENSMUSG00000003477|ENSMUSG00000027187
Metabolism	Amino acid metabolism	Arginine and proline metabolism	mmu00330	4	51	4.66E-05	ENSMUSG00000071708|ENSMUSG00000040147|ENSMUSG00000039682|ENSMUSG00000003526
Metabolism	Metabolism of other amino acids	Glutathione metabolism	mmu00480	4	57	7.02E-05	ENSMUSG00000071708|ENSMUSG00000004035|ENSMUSG00000058135|ENSMUSG00000039682
Organismal Systems	Circulatory system	Vascular smooth muscle contraction	mmu04270	5	128	0.000122047	ENSMUSG00000004936|ENSMUSG00000035783|ENSMUSG00000018830|ENSMUSG00000019254|ENSMUSG00000090841
Metabolism	Lipid metabolism	Fatty acid elongation	mmu00062	3	27	0.000170076	ENSMUSG00000021226|ENSMUSG00000072949|ENSMUSG00000059447
Metabolism	Lipid metabolism	Biosynthesis of unsaturated fatty acids	mmu01040	3	28	0.000187806	ENSMUSG00000021226|ENSMUSG00000072949|ENSMUSG00000020777
Organismal Systems	Endocrine system	Oxytocin signaling pathway	mmu04921	5	158	0.000314151	ENSMUSG00000004936|ENSMUSG00000020122|ENSMUSG00000019254|ENSMUSG00000090841|ENSMUSG00000028944
Organismal Systems	Immune system	Complement and coagulation cascades	mmu04610	4	87	0.00033087	ENSMUSG00000092511|ENSMUSG00000059481|ENSMUSG00000035031|ENSMUSG00000072849
Human Diseases	Cancers: Overview	Chemical carcinogenesis	mmu05204	4	93	0.000421887	ENSMUSG00000058135|ENSMUSG00000004035|ENSMUSG00000030711|ENSMUSG00000032315
Metabolism	Amino acid metabolism	Tyrosine metabolism	mmu00350	3	40	0.000499671	ENSMUSG00000019326|ENSMUSG00000040147|ENSMUSG00000000326
Environmental Information	Signal transduction	HIF-1 signaling pathway	mmu04066	4	110	0.000775272	ENSMUSG00000004936|ENSMUSG00000020122|ENSMUSG00000041616|ENSMUSG00000022797

Processing							
Metabolism	Lipid metabolism	Fatty acid degradation	mmu00071	3	49	0.000873997	ENSMUSG00000024900|ENSMUSG00000020777|ENSMUSG00000059447
Metabolism	Global and overview maps	Fatty acid metabolism	mmu01212	3	52	0.00102943	ENSMUSG00000024900|ENSMUSG00000020777|ENSMUSG00000059447
Human Diseases	Infectious diseases: Bacterial	Staphylococcus aureus infection	mmu05150	3	53	0.001084864	ENSMUSG00000092511|ENSMUSG00000073421|ENSMUSG00000059481
Environmental Information Processing	Signal transduction	FoxO signaling pathway	mmu04068	4	135	0.001615069	ENSMUSG00000004936|ENSMUSG00000020122|ENSMUSG00000028944|ENSMUSG00000027187
Metabolism	Xenobiotics biodegradation and metabolism	Metabolism of xenobiotics by cytochrome P450	mmu00980	3	65	0.001900942	ENSMUSG00000058135|ENSMUSG00000004035|ENSMUSG00000032315
Human Diseases	Cancers: Overview	Central carbon metabolism in cancer	mmu05230	3	66	0.001982178	ENSMUSG00000004936|ENSMUSG00000020122|ENSMUSG00000025486
Metabolism	Xenobiotics biodegradation and metabolism	Drug metabolism - cytochrome P450	mmu00982	3	67	0.002065546	ENSMUSG00000004035|ENSMUSG00000040147|ENSMUSG00000058135
Metabolism	Energy metabolism	Nitrogen metabolism	mmu00910	2	17	0.002092773	ENSMUSG00000027559|ENSMUSG00000000805
Metabolism	Amino acid metabolism	Phenylalanine metabolism	mmu00360	2	24	0.00391278	ENSMUSG00000019326|ENSMUSG00000040147
Cellular Processes	Cellular community - eukaryotes	Gap junction	mmu04540	3	86	0.004078646	ENSMUSG00000004936|ENSMUSG00000020122|ENSMUSG00000001525
Metabolism	Lipid metabolism	Steroid hormone	mmu00140	3	87	0.00420835	ENSMUSG00000061740|ENSMUSG00000032315|ENSMUSG00000000326

		biosynthesis					
Organismal Systems	Development	Dorso-ventral axis formation	mmu04320	2	26	0.004529612	ENSMUSG00000004936|ENSMUSG00000020122
Human Diseases	Cancers: Specific types	Thyroid cancer	mmu05216	2	29	0.005533184	ENSMUSG00000004936|ENSMUSG00000006005
Metabolism	Metabolism of other amino acids	beta-Alanine metabolism	mmu00410	2	33	0.007014235	ENSMUSG00000071708|ENSMUSG00000019326
Human Diseases	Neurodegenerative diseases	Prion diseases	mmu05020	2	34	0.007409518	ENSMUSG00000004936|ENSMUSG00000035031
Human Diseases	Endocrine and metabolic diseases	Insulin resistance	mmu04931	3	111	0.008094679	ENSMUSG00000028944|ENSMUSG00000024900|ENSMUSG00000033083
Cellular Processes	Cell motility	Regulation of actin cytoskeleton	mmu04810	4	218	0.008511253	ENSMUSG00000004936|ENSMUSG00000020122|ENSMUSG00000019254|ENSMUSG00000026879
Metabolism	Global and overview maps	Carbon metabolism	mmu01200	3	118	0.009520465	ENSMUSG00000061838|ENSMUSG00000021456|ENSMUSG00000027187
Metabolism	Amino acid metabolism	Glycine, serine and threonine metabolism	mmu00260	2	41	0.010448342	ENSMUSG00000019326|ENSMUSG00000040147
Human Diseases	Cancers: Specific types	Bladder cancer	mmu05219	2	41	0.010448342	ENSMUSG00000004936|ENSMUSG00000020122
Environmental Information Processing	Signal transduction	AMPK signaling pathway	mmu04152	3	129	0.012039565	ENSMUSG00000028944|ENSMUSG00000024900|ENSMUSG00000021456
Organismal Systems	Nervous system	Serotonergic synapse	mmu04726	3	133	0.013041415	ENSMUSG00000004936|ENSMUSG00000061740|ENSMUSG00000040147
Organismal Systems	Endocrine system	Insulin signaling pathway	mmu04910	3	142	0.015465233	ENSMUSG00000004936|ENSMUSG00000028944|ENSMUSG00000021456

Human Diseases	Cancers: Specific types	Endometrial cancer	mmu05213	2	52	0.016139381	ENSMUSG00000004936|ENSMUSG00000020122
Metabolism	Amino acid metabolism	Valine, leucine and isoleucine degradation	mmu00280	2	56	0.018470895	ENSMUSG00000027875|ENSMUSG00000059447
Human Diseases	Cancers: Specific types	Non-small cell lung cancer	mmu05223	2	56	0.018470895	ENSMUSG00000004936|ENSMUSG00000020122
Organismal Systems	Endocrine system	Ovarian steroidogenesis	mmu04913	2	58	0.019686749	ENSMUSG00000021226|ENSMUSG00000032315
Organismal Systems	Aging	Longevity regulating pathway - multiple species	mmu04213	2	64	0.023528382	ENSMUSG00000028944|ENSMUSG00000027187
Human Diseases	Cancers: Specific types	Glioma	mmu05214	2	65	0.024196276	ENSMUSG00000004936|ENSMUSG00000020122
Genetic Information Processing	Translation	RNA transport	mmu03013	3	170	0.024528659	ENSMUSG00000006005|ENSMUSG00000069049|ENSMUSG00000067194
Human Diseases	Cancers: Specific types	Pancreatic cancer	mmu05212	2	66	0.024871892	ENSMUSG00000004936|ENSMUSG00000020122
Human Diseases	Infectious diseases: Viral	Influenza A	mmu05164	3	172	0.025264744	ENSMUSG00000004936|ENSMUSG00000073421|ENSMUSG00000059481
Metabolism	Carbohydrate metabolism	Inositol phosphate metabolism	mmu00562	2	70	0.027650321	ENSMUSG00000030682|ENSMUSG00000024525
Human Diseases	Cancers: Specific types	Melanoma	mmu05218	2	72	0.029084244	ENSMUSG00000004936|ENSMUSG00000020122
Organismal Systems	Endocrine system	Adipocytokine signaling	mmu04920	2	73	0.029812167	ENSMUSG00000028944|ENSMUSG00000024900

		pathway					
Human Diseases	Endocrine and metabolic diseases	Type I diabetes mellitus	mmu04940	2	77	0.032795534	ENSMUSG00000037852|ENSMUSG00000073421
Cellular Processes	Transport and catabolism	Phagosome	mmu04145	3	192	0.033273867	ENSMUSG00000073421|ENSMUSG00000022797|ENSMUSG00000001525
Human Diseases	Drug resistance: Antineoplastic	Platinum drug resistance	mmu01524	2	78	0.033558994	ENSMUSG00000004035|ENSMUSG00000058135
Organismal Systems	Circulatory system	Cardiac muscle contraction	mmu04260	2	80	0.035106644	ENSMUSG00000061086|ENSMUSG00000053093
Human Diseases	Drug resistance: Antineoplastic	EGFR tyrosine kinase inhibitor resistance	mmu01521	2	82	0.036681542	ENSMUSG00000004936|ENSMUSG00000020122
Cellular Processes	Cellular community - eukaryotes	Focal adhesion	mmu04510	3	203	0.038176935	ENSMUSG00000004936|ENSMUSG00000020122|ENSMUSG00000019254
Human Diseases	Cardiovascular diseases	Hypertrophic cardiomyopathy (HCM)	mmu05410	2	84	0.038283226	ENSMUSG00000028944|ENSMUSG00000053093
Human Diseases	Cancers: Overview	Proteoglycans in cancer	mmu05205	3	208	0.040520881	ENSMUSG00000004936|ENSMUSG00000020122|ENSMUSG00000019254
Environmental Information Processing	Signal transduction	ErbB signaling pathway	mmu04012	2	87	0.040734976	ENSMUSG00000004936|ENSMUSG00000020122
Organismal Systems	Endocrine system	GnRH signaling pathway	mmu04912	2	88	0.041565127	ENSMUSG00000004936|ENSMUSG00000020122
Metabolism	Metabolism of cofactors and vitamins	Ubiquinone and other terpenoid-quinone	mmu00130	1	11	0.042151076	ENSMUSG00000003849

		biosynthesis					
Metabolism	Lipid metabolism	Synthesis and degradation of ketone bodies	mmu00072	1	11	0.042151076	ENSMUSG00000027875
Human Diseases	Cancers: Specific types	Prostate cancer	mmu05215	2	89	0.042401634	ENSMUSG00000004936|ENSMUSG00000020122
Organismal Systems	Immune system	Fc gamma R-mediated phagocytosis	mmu04666	2	90	0.043244443	ENSMUSG00000004936|ENSMUSG00000026879
Metabolism	Lipid metabolism	Glycerophospholipid metabolism	mmu00564	2	94	0.046677598	ENSMUSG00000030682|ENSMUSG00000017715
Human Diseases	Drug resistance: Antineoplastic	Endocrine resistance	mmu01522	2	95	0.047551094	ENSMUSG00000004936|ENSMUSG00000020122
Organismal Systems	Aging	Longevity regulating pathway	mmu04211	2	96	0.048430565	ENSMUSG00000028944|ENSMUSG00000027187
Human Diseases	Cardiovascular diseases	Viral myocarditis	mmu05416	2	96	0.048430565	ENSMUSG00000073421|ENSMUSG00000053093
Environmental Information Processing	Signal transduction	Phosphatidylinositol signaling system	mmu04070	2	97	0.049315958	ENSMUSG00000030682|ENSMUSG00000024525
Organismal Systems	Immune system	Antigen processing and presentation	mmu04612	2	98	0.050207221	ENSMUSG00000073421|ENSMUSG00000024308
Organismal Systems	Endocrine system	Estrogen signaling pathway	mmu04915	2	98	0.050207221	ENSMUSG00000004936|ENSMUSG00000020122
Human Diseases	Cancers: Overview	Choline metabolism in cancer	mmu05231	2	101	0.052915695	ENSMUSG00000004936|ENSMUSG00000020122

Organismal Systems	Endocrine system	Glucagon signaling pathway	mmu04922	2	102	0.053829909	ENSMUSG00000028944|ENSMUSG00000024900
Metabolism	Lipid metabolism	Primary bile acid biosynthesis	mmu00120	1	16	0.059188981	ENSMUSG00000028603
Metabolism	Metabolism of other amino acids	Selenocompound metabolism	mmu00450	1	17	0.062560281	ENSMUSG00000003477
Human Diseases	Infectious diseases: Parasitic	Toxoplasmosis	mmu05145	2	113	0.064245281	ENSMUSG00000073421|ENSMUSG00000046879
Organismal Systems	Endocrine system	Thyroid hormone signaling pathway	mmu04919	2	117	0.068187943	ENSMUSG00000004936|ENSMUSG00000033083
Metabolism	Metabolism of cofactors and vitamins	One carbon pool by folate	mmu00670	1	19	0.069266941	ENSMUSG00000021048
Cellular Processes	Transport and catabolism	Lysosome	mmu04142	2	124	0.075273294	ENSMUSG00000004207|ENSMUSG00000030894
Organismal Systems	Excretory system	Proximal tubule bicarbonate reclamation	mmu04964	1	22	0.079237715	ENSMUSG00000000805
Metabolism	Metabolism of terpenoids and polyketides	Terpenoid backbone biosynthesis	mmu00900	1	24	0.085825909	ENSMUSG00000027875
Organismal Systems	Nervous system	Dopaminergic synapse	mmu04728	2	135	0.086851681	ENSMUSG00000040147|ENSMUSG00000000326
Metabolism	Lipid	alpha-Linolenic	mmu0	1	25	0.0891024	ENSMUSG00000020777

	metabolism	acid metabolism	0592			34	
Human Diseases	Immune diseases	Asthma	mmu0 5310	1	26	0.0923673	ENSMUSG00000073421
Metabolism	Amino acid metabolism	Histidine metabolism	mmu0 0340	1	26	0.0923673	ENSMUSG00000040147
Cellular Processes	Cellular community - eukaryotes	Tight junction	mmu0 4530	2	141	0.0933780 96	ENSMUSG00000018830|ENSMUSG00000053093
Metabolism	Carbohydrate metabolism	Butanoate metabolism	mmu0 0650	1	27	0.0956205 48	ENSMUSG00000027875
Environmental Information Processing	Signal transduction	Phospholipase D signaling pathway	mmu0 4072	2	146	0.0989220 17	ENSMUSG00000004936|ENSMUSG00000020122
Metabolism	Carbohydrate metabolism	Glyoxylate and dicarboxylate metabolism	mmu0 0630	1	30	0.1053109 91	ENSMUSG00000027187
Organismal Systems	Circulatory system	Adrenergic signaling in cardiomyocyte s	mmu0 4261	2	152	0.1056934 52	ENSMUSG00000061086|ENSMUSG00000053093
Metabolism	Carbohydrate metabolism	Pentose phosphate pathway	mmu0 0030	1	31	0.1085181 76	ENSMUSG00000021456
Organismal Systems	Environmental adaptation	Circadian rhythm	mmu0 4710	1	31	0.1085181 76	ENSMUSG00000028944
Metabolism	Carbohydrate metabolism	Propanoate metabolism	mmu0 0640	1	31	0.1085181 76	ENSMUSG00000061838
Genetic Information Processing	Folding, sorting and degradation	Protein export	mmu0 3060	1	31	0.1085181 76	ENSMUSG00000000959

Metabolism	Carbohydrate metabolism	Citrate cycle (TCA cycle)	mmu00020	1	32	0.111713945	ENSMUSG00000061838
Metabolism	Carbohydrate metabolism	Fructose and mannose metabolism	mmu00051	1	35	0.121233171	ENSMUSG00000021456
Environmental Information Processing	Signal transduction	cGMP-PKG signaling pathway	mmu04022	2	173	0.130297889	ENSMUSG00000004936|ENSMUSG00000053093
Environmental Information Processing	Signal transduction	PI3K-Akt signaling pathway	mmu04151	3	350	0.133701313	ENSMUSG00000004936|ENSMUSG00000020122|ENSMUSG00000057672
Organismal Systems	Immune system	Intestinal immune network for IgA production	mmu04672	1	44	0.149187403	ENSMUSG00000073421
Genetic Information Processing	Folding, sorting and degradation	Proteasome	mmu03050	1	45	0.152238405	ENSMUSG00000005625
Environmental Information Processing	Signal transduction	cAMP signaling pathway	mmu04024	2	198	0.161073204	ENSMUSG00000004936|ENSMUSG00000020777
Human Diseases	Substance dependence	Cocaine addiction	mmu05030	1	49	0.164334174	ENSMUSG00000040147
Metabolism	Amino acid metabolism	Cysteine and methionine metabolism	mmu00270	1	49	0.164334174	ENSMUSG00000071708
Human Diseases	Neurodegenerative diseases	Amyotrophic lateral sclerosis (ALS)	mmu05014	1	52	0.173293414	ENSMUSG00000027187
Human Diseases	Cancers: Overview	Pathways in cancer	mmu05200	3	398	0.174367982	ENSMUSG00000004936|ENSMUSG00000020122|ENSMUSG00000006005

Environmental Information Processing	Signal transduction	Rap1 signaling pathway	mmu04015	2	215	0.182684884	ENSMUSG00000004936|ENSMUSG00000020122
Human Diseases	Cancers: Specific types	Acute myeloid leukemia	mmu05221	1	57	0.188014049	ENSMUSG00000004936
Organismal Systems	Endocrine system	Regulation of lipolysis in adipocytes	mmu04923	1	57	0.188014049	ENSMUSG00000062515
Human Diseases	Immune diseases	Inflammatory bowel disease (IBD)	mmu05321	1	59	0.19382917	ENSMUSG00000073421
Environmental Information Processing	Signal transduction	VEGF signaling pathway	mmu04370	1	60	0.196721207	ENSMUSG00000004936
Organismal Systems	Nervous system	Long-term depression	mmu04730	1	61	0.199602943	ENSMUSG00000004936
Environmental Information Processing	Signal transduction	Ras signaling pathway	mmu04014	2	231	0.203388786	ENSMUSG00000004936|ENSMUSG00000020122
Human Diseases	Cancers: Specific types	Colorectal cancer	mmu05210	1	64	0.208186713	ENSMUSG00000004936
Organismal Systems	Nervous system	Long-term potentiation	mmu04720	1	66	0.213858388	ENSMUSG00000004936
Metabolism	Carbohydrate metabolism	Glycolysis / Gluconeogenesis	mmu00010	1	66	0.213858388	ENSMUSG00000021456
Human Diseases	Infectious diseases: Parasitic	Leishmaniasis	mmu05140	1	67	0.216679082	ENSMUSG00000073421
Human Diseases	Substance dependence	Amphetamine addiction	mmu05031	1	68	0.219489728	ENSMUSG00000040147

Human Diseases	Cancers: Specific types	Renal cell carcinoma	mmu0 5211	1	68	0.2194897 28	ENSMUSG00000004936
Organismal Systems	Immune system	Fc epsilon RI signaling pathway	mmu0 4664	1	69	0.2222903 61	ENSMUSG00000004936
Human Diseases	Immune diseases	Allograft rejection	mmu0 5330	1	72	0.2306325 38	ENSMUSG00000073421
Human Diseases	Immune diseases	Graft-versus-h ost disease	mmu0 5332	1	72	0.2306325 38	ENSMUSG00000073421
Human Diseases	Cancers: Specific types	Chronic myeloid leukemia	mmu0 5220	1	73	0.2333934 74	ENSMUSG00000004936
Environmental Information Processing	Signal transduction	MAPK signaling pathway	mmu0 4010	2	254	0.2335626 24	ENSMUSG00000004936|ENSMUSG00000020122
Organismal Systems	Endocrine system	Prolactin signaling pathway	mmu0 4917	1	74	0.2361445 72	ENSMUSG00000004936
Cellular Processes	Cellular community - eukaryotes	Adherens junction	mmu0 4520	1	74	0.2361445 72	ENSMUSG00000020122
Organismal Systems	Immune system	B cell receptor signaling pathway	mmu0 4662	1	75	0.2388858 69	ENSMUSG00000004936
Organismal Systems	Digestive system	Salivary secretion	mmu0 4970	1	78	0.2470512 93	ENSMUSG00000027447
Human Diseases	Infectious diseases: Bacterial	Salmonella infection	mmu0 5132	1	78	0.2470512 93	ENSMUSG00000057672
Human Diseases	Immune diseases	Rheumatoid arthritis	mmu0 5323	1	85	0.2657672 93	ENSMUSG00000073421

Human Diseases	Cancers: Overview	MicroRNAs in cancer	mmu05206	2	281	0.269285847	ENSMUSG00000004936|ENSMUSG00000020122
Organismal Systems	Immune system	Hematopoietic cell lineage	mmu04640	1	87	0.271029372	ENSMUSG00000022797
Human Diseases	Immune diseases	Autoimmune thyroid disease	mmu05320	1	87	0.271029372	ENSMUSG00000073421
Metabolism	Metabolism of cofactors and vitamins	Retinol metabolism	mmu00830	1	89	0.276254008	ENSMUSG00000032315
Metabolism	Lipid metabolism	Arachidonic acid metabolism	mmu00590	1	90	0.278852368	ENSMUSG00000022040
Human Diseases	Cardiovascular diseases	Dilated cardiomyopathy	mmu05414	1	90	0.278852368	ENSMUSG00000053093
Organismal Systems	Endocrine system	Progesterone-mediated oocyte maturation	mmu04914	1	90	0.278852368	ENSMUSG00000004936
Cellular Processes	Transport and catabolism	Endocytosis	mmu04144	2	294	0.286499814	ENSMUSG00000020122|ENSMUSG00000022797
Organismal Systems	Endocrine system	Melanogenesis	mmu04916	1	99	0.30182474	ENSMUSG00000004936
Organismal Systems	Immune system	Toll-like receptor signaling pathway	mmu04620	1	101	0.30683021	ENSMUSG00000004936
Organismal Systems	Immune system	T cell receptor signaling pathway	mmu04660	1	105	0.316734512	ENSMUSG00000004936

Human Diseases	Infectious diseases: Parasitic	Amoebiasis	mmu05146	1	111	0.331328123	ENSMUSG00000035031
Environmental Information Processing	Signal transduction	TNF signaling pathway	mmu04668	1	112	0.333730123	ENSMUSG00000004936
Organismal Systems	Nervous system	Cholinergic synapse	mmu04725	1	113	0.336123557	ENSMUSG00000004936
Cellular Processes	Cell growth and death	Oocyte meiosis	mmu04114	1	116	0.34325276	ENSMUSG00000004936
Organismal Systems	Immune system	Natural killer cell mediated cytotoxicity	mmu04650	1	121	0.354966244	ENSMUSG00000004936
Organismal Systems	Nervous system	Neurotrophin signaling pathway	mmu04722	1	122	0.35728393	ENSMUSG00000004936
Environmental Information Processing	Signal transduction	Sphingolipid signaling pathway	mmu04071	1	125	0.364187498	ENSMUSG00000004936
Organismal Systems	Development	Osteoclast differentiation	mmu04380	1	133	0.38223918	ENSMUSG00000004936
Human Diseases	Infectious diseases: Viral	Hepatitis C	mmu05160	1	137	0.391073272	ENSMUSG00000020122
Cellular Processes	Cell growth and death	Apoptosis	mmu04210	1	139	0.395443169	ENSMUSG00000004936
Cellular Processes	Cellular community - eukaryotes	Signaling pathways regulating pluripotency of stem cells	mmu04550	1	140	0.397616428	ENSMUSG00000004936
Human	Infectious	Hepatitis B	mmu0	1	146	0.4104940	ENSMUSG00000004936

Diseases	diseases: Viral		5161			55	
Human Diseases	Neurodegenerative diseases	Parkinson's disease	mmu05012	1	149	0.416829996	ENSMUSG00000029223
Environmental Information Processing	Signal transduction	mTOR signaling pathway	mmu04150	1	156	0.431352157	ENSMUSG00000004936
Human Diseases	Endocrine and metabolic diseases	Non-alcoholic fatty liver disease (NAFLD)	mmu04932	1	157	0.433397229	ENSMUSG00000028944
Genetic Information Processing	Folding, sorting and degradation	Protein processing in endoplasmic reticulum	mmu04141	1	167	0.453450577	ENSMUSG00000039474
Environmental Information Processing	Signaling molecules and interaction	Cell adhesion molecules (CAMs)	mmu04514	1	176	0.470895273	ENSMUSG00000073421
Human Diseases	Infectious diseases: Bacterial	Tuberculosis	mmu05152	1	179	0.47658643	ENSMUSG00000073421
Environmental Information Processing	Signal transduction	Calcium signaling pathway	mmu04020	1	182	0.482216807	ENSMUSG00000020122
Organismal Systems	Immune system	Chemokine signaling pathway	mmu04062	1	198	0.511245292	ENSMUSG00000004936
Human Diseases	Infectious diseases: Viral	Herpes simplex infection	mmu05168	1	224	0.555009088	ENSMUSG00000073421
Human Diseases	Infectious diseases: Viral	Epstein-Barr virus infection	mmu05169	1	232	0.567675069	ENSMUSG00000005625

Human Diseases	Cancers: Overview	Viral carcinogenesis	mmu05203	1	245	0.587498222	ENSMUSG00000026879
Environmental Information Processing	Signaling molecules and interaction	Cytokine-cytokine receptor interaction	mmu04060	1	265	0.616247095	ENSMUSG00000020122
Environmental Information Processing	Signaling molecules and interaction	Neuroactive ligand-receptor interaction	mmu04080	1	285	0.643005757	ENSMUSG00000059481
Human Diseases	Infectious diseases: Viral	HTLV-I infection	mmu05166	1	295	0.655681896	ENSMUSG00000073421

Table SIX. The DEPs in the metabolic related pathways of DbCM / CK.

class_A	class_B	Term	ID	Inputnumber	Backgroundnumber	P_Value	Input
Metabolism	Metabolism of other amino acids	Selenocompound metabolism	mmu00450	1	17	0.062560281	ENSMUSG00000003477
Metabolism	Metabolism of cofactors and vitamins	Ubiquinone and other terpenoid-quinone biosynthesis	mmu00130	1	11	0.042151076	ENSMUSG00000003849
Metabolism	Lipid metabolism	alpha-Linolenic acid metabolism	mmu00592	1	25	0.089102434	ENSMUSG00000020777
Metabolism	Metabolism of cofactors and vitamins	One carbon pool by folate	mmu00670	1	19	0.069266941	ENSMUSG00000021048
Metabolism	Carbohydrate metabolism	Pentose phosphate pathway	mmu00030	1	31	0.108518176	ENSMUSG00000021456
Metabolism	Carbohydrate metabolism	Fructose and mannose metabolism	mmu00051	1	35	0.121233171	ENSMUSG00000021456
Metabolism	Carbohydrate metabolism	Glycolysis / Gluconeogenesis	mmu00010	1	66	0.213858388	ENSMUSG00000021456
Metabolism	Lipid metabolism	Arachidonic acid metabolism	mmu00590	1	90	0.278852368	ENSMUSG00000022040
Metabolism	Carbohydrate metabolism	Glyoxylate and dicarboxylate metabolism	mmu00630	1	30	0.105310991	ENSMUSG00000027187
Metabolism	Carbohydrate metabolism	Butanoate metabolism	mmu00650	1	27	0.095620548	ENSMUSG00000027875
Metabolism	Lipid metabolism	Synthesis and degradation of ketone bodies	mmu00072	1	11	0.042151076	ENSMUSG00000027875

Metabolism	Metabolism of terpenoids and polyketides	Terpenoid backbone biosynthesis	mmu00900	1	24	0.085825909	ENSMUSG00000027875
Metabolism	Lipid metabolism	Primary bile acid biosynthesis	mmu00120	1	16	0.059188981	ENSMUSG00000028603
Metabolism	Metabolism of cofactors and vitamins	Retinol metabolism	mmu00830	1	89	0.276254008	ENSMUSG00000032315
Metabolism	Amino acid metabolism	Histidine metabolism	mmu00340	1	26	0.0923673	ENSMUSG00000040147
Metabolism	Carbohydrate metabolism	Propanoate metabolism	mmu00640	1	31	0.108518176	ENSMUSG00000061838
Metabolism	Carbohydrate metabolism	Citrate cycle (TCA cycle)	mmu00020	1	32	0.111713945	ENSMUSG00000061838
Metabolism	Amino acid metabolism	Cysteine and methionine metabolism	mmu00270	1	49	0.164334174	ENSMUSG00000071708
Metabolism	Amino acid metabolism	Phenylalanine metabolism	mmu00360	2	24	0.00391278	ENSMUSG00000019326|ENSMUSG00000040147
Metabolism	Amino acid metabolism	Glycine, serine and threonine metabolism	mmu00260	2	41	0.010448342	ENSMUSG00000019326|ENSMUSG00000040147
Metabolism	Energy metabolism	Nitrogen metabolism	mmu00910	2	17	0.002092773	ENSMUSG00000027559|ENSMUSG00000000805
Metabolism	Amino acid metabolism	Valine, leucine and isoleucine degradation	mmu00280	2	56	0.018470895	ENSMUSG00000027875|ENSMUSG00000059447
Metabolism	Lipid metabolism	Glycerophospholipid metabolism	mmu00564	2	94	0.046677598	ENSMUSG00000030682|ENSMUSG00000017715
Metabolism	Carbohydrate metabolism	Inositol phosphate metabolism	mmu00562	2	70	0.027650321	ENSMUSG00000030682|ENSMUSG00000024525
Metabolism	Metabolism of other amino acids	beta-Alanine metabolism	mmu00410	2	33	0.007014235	ENSMUSG00000071708|ENSMUSG00000019326

Metabolism	Xenobiotics biodegradation and metabolism	Drug metabolism - cytochrome P450	mmu00982	3	67	0.002065546	ENSMUSG00000004035|ENSMUSG00000040147|ENSMUSG00000058135
Metabolism	Amino acid metabolism	Tyrosine metabolism	mmu00350	3	40	0.000499671	ENSMUSG00000019326|ENSMUSG00000040147|ENSMUSG00000000326
Metabolism	Lipid metabolism	Biosynthesis of unsaturated fatty acids	mmu01040	3	28	0.000187806	ENSMUSG00000021226|ENSMUSG00000072949|ENSMUSG00000020777
Metabolism	Lipid metabolism	Fatty acid elongation	mmu00062	3	27	0.000170076	ENSMUSG00000021226|ENSMUSG00000072949|ENSMUSG00000059447
Metabolism	Global and overview maps	Fatty acid metabolism	mmu01212	3	52	0.00102943	ENSMUSG00000024900|ENSMUSG00000020777|ENSMUSG00000059447
Metabolism	Lipid metabolism	Fatty acid degradation	mmu00071	3	49	0.000873997	ENSMUSG00000024900|ENSMUSG00000020777|ENSMUSG00000059447
Metabolism	Xenobiotics biodegradation and metabolism	Metabolism of xenobiotics by cytochrome P450	mmu00980	3	65	0.001900942	ENSMUSG00000058135|ENSMUSG00000004035|ENSMUSG00000032315
Metabolism	Lipid metabolism	Steroid hormone biosynthesis	mmu00140	3	87	0.00420835	ENSMUSG00000061740|ENSMUSG00000032315|ENSMUSG00000000326
Metabolism	Global and overview maps	Carbon metabolism	mmu01200	3	118	0.009520465	ENSMUSG00000061838|ENSMUSG00000021456|ENSMUSG00000027187
Metabolism	Amino acid metabolism	Tryptophan metabolism	mmu00380	4	47	3.46E-05	ENSMUSG00000032315|ENSMUSG00000040147|ENSMUSG00000003477|ENSMUSG00000027187
Metabolism	Metabolism of other amino acids	Glutathione metabolism	mmu00480	4	57	7.02E-05	ENSMUSG00000071708|ENSMUSG00000004035|ENSMUSG00000058135|ENSMUSG00000039682
Metabolism	Amino acid metabolism	Arginine and proline metabolism	mmu00330	4	51	4.66E-05	ENSMUSG00000071708|ENSMUSG00000040147|ENSMUSG00000039682|ENSMUSG00000003526
