# Supplementary figures and images for: Transcriptomics Coupled to Proteomics Reveals Novel Targets for the Protective Role of Spermine in Diabetic Cardiomyopathy
Source: Oxid Med Cell Longev. 2022 Apr 9;2022:5909378. doi: 10.1155/2022/5909378 (PMC9013312; doi:10.1155/2022/5909378)

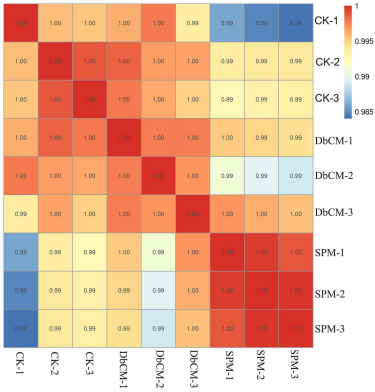

Supplement: Supplementary Materials — Figure S1: correlation analysis of myocardial tissue samples in the CK, DbCM, and SPM groups. Figure S2: KEGG pathway analysis of the DEGs in DbCM/CK and SPM/DbCM. (A) Enrichment pathway of DEGs in DbCM compared to CK. (B) Enrichment pathway of DEGs in SPM compared to DbCM. Figure S3: GSEA plot showing most enriched gene sets of all detected genes in DbCM and SPM mice. (A) The top five significant enriched gene sets related to metabolic pathway in the DbCM group. (B) The top five significant enriched gene sets related to metabolic pathway in the SPM group. Figure S4: KEGG analysis of the DEPs in DbCM/CK. Table S1: biochemical parameters in CK, DbCM, and SPM mouse. Table S2: heart function-related indexes in CK, DbCM, and SPM mouse. Table S3: Venn analysis identified 174 genes that differentially expressed in DbCM but reversally regulated by SPM. Table S4: KEGG analysis of the DEGs in DbCM compared to CK. Table S5: KEGG analysis of the DEGs in SPM compared to DbCM. Table S6: the DEGs in the metabolic related pathways of DbCM/CK. Table S7: the DEGs in the metabolic related pathways of SPM/DbCM. Table S8: KEGG analysis of the DEPs in DbCM compared to CK. Table S9: the DEPs in the metabolic related pathways of DbCM/CK. [file 5909378.f1.zip › Figure S1.pdf]

A

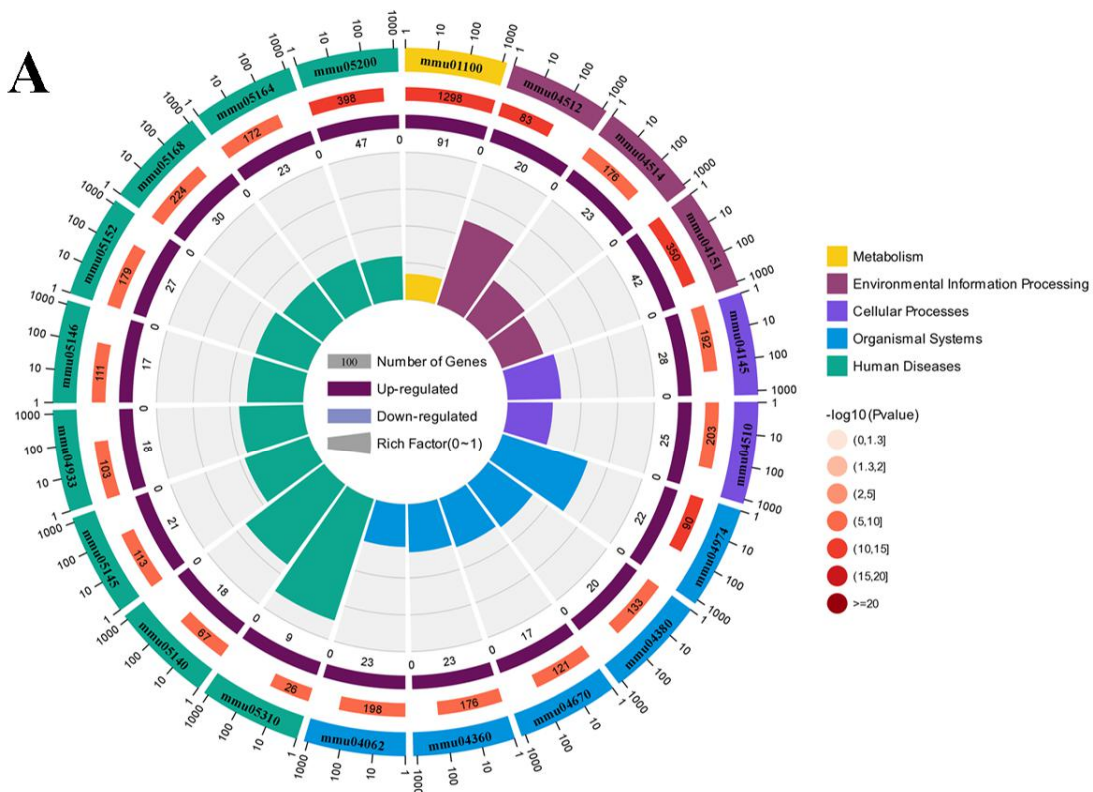

B

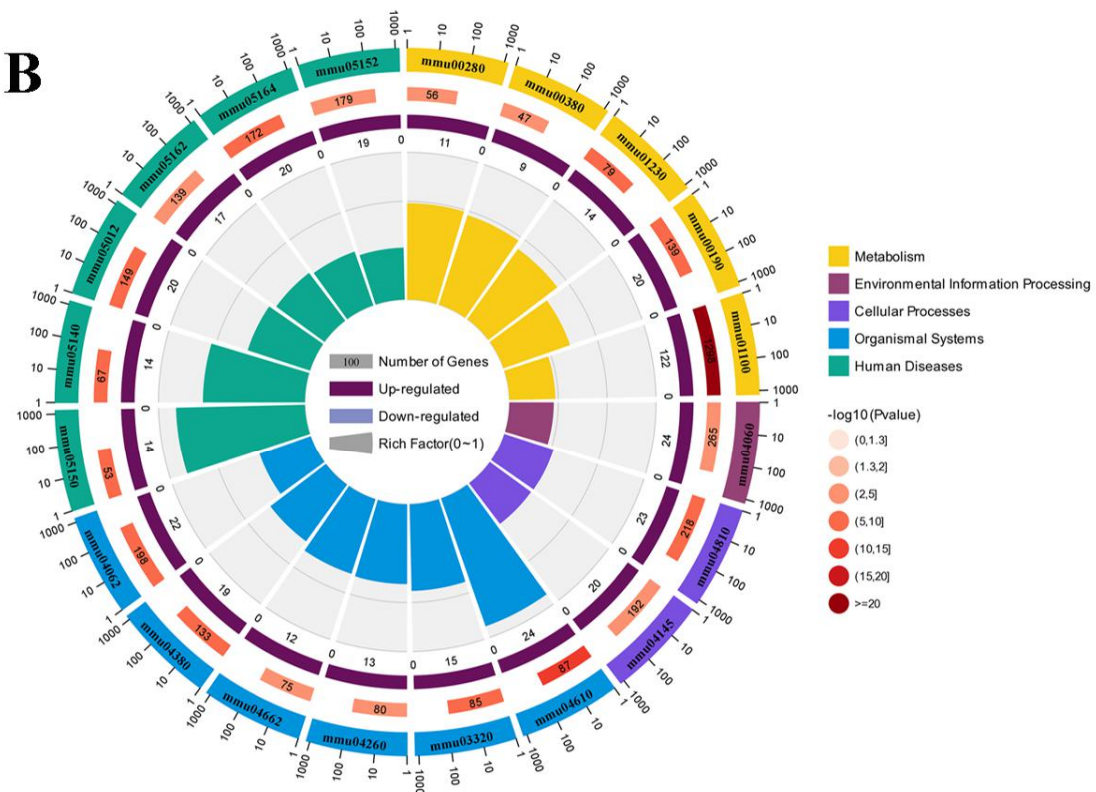

Supplement: Supplementary Materials — Figure S1: correlation analysis of myocardial tissue samples in the CK, DbCM, and SPM groups. Figure S2: KEGG pathway analysis of the DEGs in DbCM/CK and SPM/DbCM. (A) Enrichment pathway of DEGs in DbCM compared to CK. (B) Enrichment pathway of DEGs in SPM compared to DbCM. Figure S3: GSEA plot showing most enriched gene sets of all detected genes in DbCM and SPM mice. (A) The top five significant enriched gene sets related to metabolic pathway in the DbCM group. (B) The top five significant enriched gene sets related to metabolic pathway in the SPM group. Figure S4: KEGG analysis of the DEPs in DbCM/CK. Table S1: biochemical parameters in CK, DbCM, and SPM mouse. Table S2: heart function-related indexes in CK, DbCM, and SPM mouse. Table S3: Venn analysis identified 174 genes that differentially expressed in DbCM but reversally regulated by SPM. Table S4: KEGG analysis of the DEGs in DbCM compared to CK. Table S5: KEGG analysis of the DEGs in SPM compared to DbCM. Table S6: the DEGs in the metabolic related pathways of DbCM/CK. Table S7: the DEGs in the metabolic related pathways of SPM/DbCM. Table S8: KEGG analysis of the DEPs in DbCM compared to CK. Table S9: the DEPs in the metabolic related pathways of DbCM/CK. [file 5909378.f1.zip › Figure S2.pdf]

A

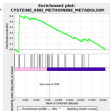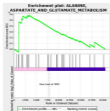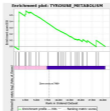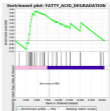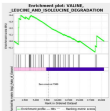

B

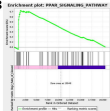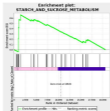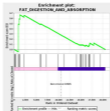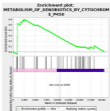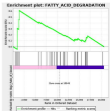

Supplement: Supplementary Materials — Figure S1: correlation analysis of myocardial tissue samples in the CK, DbCM, and SPM groups. Figure S2: KEGG pathway analysis of the DEGs in DbCM/CK and SPM/DbCM. (A) Enrichment pathway of DEGs in DbCM compared to CK. (B) Enrichment pathway of DEGs in SPM compared to DbCM. Figure S3: GSEA plot showing most enriched gene sets of all detected genes in DbCM and SPM mice. (A) The top five significant enriched gene sets related to metabolic pathway in the DbCM group. (B) The top five significant enriched gene sets related to metabolic pathway in the SPM group. Figure S4: KEGG analysis of the DEPs in DbCM/CK. Table S1: biochemical parameters in CK, DbCM, and SPM mouse. Table S2: heart function-related indexes in CK, DbCM, and SPM mouse. Table S3: Venn analysis identified 174 genes that differentially expressed in DbCM but reversally regulated by SPM. Table S4: KEGG analysis of the DEGs in DbCM compared to CK. Table S5: KEGG analysis of the DEGs in SPM compared to DbCM. Table S6: the DEGs in the metabolic related pathways of DbCM/CK. Table S7: the DEGs in the metabolic related pathways of SPM/DbCM. Table S8: KEGG analysis of the DEPs in DbCM compared to CK. Table S9: the DEPs in the metabolic related pathways of DbCM/CK. [file 5909378.f1.zip › Figure S3.pdf]

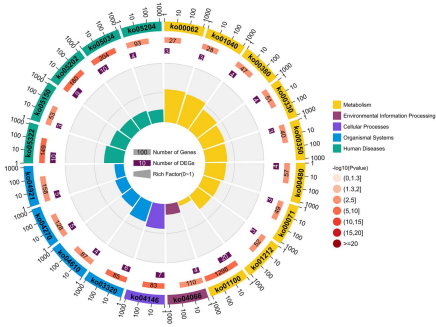

Supplement: Supplementary Materials — Figure S1: correlation analysis of myocardial tissue samples in the CK, DbCM, and SPM groups. Figure S2: KEGG pathway analysis of the DEGs in DbCM/CK and SPM/DbCM. (A) Enrichment pathway of DEGs in DbCM compared to CK. (B) Enrichment pathway of DEGs in SPM compared to DbCM. Figure S3: GSEA plot showing most enriched gene sets of all detected genes in DbCM and SPM mice. (A) The top five significant enriched gene sets related to metabolic pathway in the DbCM group. (B) The top five significant enriched gene sets related to metabolic pathway in the SPM group. Figure S4: KEGG analysis of the DEPs in DbCM/CK. Table S1: biochemical parameters in CK, DbCM, and SPM mouse. Table S2: heart function-related indexes in CK, DbCM, and SPM mouse. Table S3: Venn analysis identified 174 genes that differentially expressed in DbCM but reversally regulated by SPM. Table S4: KEGG analysis of the DEGs in DbCM compared to CK. Table S5: KEGG analysis of the DEGs in SPM compared to DbCM. Table S6: the DEGs in the metabolic related pathways of DbCM/CK. Table S7: the DEGs in the metabolic related pathways of SPM/DbCM. Table S8: KEGG analysis of the DEPs in DbCM compared to CK. Table S9: the DEPs in the metabolic related pathways of DbCM/CK. [file 5909378.f1.zip › Figure S4.pdf]
